# Pattern to Knowledge: Deep Knowledge-Directed Machine Learning for Residue-Residue Interaction Prediction

**DOI:** 10.1038/s41598-018-32834-z

**Published:** 2018-10-04

**Authors:** Andrew K. C. Wong, Ho Yin Sze-To, Gary L. Johanning

**Affiliations:** 1 Department of Systems Design Engineering, University of Waterloo, 200 University Avenue West, Waterloo, N2L 3G1 Ontario Canada; 2 Biosciences Division, SRI International, 333 Ravenswood Ave, Menlo Park, CA USA

## Abstract

Residue-residue close contact (R2R-C) data procured from three-dimensional protein-protein interaction (PPI) experiments is currently used for predicting residue-residue interaction (R2R-I) in PPI. However, due to complex physiochemical environments, R2R-I incidences, facilitated by multiple factors, are usually entangled in the source environment and masked in the acquired data. Here we present a novel method, P2K (Pattern to Knowledge), to disentangle R2R-I patterns and render much succinct discriminative information expressed in different specific R2R-I statistical/functional spaces. Since such knowledge is not visible in the data acquired, we refer to it as deep knowledge. Leveraging the deep knowledge discovered to construct machine learning models for sequence-based R2R-I prediction, without trial-and-error combination of the features over external knowledge of sequences, our R2R-I predictor was validated for its effectiveness under stringent leave-one-complex-out-alone cross-validation in a benchmark dataset, and was surprisingly demonstrated to perform better than an existing sequence-based R2R-I predictor by 28% (p: 1.9E-08). P2K is accessible via our web server on https://p2k.uwaterloo.ca.

## Introduction

In Protein-Protein interaction (PPI), residue-residue interaction (R2R-I) prediction refers to the identification of pairs of interacting residues, usually under close contact, residing on separate interacting proteins. A high-level definition of R2R-I prediction is: *“Given two proteins A and B, predict which residues in protein A interact with which residues in protein B, assuming proteins A and B can interact”*^1,2^. R2R-I prediction is critical, as it enhances our scientific understanding of PPI and furnishes potential targets for inhibiting PPI^3^. One example is the use of small molecules to inhibit the interaction between p53 and MDM2^4^, a potential cancer treatment. Despite its importance, R2R-I prediction in PPI is still hampered by expensive, labor-intensive and time-consuming experiments, such as X-ray crystallography, nuclear magnetic resonance or mutagenesis assays^2^.

Throughout the years, computational R2R-I prediction methods have been developed. However, their application is still limited since they often require additional data beyond sequence information. Methods based on computational docking^5,6^ require unbound structures or their template-based structures^7^. Methods based on co-evolution conjecture require homologous sequences of the given protein sequences^8,9^ to conduct multiple sequence alignment (MSA). Methods based on motifs^10,11^ rely on external motif databases. Methods^12,13^ based on interaction profiles of Hidden Markov Models (imHMMs)^14^ describing domain-domain interaction, have also been used to predict R2R-I in PPI. However, these interaction profiles need to be obtained from the external database 3DID^15^. To date, few R2R-I prediction methods use only sequence information. Those few^1,2^ still require trial-and-error combination of the features on external knowledge of the sequences, such as using external software to obtain features of the input sequences. Their key drawback is that a large amount of trial-and-error time is required for feature engineering, i.e. selecting the optimum feature combinations^16^ (e.g. surface accessibility, hydrophobicity, charge, etc.). The commonly used features for R2R-I prediction was recently reviewed^16^. One recent attempt^17^ using different R2R-I predictors for different types of PPI requiring prior knowledge of the type of PPI of the input protein sequences from users. Another attempt^18^ is to adopt deep learning using graph convolutional neuron networks, where advanced programming skills and high-end graphical processing units are required during the network development. Supplement Note 2 provides a detailed review of the existing work on computational R2R-I prediction methods.

This paper presents a new method, Pattern-to-Knowledge (P2K), which moves in a novel direction to predict R2R-I between two proteins based only on sequence information. It leverages the deep knowledge discovered from R2R-C data to acquire more specific features in building machine learning (ML) models not require time-consuming feature engineering of external sequence knowledge. By deep knowledge from a general perspective, we mean the physical incidences masked or entangled by subtle or unknown factors in the source environment, inconspicuous at the surface of the data. Nevertheless, this deep knowledge can be discovered from the data after a mathematical disentanglement process.

**Figure 1:**
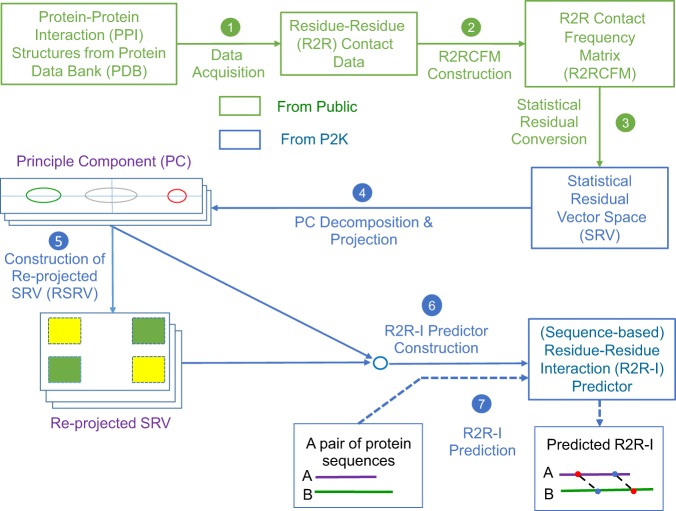
P2K Software System Overview. A Pattern to Knowledge (P2K) software system with its key components for discovering deep knowledge from Residue-Residue Contact (R2R-C) data in PPI, illustrated by its application to Dataset 618. The key procedures are marked by circled numerals. (1) Data Acquisition. PPI structures in the Protein Data Bank (PDB)^21^ were first procured, such as that of Dataset 618 consisting of 17,278 R2R-C pairs from 618 non-redundant 3D PPI complexes acquired in a previous study^20^. (2) R2R Contact Frequency Matrix (R2RCFM) Construction. R2RCFM was constructed from the frequency count of contact between residues obtained from the R2R contact data. Figure 3 provides an example of R2RCFM obtained from Dataset 618. (3) Statistical Residual Vector Space (SRV) Conversion. Each contact frequency in R2RCFM was converted into a statistical residual (SR), which accounts for the deviation of that frequency from the frequency if the contact is a random occurrence. For mathematical transformation, the matrix of SR is considered as an SR vector space (SRV) such that each row denotes a residue-vector (*r*-vector), with coordinates representing the SR of that residue interacting with other residues corresponding to the column *r*-vectors. Figure 4 provides an illustration of SRV. (4) Principal Component (PC) Decomposition and Projection. Applying PC Decomposition (PCD) on SRV, a set of PCs sorted by their corresponding eigenvalues in descending order was obtained. P2K projects the *r*-vectors onto different PCs, capturing different orthogonal SR associations governed by different specific R2R-I functionality (top panels of Figs 5(a–f)). (5) Construction of Re-projected SRV (RSRV). When the projections of the *r*-vectors on a PC are re-projected back to the SRV (referred to as RSRV) with a new set of coordinates in terms of the original basis vectors, they reflect the physiochemical R2R-Is captured in the PCs (bottom panels of Fig. 5(a–f)). (6) Construction of the R2R-I predictor. Leveraging the more precise and succinct statistical measures reflected by PC projections and RSRVs, a sequence-based R2R-I predictor was constructed (See Section 5 in Supplement Note 1 for details). **(7**) R2R-I Prediction. Given two protein sequences, Feature Vectors (FVs) were constructed for all residue pairs and inputted to the R2R-I predictor (See Section 5 in Supplement Note 1 for details).

**Figure 2:**
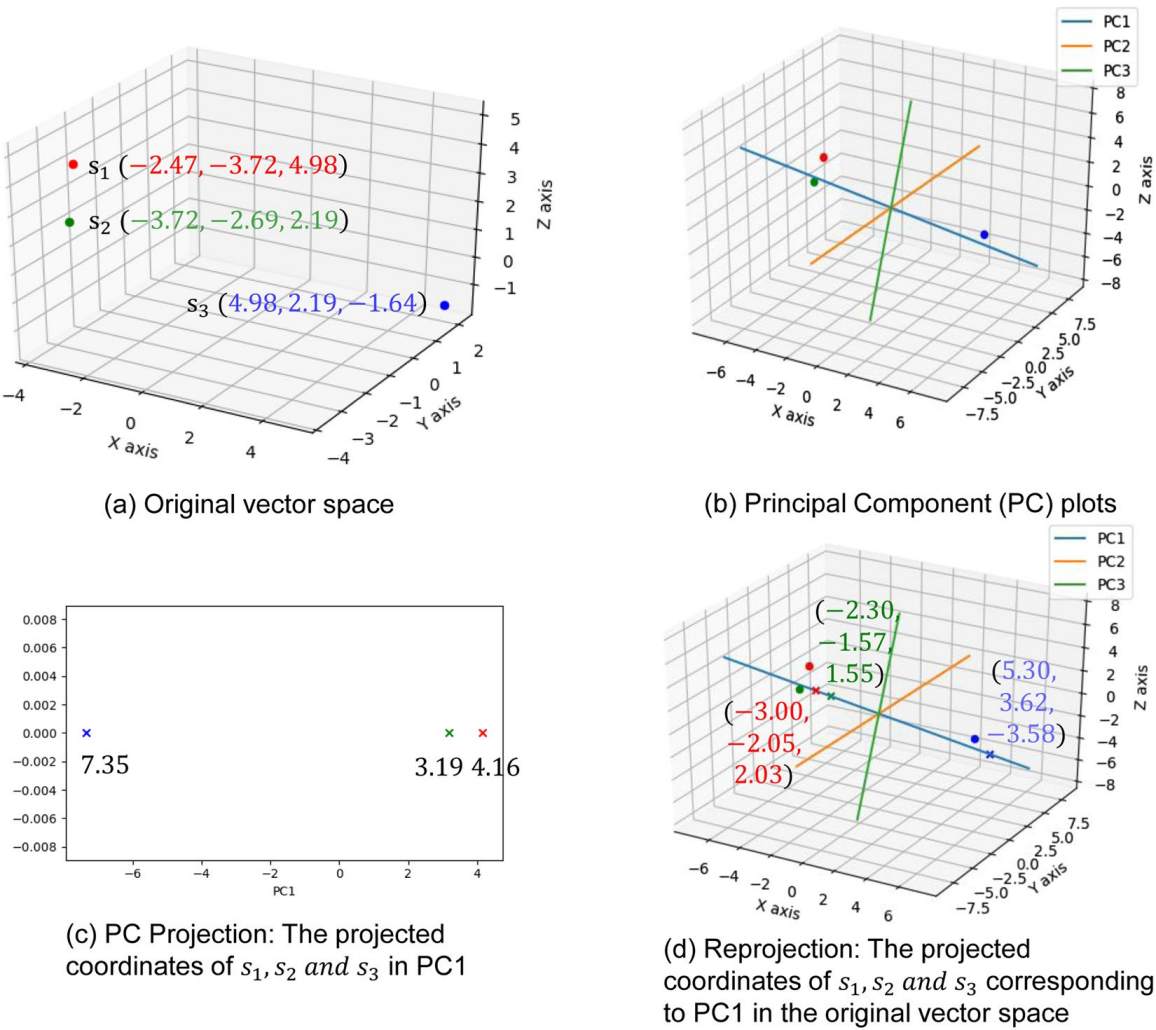
An illustration of PC projection and its re-projection. (**a**) Original vector space. Three points *s*_1_, *s*_2_ and *s*_3_ are plotted on the x-, y- and z-axes. Each point is represented as a circle and is colored red, green or blue, respectively. We denote the vector space spanned by the x-axis, y-axis and z-axis as the original vector space. The coordinates of *s*_1_, *s*_2_ and *s*_3_ are respectively (−2.47, −3.72, 4.98), (−3.72, −2.69, 2.19) and (4.98, 2.19, −1.64). A matrix *A* = [*s*_1_ *s*_2_ *s*_3_]^*T*^ with the dimension of 3 by 3 is constructed. **(b**) Principal Component (PC) plots. By applying Principal Component Decomposition (PCD)^22^ on matrix *A*, three principle components (PC1, PC2 and PC3) are obtained. These three PCs are plotted in the original vector space. **(c)** PC Projection: The projected coordinates of *s*_1_, *s*_2_ and *s*_3_ in PC1. We project *s*_1_, *s*_2_ and *s*_3_ on PC1. The projection of each vector is represented as a cross on the PC axis following the same coloring scheme as for *s*_1_, *s*_2_ and *s*_3_. The projected coordinates of *s*_1_, *s*_2_ and *s*_3_ in PC1 are respectively 4.16, 3.19 and −7.35. **(d)** Re-projection: The projected coordinates of *s*_1_, *s*_2_ and *s*_3_ corresponding to PC1 in the original vector space. We re-project the projected coordinates of *s*_1_, *s*_2_ and *s*_3_ in PC1 onto the original vector space. In other words, the projected coordinates of *s*_1_, *s*_2_ and *s*_3_ in PC1 are converted into the coordinates in the original vector space to reveal the association (relation) of *s*_2_, *s*_2_ and *s*_3_ captured in the PC. The reprojected coordinates of *s*_1_, *s*_2_ and *s*_3_ corresponding to PC1 are respectively (−3.00, −2.05, 2.03), (−2.30, −1.57, 1.55) and (5.30, 3.62, −3.58). They reveal a significant association of these points captured by the PC.

Figure 1 furnishes a schematic overview of P2K with procedural steps represented by numbered arrows and procedural outcomes listed inside each block. In steps 1, 2 and 3, we first transform the R2R-C Frequency Matrix into a Statistical Residual Space (SRV) to extract statistical deviation from randomness. Each row in SRV denotes a residue-vector (*r*-vector) with coordinates representing the SR (the statistical residual) of that residue interacting with other residues corresponding to the column *r*-vectors. In steps 4 and 5, we first apply Principal Component (PC) Decomposition (PCD) to the SRV to obtain PCs containing projections of *r*-vectors maximizing their variance. When these projections are re-projected back to the SRV (referred to as RSRV), their coordinates are the SRs of residue associations reflecting the physiochemical R2R-Is captured in the PCs. Since PCD is sensitive to scaling, the use of SR in the SRV unifies the scaling and enhances the statistical strength. We refer these PCs and RSRVs as the deep knowledge inherent in the complex R2R-I environments. Figure 2 provides an illustration of the coordinates of the *r*-vectors in the transformations. Empirically, we found the deep knowledge discovered could reveal the subtle yet specific physiochemical properties that a residue possesses.

To leverage the deep knowledge for ML, we developed a novel method known as P2K to acquire more R2R-I information for R2R-I prediction. Using the benchmark P2P docking benchmark dataset (DBD) version 4.0^19^, we conducted leave-one-complex-out-alone cross-validation to obtain much better prediction performance than an existing software platform that requires feature engineering over external knowledge of sequences.

This paper is structured as follows. Table 1 summarizes the terminology used in this paper. The Results section gives an overview of P2K with an illustrative application to Dataset 618^20^ followed by R2R-I prediction results. A case study is also conducted to illustrate the effectiveness of P2K. The Discussion section then summarizes the novelty and contributions of this paper. Supplement Note 1 provides a complete understanding of the P2K method; Note 2 a literature review on related work; Note 3 a detailed analysis of the experimental results over Dataset 618^20^ and Note 4 the details in the ML experiments. P2K is accessible via our web server on https://p2k.uwaterloo.ca.

**Table 1:** A legend of the terminology used in this study.

Term	Description
3D	Three-Dimensional
DBD	Protein-Protein Docking Benchmark Dataset
ML	Machine Learning
PC	Principal Component
PCD	Principal Component Decomposition
P2K	Pattern to Knowledge Software System
P2P	Protein-Protein
PDB	Protein Data Bank
PPI	Protein-Protein Interaction
*r*-vector	Residue vector, a row vector in SRV and a row in R2RSRM
R2R	Residue-Residue
R2R pair	A pair of residues
R2R-I	Residue-Residue Interaction
+ve R2R-I	A pair of residues, known to have R2R-I
−ve R2R-I	A pair of residues, known to not have R2R-I
Candidate R2R-I	A pair of residues, not yet known to have R2R-I or not
R2R-C	Residue-Residue Contact
R2R-C pair	A pair of residues with R2R-C
R2RCFM	Residue-Residue Contact Frequency Matrix
R2RSRM	R2R Statistic Residual Matrix (=SRV)
SR	Statistic Residual, which is computed by Adjusted Standard Residual
SRV	Statistic Residual Vector Space (=R2RSRM)
RSRV	Re-projected Statistic Residual Vector Space
Dataset 618	A dataset of 17,278 R2R-C pairs from 618 non-redundant 3D PPI complexes acquired in a previous study^20^

## Results

We first provide an overview of P2K with illustrative application to Dataset 618^20^ and furnish a detailed analysis of the R2R-I pattern disentanglement results. We then report our R2R-I prediction results by ML over the benchmark dataset P2P docking benchmark dataset (DBD) version 4.0^19^.

### P2K Discovery of Deep Knowledge, with Illustrative Results on Dataset 618

P2K is a software system capable of discovering deep knowledge from R2R-C data in PPI lab environment acquired from the PDB^21^. The deep knowledge discovered is then leveraged to construct a sequence-based R2R-I predictor. Figure 1 furnishes a schematic overview of P2K. For the definitions and a detailed description of the method, please refer to Supplement Note 1: Methods.

1. **Data Acquisition**. 618 non-redundant PPI structures used in a previous study^20^ were acquired from the PDB. We denote a pair of residues between proteins when the closest Euclidean distance between their C-Beta atoms (C-Alpha atoms for Gly (G))^19^ is less than 6Å^3,15,19^ in the 3D coordinate space as a R2R-C pair (Definition 1). From these 618 PPI structures, we acquired 17,278 R2R-C pairs. We denoted the dataset as Dataset 618 (Definition 2).
2. **R2R Contact Frequency Matrix (R2RCFM) Construction**. R2RCFM (Definition 3) was constructed from the R2R-C pairs collected from Dataset 618. From the R2RCFM (Fig. 3) we noticed that the frequency count of the C-C contact attributed to disulfide bonding is 49 close to 39 of the R-R contact while R-R (both positive) should not interact. This surprising finding led us to postulate that the underlying phenomena could be masked in the accounted data. It thus motivated us to develop the method to disentangle the statistics inherent in the R2R-C data.

**Figure 3:**
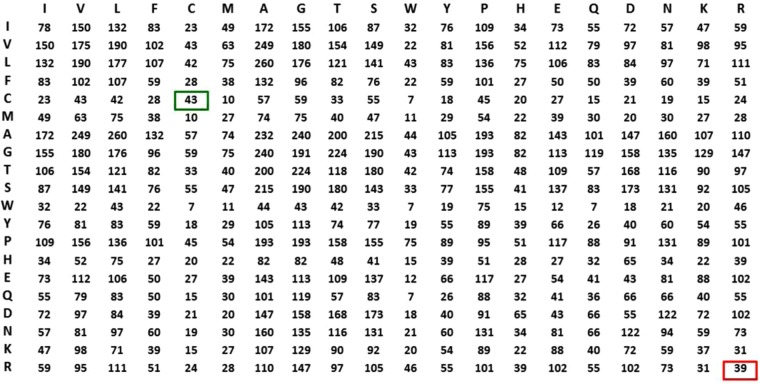
R2R Contact Frequency Matrix (R2RCFM). The figure was derived from Dataset 618, i.e. 618 non-redundant PPI structures with 17,278 R2R contact (R2R-C) pairs (<6 Å) in total. From this R2RCFM, note that the frequency count of the C-C contact, which potentially occurs through disulfide bonding, is 43, which is close to the number 39 observed for the R-R contact. This finding is surprising, due to repulsive electrostatic force between the positively charged residues. This observation indicates that using its absolute value for a valid prediction is not realistic or reliable.
3. **Statistical Residual Vector Space (SRV) Conversion**. R2RCFM is converted to a matrix by replacing each entry with a statistical measure, known as statistical residual (SR) (Definition 4), which accounts for the deviation of the observed frequency of the R2R-C pair from that if it is a random occurrence. The SR reveals the statistical significance of a R2R-C pair. At the confidence level of 95%, if the SR > 1.96, the discovered R2R-I is “+ve statistically significant”, colored yellow in the figures; “−ve statistically significant” if the SR <−1.96, colored green, and irrelevant or random if the SR is between −1.96 and 1.96. We refer this matrix as R2R Statistic Residual Matrix (R2RSRM) (Definition 5). For mathematical transformation, we treat R2RSRM as a vector space, called Statistic Residual Vector Space (SRV) (Definition 6), such that each row is considered as a residue-vector (*r*-vector) (Definition 7), with its coordinate representing the SR of that residue interacting with another residue corresponding to the column. Figure 4 shows the SRV obtained. However, there is still a problem. While the 15.35 value of SR between C-C (≫1.96) is reasonable, the SR value of 0.73 between R-R is still questionable since SR of R-R interaction should be below −1.96. Hence, we seek a method to reveal deeper knowledge embedded in the SRV.

**Figure 4:**
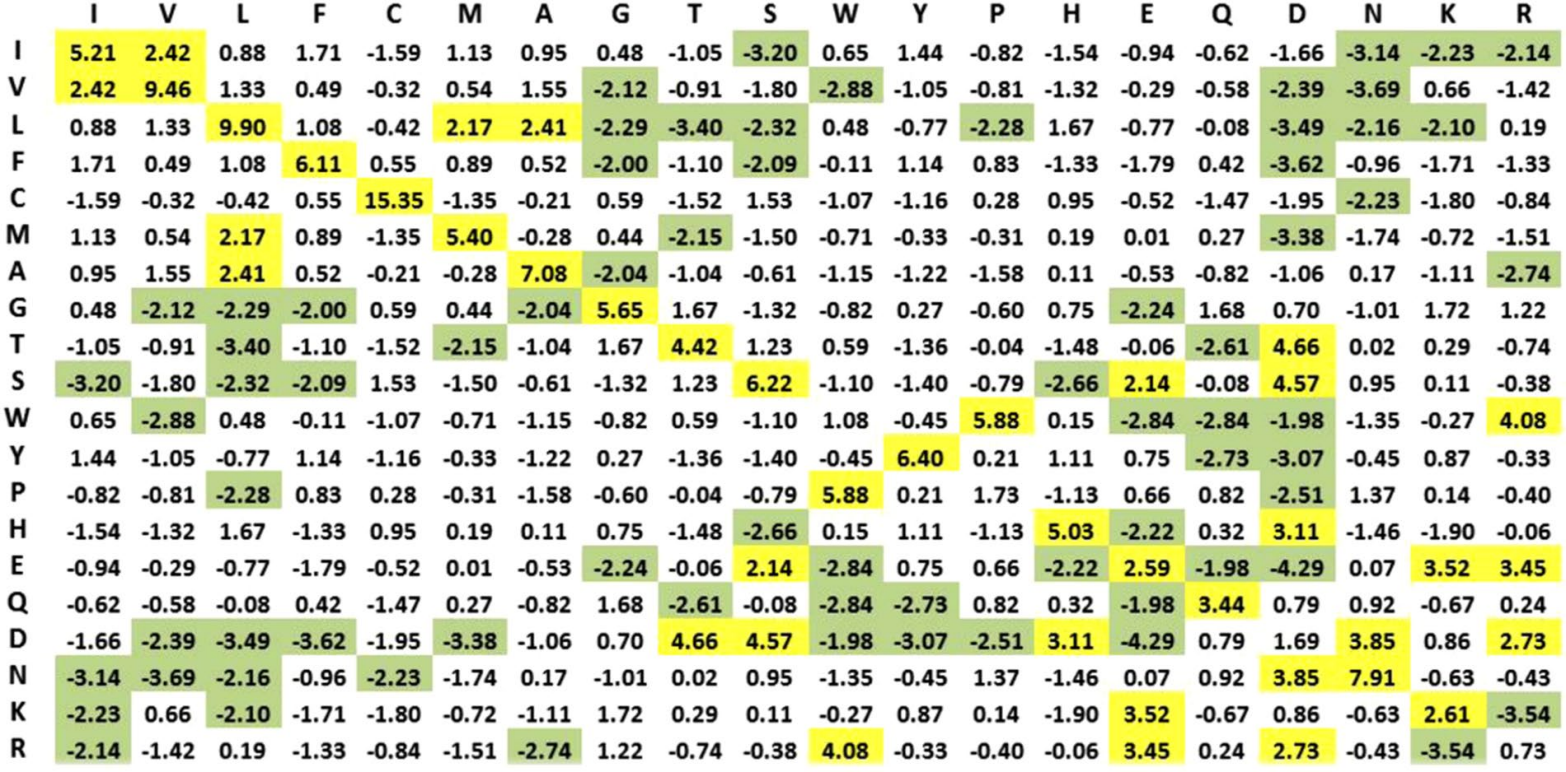
Statistical Residual Vector Space (SRV). The goal of converting R2RCFM to SRV is to extract statistical deviation from randomness. The figure was obtained from R2RCFM by converting each of its frequency counts into a Statistical Residual (SR). The SR measures the deviation of the observed frequency of occurrences of a R2R contact from the expected frequency if it is a random or irrelevant occurrence. For a confidence interval of 95%, the R2R contact is positively or negatively statistically significant if its SR is >1.96 (colored yellow) or its SR is <−1.96 (colored green), respectively. If the SR is between −1.96 and 1.96 exclusively, the R2R contact is statistically considered as a chance, random or irrelevant happening. The 15.35 value of SR between C-C (≫1.96) is reasonable; but that of 0.73 between R-R is questionable since R, a positively charged residue, is unlikely to interact with another positive residue R. Hence, we speculated that SRV might still be distorted by “noise” (e.g. external binding forces brought about by water molecules) and/or physiochemical entanglement (e.g. multiple types of physiochemical binding forces combining to bring two residues in close contact). Therefore, further measures are required to resolve this dilemma.
4. **Principal Component Decomposition (PCD) and** ***r*-Vector Projection**. We then apply PCD^22^ (Definition 8) on SRV to obtain orthogonal (uncorrelated) PCs^22^. A PC is a one-dimensional space (an axis) reflecting the variance of the projections of the *r*-vectors on it (as illustrated by the color crosses on the blue PC axis on Fig. 2(d). They are the projections of the *r*-vectors denoted by circles of the corresponding color). If the association of a group (say the green and red circles) is strong in certain subspace (i.e. coordinates), their projections on a certain PC should be far away from the mean, attributing to the large variance of that PC (like the crosses in Fig. 2(d)). Those distinct groups of *r*-vectors represent certain strong associations implying specific functionality in certain subspace captured by the PC. Note that when these color circle *r*-vectors are projected onto the PCs denoted by the orange and green axes, they would be located close to the mean with low variance, indicating that they are insignificant in these two PC spaces. This is the essence of “function disentanglement”. Based on the association of the *r*-vectors in SRV, PCD will obtain orthogonal PCs with their variance sorted in descending order. We selected the top 6 PCs with total data variance coverage up to 80% (Definition 9). We then projected (Definition 9) the *r*-vectors in the SRV onto each of these 6 PCs (Fig. 5(a–f), top). We observed that each PC reveals a type of R2R-I associated molecular property of the residue. For example, PC5 reveals whether a residue is charged. As shown at the top panel in Fig. 5(e)), the distinctive groups discovered are the green group (R) and the red group (W, E, D). Note that residue R is positively charged and residues E and D are negatively charged. While the residue W is not usually listed as having a negative charge, it was recently reported that its surface is negatively charged^23^. We also observed in PC5 that the green group (R) is projected at the left end, while the red group (W, E, D) is projected at the right end of the panel. This indicates that the projected coordinates reflect (by the correlated SR coordinates in RSRV5) the strength of the opposite molecular charge property.

**Figure 5:**
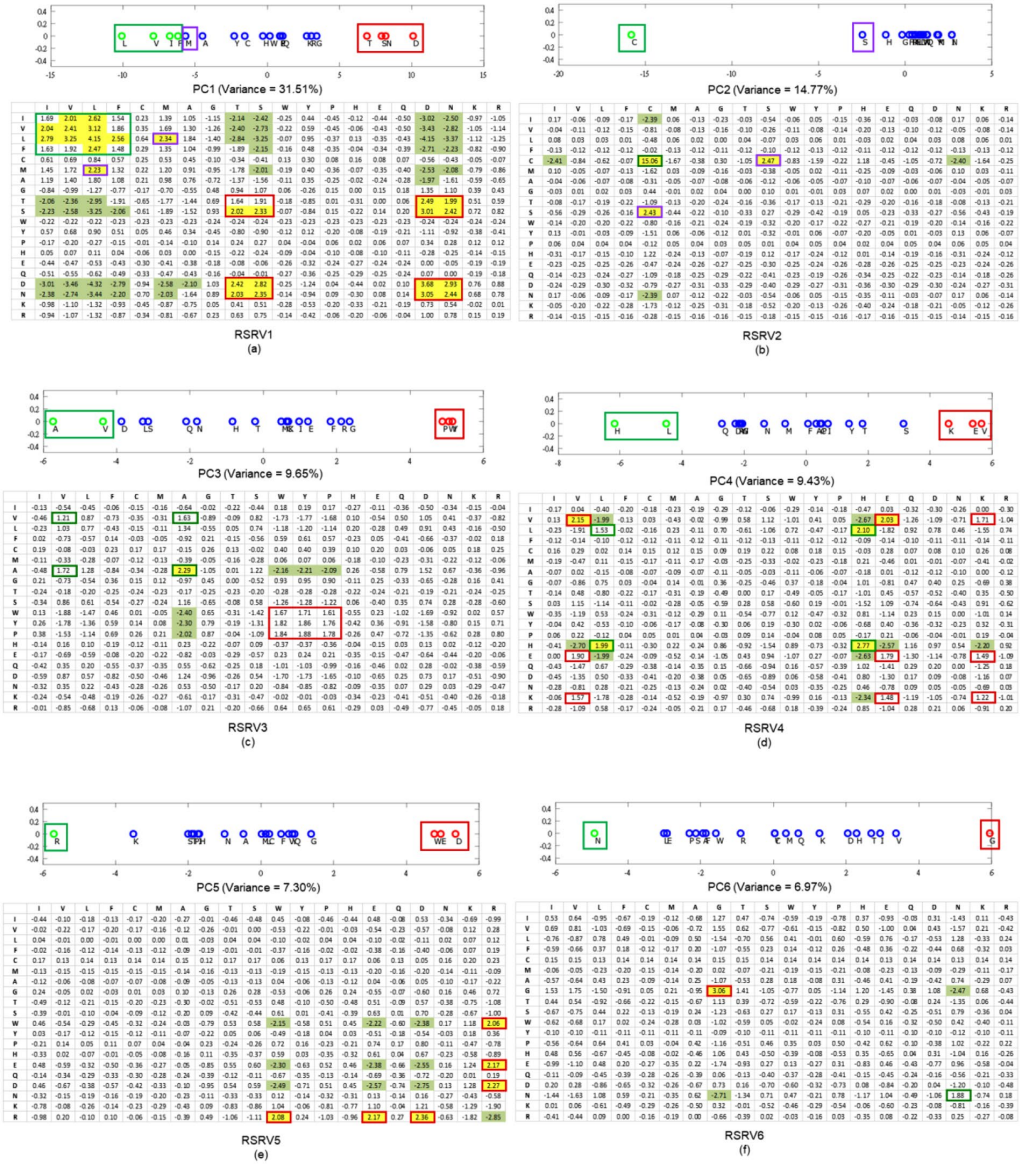
Projections of *r*-vectors from the SRV onto PCs and their re-projections onto RSRVs. The top panels in (**a**–**f**) show the projections of the *r*-vectors onto the top six PCs (PC1 to PC6), sorted by their corresponding eigenvalues. The distinct projection of an *r*-vector at the far left or far right (red and green boxes) of a PC indicates that certain of its coordinates (SRs) corresponding to the interacting residues are strong. The bottom panels in (**a**–**f**) show their SR coordinates when projected back to their RSRVs (RSRV1 to RSRV6 respectively). The projections of the *r*-vectors, functionally related, distinct and orthogonal to others in each PC and their corresponding SR coordinates re-projected on the RSRV, are enclosed in boxes of the same colored borders (C-Boxes). The corresponding analysis of these figures is found in our analysis below, and also in Supplement Note 3 with enlarged figures.
5. **Construction of Re-projected SRV (RSRV)**. To reveal the R2R-I strength captured in each PC projection, a re-projection (Definition 10) procedure was proposed. It maps the *r*-vector projections on a PC back to SRV, referred to as RSRV (Definition 10), with a new set of coordinates of the *r*-vectors reflecting their interaction captured by the PC. The color crosses on Fig. 2(d) show the projections of the *r*-vectors revealing their association strength captured in the PC in the explainable coordinates of RSRV corresponding to the same basis vectors of SRV. As shown in the bottom panels of Fig. 5(a–f), RSRV1 to RSRV6 were obtained by mapping the *r*-vector projections in PC1 to PC6 (the top panels in Fig. 5(a–f)) onto different RSRVs respectively. In Fig. 5(e), the matrix in the bottom panel is the RSRV5 of PC5. We observed that R-R is now negatively significant, while R-D, R-E and R-W are positively significant, consistent with our knowledge of charged residues. Thus, the R2R-I strength of these charged residues is distinguished in this RSRV space with statistical significance and functional relevance. These revealed SR strength can be considered as deep knowledge supporting R2R-I estimation and prediction.
6. **R2R-I Predictor Construction**. Leveraging the PC Projections and RSRVs, a sequence-based R2R-I Predictor was constructed (Section 5 in Supplement Note 1).
7. **R2R-I Prediction**. Given two protein sequences, P2K constructs feature vectors for all R2R–I candidates and inputs them to the R2R-I predictor for R2R-I prediction (Section 5 in Supplement Note 1).

### Detailed Analysis on Results Obtained from Dataset 618

In steps 4 and 5 of P2K (Fig. 1), we obtain and sort the PCs according to the variance and project the *r*-vectors on each separately. When the *r*-vector projection on a PC are re-projected onto the RSRV, their SR coordinates reflect the statistical strength of the R2R-I associating with particular physio-chemical properties captured by the PC.

The projections in PCs 1–6 are shown in Fig. 5(a–f) (top), with the corresponding eigenvalues covering a data variance of almost 80%. For the *r*-vector projections located over 1 standard deviation from the mean (colored green on the –ve side and red on the +ve side) they represent significant associations. The remaining ones colored blue signify insignificant associations in that particular PC. The corresponding RSRVs are shown in Fig. 5(a–f) (bottom). The yellow-shaded values signify SRs with positive statistical significance at a confidence interval of 95% (>1.96) while green-shaded values signify those with negative statistical significance at a confidence interval of 95% (<−1.96). Unshaded values represent statistically insignificant SRs. For each residue within a significant group in a PC, its projections onto the corresponding RSRV representing its statistical R2R-I preferences are highlighted by enclosing it in the C-box (box with colored borders) of the same color as the box in which it was enclosed in the PCs.

Guided by these statistical and functional findings, we conducted a literature search of protein hydrophobicity and other R2R-I properties reported from studies in other laboratories and established literature. Though most of the distinct residues in the top 6 PCs are found significant only in one PC and its corresponding RSRV, we noted some interesting exceptions, like PC3 and RSRV3. We also noticed that a few residues (like V, E and W) appear in more than one PC and RSRV. Both of these findings provide an indication of multiple interacting functionality of residues in the 3D environment. Here we list the major findings. Experimental and observational details are elaborated in Supplement Note 3.

1. **The projections of** ***r*-vectors in SRV on PC1** are shown in Fig. 5a (top), where the corresponding eigenvalue covers a data variance of 31.51%. We found that the green groups (L, V, I, F, M, A) are associated with a high hydrophobicity scale (or hydropathy index)^24^ and the red polar groups (T, S, N, D) with a low hydrophobicity scale. Table 2 summarizes their hydrophobicity scale to reflect the residues’ tendency to be found on the surface of a protein^25,26^. Hence, hydrophobicity plays an important role in intermolecular recognition processes^27^ such as PPI^28^, and has been used for predicting protein interface residues^29–32^. Comparing the projection coordinates of both groups in PC1 with their hydropathy index taken from the literature^24^ (Table 2; a full table is provided in Table S3.2 of Supplement Note 3), we were surprised to find that the statistical strength and the functional scale were highly correlated (the P-value of the Pearson correlation is 1.8E-05 (p < 0.05))–a perfect case to show that physical knowledge is reflected in a disentangled subspace from the R2R-C data (Supplement Note 3). Surprisingly, although we found that residue I has a strong R2R-I preference for hydrophobic V and L, it was reported as being generally non-reactive^33^ (Supplement Note 3). It is interesting to observe that I-L and V-L interactions, as reflected by their SRs, are relatively weak. The close correspondence of the P2K results and the physical knowledge furnishes convincing scientific evidence of P2K knowledge discovery capability since the entire experimental process had no input from the independently reported hydrophobicity scale^24^.

**Table 2:** The close correlation between the PC Projection of *r*-vectors on PC1 and the hydrophobicity scale of *r*-vectors.

Residues (*r*-vectors)	I	V	L	F	M	A	T	S	D	N
Hydrophobicity scale	4.5	4.2	3.8	2.8	1.9	1.8	−0.7	−0.8	−3.5	−3.5
PC1 Projection	−6.77	−7.89	−10.07	−6.21	−5.69	−4.47	6.91	7.91	10.08	8.20

The PC Projection values are results from the orthogonal R2R-I statistical/functional space obtained by disentangling R2R-C data. The well-established hydrophobicity scale values are taken from the literature^24^. We found that the green groups (L, V, I, F, M, A) are associated with a high hydrophobicity scale (or hydropathy index)^24^ and the red polar groups (T, S, N, D) with a low hydrophobicity scale.
  Further, in the RSRV1 (Fig. 5a, bottom), we observed R2R-I statistical preferences between members of the same group but not those of the other group. The positive statistically significant interactions among red group members (D, S, T, N) could be attributed to polarity or hydrophilic-hydrophilic interaction, while those among the green group (L, V, I, F, M, A) could be attributed to hydrophobic-hydrophobic interaction. Both are consistent with previous observations^20^. It has also been reported that surface patches with high hydrophobicity are energetically unfavorable in an aqueous solution, but favorable when in contact with other hydrophobic surfaces^34^. We noted that the SR of S-T is 1.23 (insignificant) in SRV, whereas in RSRV1 (Fig. 5a) the SR values of S-T and T-S are 2.02 (significant) and 1.91 (approaching significance), respectively. Disentanglement thus reveals that S-T does interact in a hydrophilic setting.
2. **The projections of** ***r*-vectors in SRV on PC2** are shown in Fig. 5b (top), where the corresponding eigenvalue covers a data variance of 14.8%. Note that the projection of C (in green) is highly distinctive in PC2. This is largely attributed to the formation of the C-C disulfide bond that plays a major role in PPI^35^, with high statistical significance as shown in RSRV2 (Fig. 5b, bottom). Though S (in the purple C-box) is not as distinct as C on PC1, it has significant interaction with C as seen in RSRV2, forming a C-S H-bond, which has been reported only in a recent study^36^. This information is masked in SRV (Fig. 5), as its SR assumes an insignificant (SR = 1.53) interaction, but was found to be significant (SR = 2.47) in RSRV2. This projection thus furnishes additional information useful to biologists and protein chemists to very rapidly identify and clarify R2R-Is between proteins of interest.
3. **The projections of** ***r*-vectors in SRV on PC3** are shown in Fig. 5c (top), where the corresponding eigenvalue covers a data variance of 9.76%. It reveals two discovered groups: the green group (A, V) and the red group (P, Y, W). We conjecture that they correspond to aliphatic-hydrophobic and aromatic groups respectively, where only residues within, but not between groups, could interact^37^. In RSRV3 (Fig. 5c, bottom)), most of the SRs in the C-boxes are slightly below 1.96, indicating weaker interactions, except for that between A-A, which is conjectured to be an aliphatic-aliphatic interaction^37^. We reasoned that the R2R-I statistical preferences between A and V could be attributed to aliphatic-hydrophobic interactions^37^, while those between Y and W to aromatic-aromatic interactions^37^. As for P with SR > 1.62, we found that P can weakly interact with aromatic residues. This might be due to a CH/π interaction^38^.
4. **The projections of** ***r*-vectors in SRV on PC4**, are shown in Fig. 5d (top), where the corresponding eigenvalue covers a data variance of 9.43%. This analysis reveals two significant groups: the green group (H, L) and the red group (K, E, V), where only members within, but not between groups interact. We speculated that interaction within the (K, E, V) group and the (H, L) group are due to hydrogen bonding (H-bonding). We found cases of both groups in 3D PPI complexes (Supplement Note 3). Our reasoning is that H-bonds are essential for determining binding specificity^39,40^ and provide favorable free energy for the binding^41^, whereas unfulfilled bonding resulting from the presence of a H-bonding residue without a bonding partner could destabilize binding^42^. The contrast in energetics contributes to a high selectivity in matching the H-bonds between proteins and confers binding specificity. Hence, we conjectured that these are H-bonds, and that they determine the binding specificity within these groups^39^.
5. **The projections of** ***r*-vectors in SRV on PC5**, are shown in Fig. 5e (top), where the corresponding eigenvalue covers a data variance of 7.30%. The distinctive green (R) and red (W, E, D) groups correspond respectively to positively and negatively charged residues. While the residue W is not usually listed as one with negative charge, a recent finding reported that its surface is negatively charged^23^, complying with the R2R-I knowledge (SR = 2.08) we discovered. Note that the projection of K is close to that of R, and K is found to be positively charged. This indicates again that the projected coordinates indeed reflect the molecular properties of these residues. From the yellow and green cells in RSRV5 (Fig. 5e, bottom), we noted that residues of opposite charges attract and those of similar charges repel. The R2R-Is for all other non-charged residues are insignificant and irrelevant in this charge dominating statistic space, the result of disentanglement.
6. **The projections of** ***r*-vectors in SRV on PC6** are shown in Fig. 5f (top), where the corresponding eigenvalue covers a data variance of 6.97%. The two distinctive groups are the green group (N) and the red group (G). In RSRV6 (Fig. 5f, bottom), G-G is statistically preferred and G-N is not. Though not significant, the SR of N-N is quite large (1.88). Note that it is reported that interaction involving G is not rationalized^20^. Additional research needs to be conducted to establish the molecular properties in this RSRV.

In most of these cases, we showed that R2R-I masked in R2RCFM and SRV are being disentangled and brought forth in their PCs and RSRVs, respectively. These findings render a strong statistical and functional base for P2K with well-established experimental laboratory support and validation. We should note that some of the findings predicted were independently reported only recently, suggesting that information inherent in R2R-C data can be uncovered by P2K.

### P2K Knowledge-Directed R2R-I Prediction using Protein-Protein Docking Benchmark Dataset 4.0

**Figure 6:**
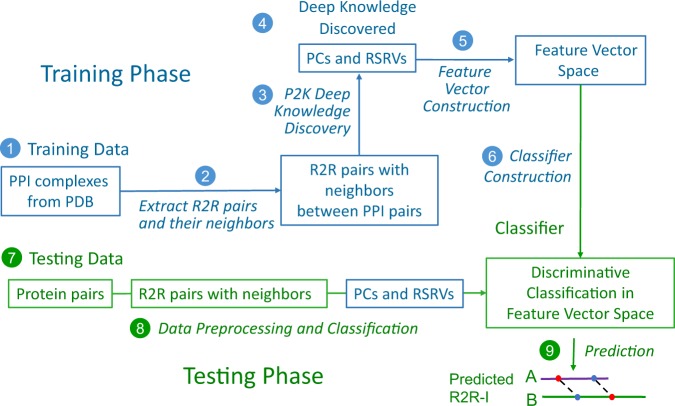
An Overview pf P2K Deep Knowledge Directed R2R-I Prediction. A new ML method using the deep knowledge discovered to direct the construction of a predictor for R2R-I prediction between interacting protein sequences. The key steps are marked in circled numerals. (1) Collection of Positive/Negative R2R-I in PPI Complexes from PDB for Experimental Testing. R2R pairs (a pair of residues residing on two different protein sequences) in PPI complexes were obtained from PDB. Previous studies^1,2,20^ suggested that if the contact distance between two residues is below a threshold (such as 6Å)^1,2,20^, R2R-I is likely to occur in the R2R pair. Hence, in our experiments, all R2R pairs, with contact distance $< 6\dot{A}$, were marked as having positive (+ve) R2R-I, otherwise ($\ge 6\dot{A}$) as having negative (-ve) R2R-I. See Definition 1 in Supplement Note 1 for details. (2) Acquisition of Deep Knowledge Discovered via P2K. We first obtained via P2K the deep knowledge, consisting of PCs and their corresponding RSRVs with top variance, on the + ve R2R-I. (3) Extraction of Neighbors of Positive/Negative R2R-I. For each positive/negative R2R-I taken in step 1, we exploited the R2R-I knowledge of their neighbors via FV construction in ML. Figure 7a gives an example of a R2R pair (A-V) with three neighbors on both sides (See Section 5 in Supplement 1 for details). (4) Construction of the FV for R2R-Is. For each positive/negative R2R-I, we applied the deep knowledge (the more precise disentangled statistical measures procured in PCs and RSRVs not only to the central R2R pair (A-V) but also to its six neighbors (three on each side) to build up its FV (an example is in Fig. 7a). Figure 7b depicts the transformation of the central R2R pair (A-V) with its neighboring residues into a 126-dimension FV. (5) Construction of the FV Space. We obtained the FV for R2R-I predictor construction from all positive/negative R2R-Is. (6) R2R-I Predictor Construction. From the FV Space obtained in step 5, we constructed a classifier through training, based on the training set with + ve and –ve R2R-I in the FV Space. (7) Procurement of Protein Pairs for Testing. Given two input protein sequences, we took all R2R pairs between them for R2R-I prediction. (8) Obtaining and Inputing the FVs for Prediction. Each R2R pair between the two proteins was transformed into an FV (like that in Fig. 7b) but without class labels. We inputted these FVs into the R2R-I predictor. (9) The R2R-I predictor then assigned each FV a score. We then outputted the R2R pairs with top scores as the predicted R2R-Is. An illustration is given in Fig. S1.8 in Supplement Note 1. See Section 5 in Supplement Note 1 for details.

Our next question is: “could the deep knowledge discovered be used to enhance R2R-I prediction?” In response, we developed a method known as Deep Knowledge-Directed ML (or P2K) for R2R-I Prediction. It uses the deep knowledge discovered to direct the construction of the predictor rather than relying on feature engineering. Figure 6 gives a description of this method through the steps marked in circled numerals.

### R2R-I Predictor Construction in the Training Phase

1. **Collection of Positive/Negative R2R-I in PPI Complexes from PDB for Prediction Experiments**. R2R pairs (a pair of residues residing on two different protein sequences) in PPI complexes were obtained from the PDB. In our experiments, we used the DBD version 4.0^19^. Previous studies^1,2,20^ suggested that if the contact distance between two residues is below a threshold (such as 6Å)^1,2,20^, R2R-I is likely to occur in the R2R pair. Hence, in our experiments, all R2R pairs, with contact distance $< 6\dot{A}$, were marked as having positive (+ve) R2R-I, otherwise they were considered to have negative (−ve) R2R-I. See Definition 1 in Supplement Note 1 for details.
2. **Acquisition of Deep Knowledge Discovered via P2K**. We first obtained via P2K the deep knowledge, consisting of PCs and their corresponding RSRVs with top variance, on the +ve R2R-I pairs. In this study, the top six PCs with a total variance of approximately 80% was selected to direct the predictor construction.
3. **Extraction of Neighbors of Positive/Negative R2R-I Pairs**. For each positive/negative R2R-I pairs chosen in step 1, we exploited the R2R-I knowledge of their neighbors via feature vector construction, a practice adopted in ML. Figure 7(a) gives an example of a R2R pair (A-V) with three neighbors on both sides.

**Figure 7:**
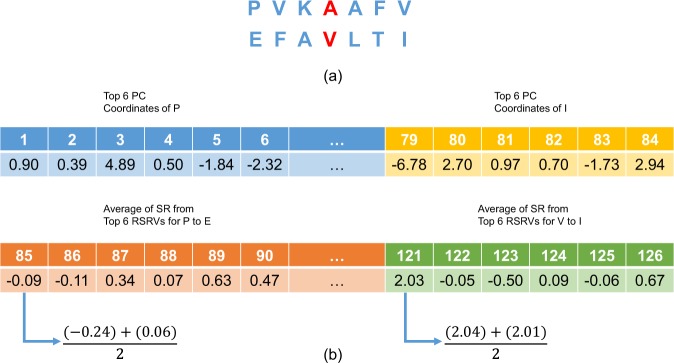
An Overview of P2K Deep Knowledge Directed R2R-I Prediction. (**a**) The central R2R pair with its neighbors. This figure gives an example of a R2R pair (A-V) with its neighbors **(b)** A 126-dimensional feature vector (FV) transformed from the R2R-pair with its neighbors shown in (**a**). Features 1 to 84 (6 × 14) would be in the projected values of the top 6 PCs of the 14 residue alphabets, while features 85 to 126 would be the average RSRVs of the top 6PCs obtained for each of the 7 R2R pairs (P-E, V-F, K-A, A-V, A-L, F-T and V-I) (6 × 7 = 42). Hence, the total number of features is 84 + 42 = 126.
4. **Construction of the Feature Vector (FV) for R2R-Is**. For each positive/negative R2R-I, we applied deep knowledge, which incorporates the more precise disentangled statistical measures procured in PCs and RSRVs not only to the central R2R pair (A-V) but also to its six neighbors (three on each side) to build up its FV (an example is presented in Fig. 7a). Figure 7b shows how the central R2R pair (A-V) with its neighboring residues were transformed into a 126-dimension FV. Features 1 to 84 would be the top 6 PC projection coordinates of the 14 residue alphabets (6 × 14 = 84), while features 85 to 126 would be the average RSRVs of the top 6 PCs obtained for each of the 7 R2R pairs (P-E, V-F, K-A, A-V, A-L, F-T and V-I) (6 × 7 = 42). Hence, the total number of features is 84 + 42 = 126, which is the dimension of the FV.
5. **Construction of the Feature Vector Space**. From all positive/negative R2R-Is, we obtained their FV for R2R-I predictor construction subsequently.
6. **R2R-I Predictor Construction**. From the FV Space obtained in step 5, we constructed an extra decision tree classifier^43^ (one variant of the widely used classifier random forests) with 1000 trees through training using the ML package scikit-learn 0.18.2^44^ under default parameters based on the training set with + ve and −ve R2R-I in the FV Space.

### R2R-I Predictor Operation in the Testing phase

The R2R-I predictor constructed could be used for predicting R2R-I between two input protein sequences. This Testing Phase is described in the continuing steps

(7) **Protein Pairs for Testing**. Given two input protein sequences, we took all R2R pairs between them for R2R-I prediction.
(8) **Obtain and Input the FVs for Prediction**. Each R2R pair between the two proteins was transformed into an FV (like that in Fig. 7b), but without class labels. We inputted these FVs into the R2R-I predictor.
(9) **The R2R-I predictor then assigned each FV a score**. We then outputted the R2R pairs with top scores as the predicted R2R-Is. An illustration is given in Fig. S1.8 in Supplement Note 1. See Section 5 in Supplement Note 1 for details.

In this experiment we used the DBD 4.0^19^, which contains 176 non-redundant PPI complexes. We also subdivided the data into training and test sets. The training set consisted of the first 124 non-redundant PPI complexes accessible from PDB^21^ (Supplement Table 4.2). The data set is equivalent to the dataset version 3.0 (DBD 3.0)^45^. The test set included the last 52 non-redundant PPI complexes in DBD 4.0^19^ (Supplement Table 4.2).

In ML, to evaluate a binary classifier, the area under a receiver operating characteristic (ROC) curve^46^, known as AUC (Area Under Curve), is often used. The higher the AUC value, the better the prediction performance. Its maximum and minimum value is 1.0 and 0.0 respectively. A random predictor would obtain a value of 0.5.

We first validated the R2R-I prediction results of P2K over the first 124 PPI complexes in DBD 4.0^19^ under leave-one-complex-out-alone cross-validation (See Supplement Note 4 for details). As shown in Supplement Table 4.3, P2K has achieved a higher average AUC (0.69078 ± 0.02757) than that of a Random Predictor (0.50000 ± 0.00000). Even when no neighboring residue is considered, the average AUC (0.59600 ± 0.01531) is still much higher than that of a Random Predictor. This strongly indicates that the deep knowledge discovered from R2R-C data is effective for R2R-I prediction.

We also took absolute values of the PC projections and re-ran the experiments. We found that the prediction performance (as shown in Supplement Table 4.4) in our testing set was not substantially altered. There is not enough statistically significant evidence to indicate that taking absolute values of the PC projections would result in better or worse prediction performance. Hence, from a ML perspective, we would adopt the original values of the PC projections in the construction of FV processes. (See Supplement Note 4).

We then conducted a comparative study with an existing sequence-based R2R-I predictor, PPiPP^1^, through its assessable web interface. Like other ML approaches, PPiPP^1^ also requires leveraging feature engineering over external knowledge of sequences whereas P2K does not. In this paper, we compared our experimental results with those of PPiPP^1^ which is the closest counterpart available. P2K (1) requires only sequence input; (2) does not need prior information over the type of PPI of the input protein sequences provided by the users; (3) does not require hand-end graphical processing units in neither constructing nor using the ML models; and (4) provides an easy-to-use web interface. PPiPP^1^ is the only available existing software fulfilling these four criteria. Supplement Note 2 provides a literature review on related work.

The comparative results between PPiPP^1^ and P2K in Table 3 showed that P2K achieved a higher average AUC (0.64317 ± 0.04159) than that of PPiPP^1^ (0.50112 ± 0.00257), 22% better with statistical significance (two-tailed paired student’s t-test p-value: 1.9E-08 < 0.05). We also observed that the prediction performance generally increased with the number of neighboring residues incorporated in the FVs (See Supplement Tables 4.5 and 4.6). This suggests that the R2R-I environments have intriguing features, and the use of deep knowledge is definitely beneficial.

**Table 3:** A summary of the average AUC achieved by P2K compared with PPiPP^1^ for the 52 PPI complexes newly introduced in DBD 4.0^19^.

Method	Average AUC
PPiPP^1^	0.50112 ± 0.00257
P2K (no. of neighboring residues considered = 0; no. of features = 18)	0.55911 ± 0.02502
P2K (no. of neighboring residues considered = 1; no. of features = 54)	0.59947 ± 0.03251
P2K (no. of neighboring residues considered = 2; no. of features = 90)	0.61933 ± 0.04069
P2K (no. of neighboring residues considered = 3; no. of features = 126)	**0.64317** ± **0.04159**

### Case Study

**Figure 8:**
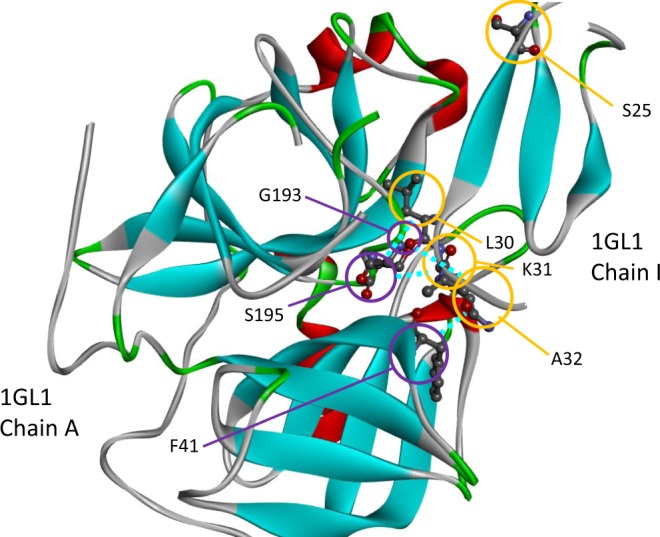
A demonstration of the 3D configuration of the R2R-I prediction between protein sequence chains A and I on the target complex 1GL1. The residues on protein chain A are enclosed by purple circles while the residues on the protein sequence chain I are enclosed by yellow circles. The 4 out of 5 true positive predictions of P2K are shown in the figure and are connected by light blue dash lines. They are 1: (S195 on chain A, K31 on chain I), 2: (S195 on chain A, L30 on chain I), 3: (F41 on chain A, A32 on chain I) and 4: (G193 on chain A, A32 on chain I). The false positive prediction is S195 on chain A with S25 on chain I that are located at a distance from each other. We do not link them by a blue dash line. As shown in Supplement Table 4.7, P2K outperformed PPiPP^1^, where P2K had a precision of 80% among the top 5 predictions while PPiPP^1^ had a precision of 0% among the top 5 predictions.

To demonstrate the effectiveness of P2K for illustration purpose, we conducted a case study on the two protein sequence chains of the target complex 1GL1-A:I, which is one of the 52 PPI complexes newly introduced in protein-protein docking benchmark dataset version 4.0 (abbreviated as DBD 4.0^19^). This target complex has the PDB^21^ ID 1GL1, which describes the PPI between bovine alpha-chymotrypsin and PMP-C, an inhibitor from the insect Locusta migratoria. According to DBD 4.0^19^, R2R-I occurs on protein sequence chains A and I. Following the procedure mentioned, we trained P2K over the first 124 PPI complexes in DBD 4.0^19^ under default parameter setting except setting the number of neighboring residues to be 3. Also, the latest scikit-learn machine learning package 0.19.2^44^ was adopted. We then applied P2K to predict R2R-I between the chains A and I of the target complex 1GLI. A working practice of an experimental biologist is to focus on the top predictions. Hence, in this experiment, only the top 5 positive predictions and the other positive predictions were forced to be considered as negative predictions. As shown in Table 4, P2K outperformed PPiPP^1^, where P2K had a precision of 80% among the top 5 predictions while PPiPP^1^ had a precision of 0% among the top 5 predictions. Figure 8 provides a 3D illustration of the prediction in a 3D configuration. The input and output of the case study can be referred to Section 6 in Supplement Note 1.

**Table 4:** Precision, Recall, Specificity, F-Measure achieved by P2K comparing with PPiPP^1^ on the target complex 1GL1-A:I on DBD 4.0^19^, where in each case only the top 5 positive predictions remained and the other positive predictions were forced to be negative predictions^50,51^.

	TP	FP	TN	FN	Precision	Recall	Specificity	F1
P2K	4	1	8104	17	0.80000	0.19048	0.99988	0.30769
PPiPP^1^	0	5	8100	21	0.00000	0.00000	0.99938	0.00000

## Discussion

In this study, P2K succeeded in integrating three components important in analyzing and predicting R2R-I between two protein sequences. It uses: (a) more succinct and precise statistical measures to analyze data; (b) disentangled statistical measures to unveil and extract more specific deep knowledge; and (c) deep knowledge discovered to construct FVs for building R2R-I predictors. Hence, P2K opens a new chapter in sequence-based R2R-I prediction using sequence data only and does not require external knowledge of the sequences. It produced significantly superior performance than a random predictor, as well as an existing software platform that relies on feature engineering on external knowledge of sequences. It renders a promising new path to further enhance R2R-I prediction. This is the first time using the explainable deep knowledge discovered from data to enhance ML. P2K used a selected set of disentangled statistical vector spaces rather than conducting time-consuming feature engineering as in current ML. Since the deep knowledge discovered is explainable, it represents a pioneering work in deep knowledge discovery and explainable AI.

The key insight we gained in this study is that the disentangled statistical measures in SRV onto PCs and RSRVs could unveil deep knowledge inherent in the three-dimensional R2R-I environments, including knowledge previously known to the biology community and some only recently reported. There were also some findings that require additional research to produce a clearer R2R-I picture.

R2R-I prediction in P2K could be further enhanced when more specific R2R-I locations of conserved local functional regions are obtained through Aligned Pattern Clusters (APCs)^47–49^ obtained by our WeMine software^47–49^. Furthermore, ensemble classifiers could be constructed to combine different neighboring residue settings to further improve the predictive power. P2K could also be applied for partner-specific R2R-I prediction in protein-antibody binding. This would have great potential in drug discovery, once the false positives are significantly lowered. We also seek to advance P2K as a scientific method with strong statistical support and functional relevance to assist biological research, particularly when specific patterns rapidly discovered by P2K can be directly validated experimentally in laboratories, thus greatly improving the efficiency of identifying novel R2R-I. We are confident that P2K will open a new scientific frontier in extracting deep disentangled, quantifiable and verifiable knowledge from data in other biomedical domains.

### Availability of data, materials and methods

The PPI structure data is publicly available, where the data accessible method is described in the data availability statements. All computer software and codes to generate results that are reported in the paper and central to its main claims are available upon request.

## Electronic supplementary material

Supplementary Information

## Data Availability

The PPI structure data, i.e. Dataset 618, that supports the findings of this study are available in the previous study^20^ with the identifier(s) [doi:10.1002/1097-0134(20010501)43:2<89::AID-PROT1021>3.0.CO;2-H]. The PPI structure data, DBD version 4.0^19^, that support the findings of this study are available in [ZDock: Protein Docking], [https://zlab.umassmed.edu/zdock/].
