# Supplementary material for: Pattern to Knowledge: Deep Knowledge-Directed Machine Learning for Residue-Residue Interaction Prediction
Source: Sci Rep. 2018 Oct 4;8:14841. doi: 10.1038/s41598-018-32834-z (PMC6172270; doi:10.1038/s41598-018-32834-z)
Supplement: Supplementary file 1 — Supplementary Information [file 41598_2018_32834_MOESM1_ESM.pdf]

Pattern to Knowledge: Deep Knowledge-Directed Machine Learning for Residue-Residue Interaction Prediction

Andrew K.C. Wong[1,*], Ho Yin Sze-To[1] and Gary L. Johanning[2]

1: Department of Systems Design Engineering, University of Waterloo, Ontario, Canada N2L 3G1

2: SRI International, Biosciences Division, 333 Ravenswood Ave, Menlo Park, CA, USA

*Correspondence to akcwong@uwaterloo.ca

Supplement Note 1: Methods

While the main text furnishes a brief description of the proposed method, this document provides a comprehensive explanation of the definitions and the rationale of the proposed method. For ease of understanding and completeness, we restate certain parts as presented in the main text.

The structure of Supplement Note 1 is described as below.

Section 1.1 – "Introduction" provides the background and an overview of the proposed method.

Section 1.2 – "P2K Overview with Illustrative Results on Dataset 618" explains the proposed method in detail, with examples of the results applying on Dataset 618.

Section 1.3 – "Definitions of P2K and Details of the Illustrative Experiment on Dataset 618" provides a comprehensive understanding of the definitions, with examples of the results applying on Dataset 618.

Section 1.4 – "Formal Derivation of the Statistical Residual of the Observed Frequency of a Residue Pair" provides a formal mathematical derivation of the statistical residual.

Section 1.5 – "R2R-I Predictor Construction and Operation" provides a detailed description on how the R2R-I predictor is constructed and how it operates.

Section 1.6 – "Illustration of the input and Output R2R-I Predictor" provides a description on the input and output of the R2R-I predictor.

Section 1.7 – "References in Supplement Note 1" provides the references used in this document.

1.1. Introduction

In Protein-Protein interaction (PPI), residue-residue interaction (R2R-I) prediction refers to the identification of pairs of interacting residues, usually under close contact, residing on separate interacting proteins. Identification of R2R-I is critical as it enhances our understanding of PPI and furnishes potential targets for inhibiting the PPI[1]. One example of the utility of R2R-I is the use of small molecules to inhibit the interaction between p53 and MDM2[2], a potential cancer treatment. However, despite its importance, the identification of R2R-I in PPI is hampered by expensive, labor-intensive and time-consuming experiments, such as X-ray crystallography, nuclear magnetic resonance or mutagenesis assays[3].

Throughout the years, computational R2R-I prediction methods have been developed. However, their application is still limited as these methods often require additional data beyond sequence information. Methods[4,5] based on computational docking need to have unbound structures or their template-based structures[6]. Method based on co-evolution conjecture require homologous sequences of the given protein sequences[7,8] to conduct multiple sequence alignment (MSA). Methods[9,10] based on motifs rely on external motif databases. Methods[11,12] based on interaction profiles of Hidden Markov Models (imHMMs)[13], which describe domain-domain interaction, have also been used to predict R2R-I in PPI, where these imHMMs are obtained from an external database termed 3DID[14].

On the other hand, there are a few methods[3,15] requiring only sequence information. However, existing methods based on sequence information[3,15] rely on feature engineering over external knowledge of the sequences, such as using external software to obtain features of the input sequences. The key drawback is that a large amount of time is needed in the trial-and-error process of engineering features, i.e. selecting the optimum combinations of features[16] (e.g. surface accessibility, hydrophobicity, charge…). There is a recent review[16] on the commonly used features for R2R-I prediction. One recent attempt[17] is to use different R2R-I predictors for different types of PPI but it requires the users to have prior knowledge over the type of PPI of the input protein sequences. Another recent attempt[18] is to adopt deep learning using graph convolutional neuron network, where advanced programming skills and high-end graphical processing units are required during its development.

Our method, Pattern-to-Knowledge (P2K), moves in a new direction to predict R2R-I between two proteins based only on sequence information, leveraging the deep knowledge discovered from R2R contact (R2R-C) data and uses the discovered deep knowledge to acquire features for building machine learning models. The entire process requires neither time-consuming feature engineering over external knowledge on the sequences nor high-end hardware equipment during the construction of the machine learning models.

1.2. P2K Overview with Illustrative Results on Dataset 618

P2K is a software system which could discover deep knowledge from R2R-C data in PPI structures acquired from Protein Data Bank (PDB)[19], and leverage the deep knowledge discovered to construct a sequence-based R2R-I predictor. What exactly is deep knowledge? From a general prospective, deep knowledge means the physical occurrences which are masked or entangled by subtle or unknown factors. In other words, the subtle and not obvious knowledge could not be obtained from a superficial inspection of the data. It means that certain happenings observed from the data could be misleading because they are masked or overwhelmed by happenings caused by some other unknown or multiple entwining factors. For example, two residues with positive charge should not interact but their close contacts are frequently accounted from instances as recorded in the surface value of the R2R-C data (Fig. S1.2). This implies that quite frequent, their close contacts accounted in the 3-D space could be caused by other nearby stereo structural or interacting factors. However, the fact that residues of opposite charge attract and same charge repel still govern R2R-I and should be embedded somewhere in the data. That statistics, if disentangled, could be unveiled, extracted and used for predicting R2R-I. Can such deeper level of knowledge not obvious in the observed data be discovered and disentangled from the statistics inherent in the given data? This motivates the development of P2K. Fig. S1.1 furnishes a schematic overview of P2K, with procedures represented by arrows and procedure outcomes presented in blocks. The definitions are written in Section 3 in Supplement Note 1. A detailed exposition of the major steps is given below corresponding to the labelled steps in Fig. S1.1.

(1) Data Acquisition. R2R-C data in PPI structures are acquired from Protein Data Bank (PDB) [19]. 618 non-redundant PPI structures used in a previous study[20] were acquired from PDB. Previous studies[3,15,20] suggested that two residues are considered as a R2R contact pair (R2R-C) (Definition 1) if their contact

distance between them is below a certain threshold (such as 6Å[3,15,20]). From these 618 PPI structures, we acquired 17,278 R2R-C pairs. We denoted the dataset as Dataset 618 (Definition 2).

(2) **R2R Contact Frequency Matrix (R2RCFM) Construction.** R2RCFM (Definition 3) is constructed from the R2R-C pairs collected. After the construction of a R2RCFM over the 17,278 R2R-C pairs in Dataset 618, we found the R2RCFM (Fig. S1.2) that the frequency count of the C-C contact attracted via disulfide bond was 43, close to the value of 39 for R-R contact repelled by positive electrostatic force. This surprising finding led us to postulate that statistical conversion of observed data was needed to reveal deeper knowledge which should still be inherent in the data. Fig. S1.2. is the R2RCFM obtained from Dataset 618.

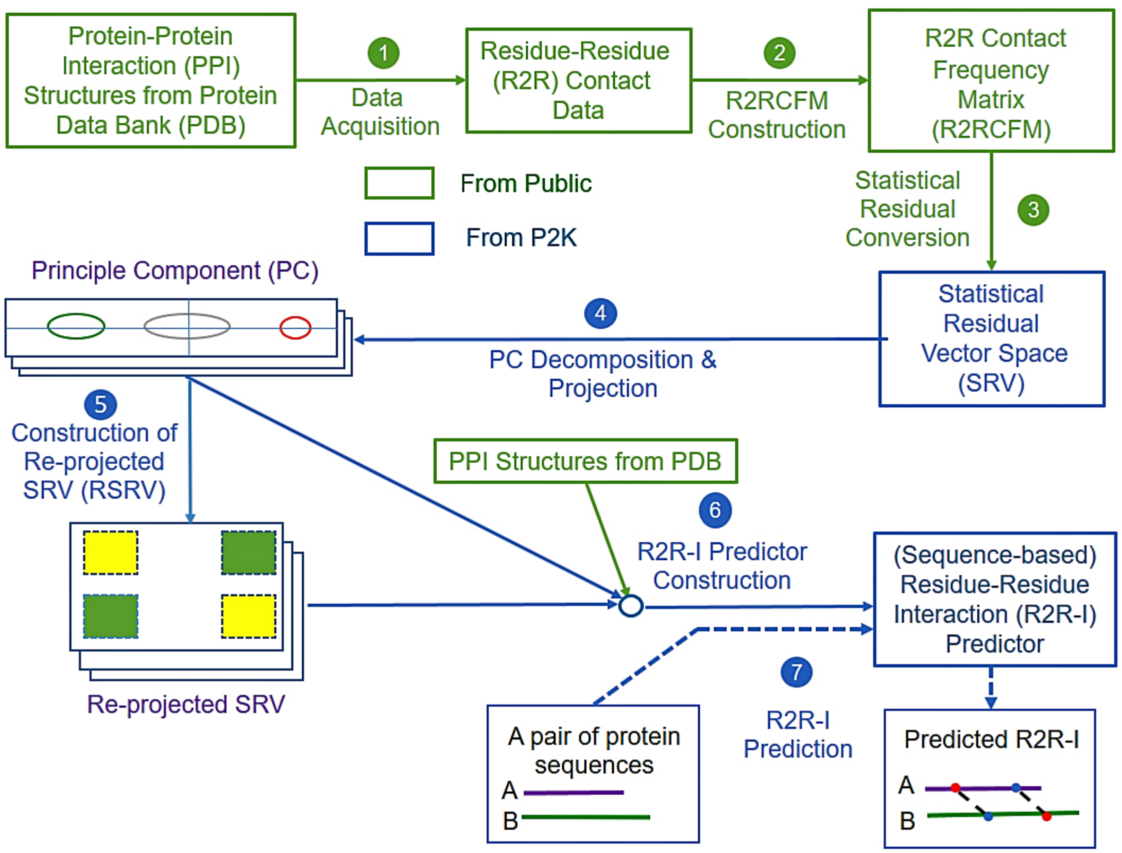

Figure S1.1. P2K Software System Overview with illustrative results on Dataset 618 A Pattern to Knowledge (P2K) software system with its key components for discovering deep knowledge from Residue-Residue Contact (R2R-C) data in the Protein-Protein Interaction (PPI). The key procedures are marked by circled numerals. **(1) Data Acquisition**. R2R-C data in PPI structures are first acquired from in Protein Data Bank (PDB)[19]. 17,278 R2R-C pairs were acquired from 618 non-redundant 3D PPI complexes collected in a previous study[20] and this dataset was designated as Dataset 618. (**2) R2R Contact Frequency Matrix (R2RCFM) Construction.** R2RCFM is constructed from the frequency count of contact between residues obtained from the R2R-C data. Fig. S1.2 provides an example of R2RCFM obtained from Dataset 618. **(3) Statistical Residual Vector Space (SRV) Conversion**. Each contact frequency in R2RCFM is converted into a statistical residual (SR) accounting for the deviation of that frequency from that if the contact is a random happening. We denote this matrix after conversion as R2R Statistic Residual Matrix (R2RSFM). For mathematical transformation, R2RSRM is also considered as an SR vector space (SRV) such that each row denotes a residue-vector (r-vector) with coordinates representing the SR of that residue interacting with other

residues corresponding to the column r-vectors. Fig. S1.3 provides an illustration of SRV. **(4) Principal Component (PC) Decomposition and Projection**. Applying Principal Component Decomposition (PCD), a set of PCs sorted by their corresponding eigenvalues in descending order is obtained. After projecting *r*-vector s on the PCs, we found that the statistical interacting strength among *r*-vector s associating with different types of molecular interaction properties was reflected by the distance of the projection position of the *r*-vector s from the mean of the PC (top panels of Fig. S1.4 (a) to (f)). **(5) Construction of Re-projected SRV (RSRV)**. To reveal and analyze the statistical information captured by the PC projections, an *r*-vector re-projection procedure is carried out to map the r-vector PC projections back to SRV, referred to as Re-projected SRV (RSRV) (bottom panels of Fig. S1.4 (a) to (f)). **(6) Construction of the R2R-I Predictor**. Leveraging the projected coordinates in PCs and the reprojected coordinates in RSRVs, a sequence-based R2R-I predictor can be constructed. (See Section 5 in Supplement Note 1 for details). The predictor takes the values from PCs and RSRVs to construct feature vectors (FVs) for R2R-I prediction. **(7) R2R-I Prediction.** Given two protein sequences, P2K constructs FVs for all residue pairs and input them to the R2R-I Predictor to predict R2R-I. (See Section 5 in Supplement Note 1 for details).

(3) **Statistical Residual Vector Space (SRV) Conversion**. To extract the deviation from randomness, the R2RCFM is converted to a matrix by replacing each matrix entry with a statistical measure, known as statistical residual (SR) (Definition 4), which accounts for the deviation of the observed frequency of the R2R-C pair if it is a random happening. We refer this matrix as R2R Statistic Residual Matrix (R2RSRM) (Definition 5). For later mathematical transformation to reveal deep knowledge, we treat R2RSRM as a vector space, called Statistic Residual Vector Space (SRV) (Definition 6), such that each row is considered as a residue-vector (*r*-vector) (Definition 7), in which each vector coordinate represents the SR of that residue interacting with other residue corresponding to the column. Fig. S1.3 provides an illustration of SRV. Nevertheless, at this stage, we still observe a problem in the SRV. While the 15.35 value of SR (>> 1.96) between C-C (disulfide bond) is reasonable, the SR value of 0.73 between R-R is questionable because R is positively charged and its SR should be below -1.96.

(4) **Principal Component Decomposition (PCD) and Projection**. PC Decomposition (PCD)[21] (Definition 7) was conducted on SRV to obtain PCs[21] sorted by their eigenvalues in descending order. It is a statistical procedure to convert a set of observations of possibly correlated variables into a set of values of linearly uncorrelated variables called Principal Components (PC). We use the PCD to sort out *correlated r-vectors with strong association and later the RSRV to reveal the SR of their R2R-I* associations captured by the PC with other residues. We selected the top 6 PCs so that the total data variance coverage was almost 80% (Definition 7). We then conducted PC Projection (Definition 8) of the *r*-vectors in SRV onto these 6 PCs, shown at the top panels in Fig. S1.4 (a)-(f). We observed that each PC reveals a type of molecular interaction property of residues. For example, PC5 reveals if a residue is charged. As shown on the top panel in Fig. S1.4 (e)), the distinctive groups discovered are the green group (R) and the red group (W, E, D). Note that residue R is positively charged and residues E and D are negatively charged. While the residue W is not usually listed as one with negative charge, a recent finding[22] reported that its surface is negatively charged. We also observed that the green group (R) is projected at the left end, while the red group (W, E, D) is projected at the right end. It indicates that the projected coordinates reflect (by the correlated SR coordinates in the SRV) the statistical interacting strength of the molecular interaction property, i.e. if the charged residue is positively or negatively charged.

(5) **Construction of Re-projected SRV (RSRV)**. On the PC, we observe that the projection of a *r*-vector, if located far away from the mean (zero), signifies its strong SR associating with other residues, but we do not know exactly which one(s) and the corresponding SR values. For example, a distinct group of residues like (W, E, D) with projections of their *r*-vectors on PC5 close to each other in Fig. S1.4 (e) suggests that their correlated R2R-I are strong, but we do not know the SR strength of the R2R-I pairs. To find that out, a re-projection procedure (Definition 10) is introduced. It maps the *r*-vector projections on the PC back to the SRV with new SR coordinates reflecting the R2R-I captured by the PC. We refer

this SRV as the Re-projected SRV (RSRV) (Definition 10). As shown at the bottom panels of Fig. S1.4 (a)-(f), RSRV1 to RSRV6 were obtained by mapping the *r*-vector projections in PC1 to PC6 (the top panels in Fig. S1.4 (a)-(f)) onto SRV, respectively. In Fig. S1.4 (e), the matrix at the bottom panel is the RSRV5 of PC5. We observed that R-R is –ve significant, while R-D, R-E and R-W are +ve significant, consistent with our knowledge that residues of the same charge repel while those of opposite charge attract. While we observe that W, E, D are away from the mean and close to each other on PC5, we notice that the SR values of their interactions are succinctly revealed in RSRV5. In Fig. S1.4 (a) to (f), we observe that the statistical strength of specific R2R-I of various residue groups is reflected in the PC projections as well as the SR values in the RSRV. Hence both PCs and RSRVs bring out the statistical significance and functional relevancy of R2R-I.

(6) **R2R-I Predictor Construction**. Leveraging the projected coordinates in PCs and the reprojected coordinates in RSRVs, a sequence-based R2R-I predictor can be constructed). The predictor takes the deep knowledge discovered in the PCs and RSRVs to construct feature vectors (FVs) through ML for R2R-I prediction (See Section 5 for details).

(7) **R2R-I Prediction.** Given two protein sequences, P2K constructs FVs for all residue pairs and input them to the R2R-I Predictor to predict R2R-I (See Section 5 for details).

1.3. Definitions of P2K and Details of the Illustrative Experiment on Dataset 618

Definition 1 – Residue-Residue Contact (R2R-C). R2R-C refers to an event where two residues are considered to be in close contact in the 3D coordinate space when the closest Euclidean distance between their C-Beta atoms (C-Alpha atoms for Gly (G))[20]) is less than 6Å[3,15,20]. Though other distance-based definitions have been used, the results are consistent with those with the 6Å distance[23]. A R2R-C pair is referred to as a pair of residues (r_i,r_j) in close contact in the 3D coordinate space, where $r_i, r_j \in \Sigma_\mathrm{R}$.

Definition 2 – Dataset 618. Refers to a R2R-C dataset obtained from a previous study[20] recording the PDB ID of 618 non-redundant PPI structures from which 17,278 R2R-C pairs were acquired. Note that the previous study[20] recorded the PDB ID of 621 PPI structures, but we removed 3 of them. They are 1reg-CD (Reason: 1reg has no C, D chains), 1seb-EH (Reason: Redundancy) and 4tsl-AB (Reason: No such complex).

Definition 3 – R2R Contact Frequency Matrix (R2RCFM). R2RCFM is a $|\Sigma_\mathrm{R}| \times |\Sigma_\mathrm{R}|$ matrix where each entry $(\mathrm{R2RCFM})_{i,j}$ records the contact frequency of a pair of residues (r_i,r_j). Since all residue pairs are undirected, (r_i,r_j) and (r_j,r_i) are equivalent and $(\mathrm{R2RCFM})_{i,j} = (\mathrm{R2RCFM})_{j,i}$. Fig. S1.2 is the R2RCFM obtained from Dataset 618.

	I	V	L	F	C	M	A	G	T	S	W	Y	P	H	E	Q	D	N	K	R
I	78	150	132	83	23	49	172	155	106	87	32	76	109	34	73	55	72	57	47	59
V	150	175	190	102	43	63	249	180	154	149	22	81	156	52	112	79	97	81	98	95
L	132	190	177	107	42	75	260	176	121	141	43	83	136	75	106	83	84	97	71	111
F	83	102	107	59	28	38	132	96	82	76	22	59	101	27	50	50	39	60	39	51
C	23	43	42	28	43	10	57	59	33	55	7	18	45	20	27	15	21	19	15	24
M	49	63	75	38	10	27	74	75	40	47	11	29	54	22	39	30	20	30	27	28
A	172	249	260	132	57	74	232	240	200	215	44	105	193	82	143	101	147	160	107	110
G	155	180	176	96	59	75	240	191	224	190	43	113	193	82	113	119	158	135	129	147
T	106	154	121	82	33	40	200	224	118	180	42	74	158	48	109	57	168	116	90	97
S	87	149	141	76	55	47	215	190	180	143	33	77	155	41	137	83	173	131	92	105
W	32	22	43	22	7	11	44	43	42	33	7	19	75	15	12	7	18	21	20	46
Y	76	81	83	59	18	29	105	113	74	77	19	55	89	39	66	26	40	60	54	55
P	109	156	136	101	45	54	193	193	158	155	75	89	95	51	117	88	91	131	89	101
H	34	52	75	27	20	22	82	82	48	41	15	39	51	28	27	32	65	34	22	39
E	73	112	106	50	27	39	143	113	109	137	12	66	117	27	54	41	43	81	88	102
Q	55	79	83	50	15	30	101	119	57	83	7	26	88	32	41	36	66	66	40	55
D	72	97	84	39	21	20	147	158	168	173	18	40	91	65	43	66	55	122	72	102
N	57	81	97	60	19	30	160	135	116	131	21	60	131	34	81	66	122	94	59	73
K	47	98	71	39	15	27	107	129	90	92	20	54	89	22	88	40	72	59	37	31
R	59	95	111	51	24	28	110	147	97	105	46	55	101	39	102	55	102	73	31	39

Figure S1.2. R2R Contact Frequency Matrix (R2RCFM). The figure was derived from Dataset 618, i.e. 618 non-redundant Protein-Protein Interaction (PPI) structures with 17,278 R2R contact (R2R-C) pairs (< 6Å) in total. From this R2RCFM, note that the frequency count of the C-C contact, which potentially attracts through a disulfide bond, is 43. This number is close to the value of 39 observed for the R-R contact, which potentially repels through positively charged electrostatic force. This observation indicates that frequency count needs to be converted into a statistical measure for fair comparison.

Definition 4 – Statistical residual (SR). The statistical residual s_{r_i,r_j} of a residue pair (r_i,r_j) is defined as the deviation of the observed frequency of (r_i,r_j) in R2R contact from the default case where the happening is random. Assuming the occurrence of contact follows a normal distribution, for a confidence interval of

95%, the observed frequency of a residue pair (r_i,r_j) in R2R contact is considered as positively or negatively statistically significant if its SR is >1.96 or < -1.96 respectively, while those with SR between -1.96 and 1.96 are considered chance, random or irrelevant happening. s_{r_i,r_j} is defined as

$$s_{r_i,r_j} = \frac{X_{r_i,r_j} - N \cdot \mathrm{p}(\mathrm{r}_i, \mathrm{r}_j)}{\sqrt{N \cdot \mathrm{p}(\mathrm{r}_i, \mathrm{r}_j) \cdot \left(1 - \mathrm{p}(\mathrm{r}_i, \mathrm{r}_j)\right)}}$$

where X_{r_i,r_j} is the observed number of a residue pair (r_i, r_j) in R2R contact; N is the total observed number of residue pairs in R2R contact and $\mathrm{p}(\mathrm{r}_i, \mathrm{r}_j)$ is the probability of observing (r_i, r_j) in R2R contact if it is a random happening. The formal derivation of s_{r_i,r_j} is provided in Section 4.

Definition 5 – R2R Statistical Residual Matrix (R2RSRM). R2RSRM is a $|\Sigma_\mathrm{R}| \times |\Sigma_\mathrm{R}|$ matrix derived from $(\mathrm{R2RCFM})_{i,j}$ such that

$$(\mathrm{R2RSRM})_{i,j} = s_{r_i,r_j}$$

	I	V	L	F	C	M	A	G	T	S	W	Y	P	H	E	Q	D	N	K	R
I	5.21	2.42	0.88	1.71	-1.59	1.13	0.95	0.48	-1.05	-3.20	0.65	1.44	-0.82	-1.54	-0.94	-0.62	-1.66	-3.14	-2.23	-2.14
V	2.42	9.46	1.33	0.49	-0.32	0.54	1.55	-2.12	-0.91	-1.80	-2.88	-1.05	-0.81	-1.32	-0.29	-0.58	-2.39	-3.69	0.66	-1.42
L	0.88	1.33	9.90	1.08	-0.42	2.17	2.41	-2.29	-3.40	-2.32	0.48	-0.77	-2.28	1.67	-0.77	-0.08	-3.49	-2.16	-2.10	0.19
F	1.71	0.49	1.08	6.11	0.55	0.89	0.52	-2.00	-1.10	-2.09	-0.11	1.14	0.83	-1.33	-1.79	0.42	-3.62	-0.96	-1.71	-1.33
C	-1.59	-0.32	-0.42	0.55	15.35	-1.35	-0.21	0.59	-1.52	1.53	-1.07	-1.16	0.28	0.95	-0.52	-1.47	-1.95	-2.23	-1.80	-0.84
M	1.13	0.54	2.17	0.89	-1.35	5.40	-0.28	0.44	-2.15	-1.50	-0.71	-0.33	-0.31	0.19	0.01	0.27	-3.38	-1.74	-0.72	-1.51
A	0.95	1.55	2.41	0.52	-0.21	-0.28	7.08	-2.04	-1.04	-0.61	-1.15	-1.22	-1.58	0.11	-0.53	-0.82	-1.06	0.17	-1.11	-2.74
G	0.48	-2.12	-2.29	-2.00	0.59	0.44	-2.04	5.65	1.67	-1.32	-0.82	0.27	-0.60	0.75	-2.24	1.68	0.70	-1.01	1.72	1.22
T	-1.05	-0.91	-3.40	-1.10	-1.52	-2.15	-1.04	1.67	4.42	1.23	0.59	-1.36	-0.04	-1.48	-0.06	-2.61	4.66	0.02	0.29	-0.74
S	-3.20	-1.80	-2.32	-2.09	1.53	-1.50	-0.61	-1.32	1.23	6.22	-1.10	-1.40	-0.79	-2.66	2.14	-0.08	4.57	0.95	0.11	-0.38
W	0.65	-2.88	0.48	-0.11	-1.07	-0.71	-1.15	-0.82	0.59	-1.10	1.08	-0.45	5.88	0.15	-2.84	-2.84	-1.98	-1.35	-0.27	4.08
Y	1.44	-1.05	-0.77	1.14	-1.16	-0.33	-1.22	0.27	-1.36	-1.40	-0.45	6.40	0.21	1.11	0.75	-2.73	-3.07	-0.45	0.87	-0.33
P	-0.82	-0.81	-2.28	0.83	0.28	-0.31	-1.58	-0.60	-0.04	-0.79	5.88	0.21	1.73	-1.13	0.66	0.82	-2.51	1.37	0.14	-0.40
H	-1.54	-1.32	1.67	-1.33	0.95	0.19	0.11	0.75	-1.48	-2.66	0.15	1.11	-1.13	5.03	-2.22	0.32	3.11	-1.46	-1.90	-0.06
E	-0.94	-0.29	-0.77	-1.79	-0.52	0.01	-0.53	-2.24	-0.06	2.14	-2.84	0.75	0.66	-2.22	2.59	-1.98	-4.29	0.07	3.52	3.45
Q	-0.62	-0.58	-0.08	0.42	-1.47	0.27	-0.82	1.68	-2.61	-0.08	-2.84	-2.73	0.82	0.32	-1.98	3.44	0.79	0.92	-0.67	0.24
D	-1.66	-2.39	-3.49	-3.62	-1.95	-3.38	-1.06	0.70	4.66	4.57	-1.98	-3.07	-2.51	3.11	-4.29	0.79	1.69	3.85	0.86	2.73
N	-3.14	-3.69	-2.16	-0.96	-2.23	-1.74	0.17	-1.01	0.02	0.95	-1.35	-0.45	1.37	-1.46	0.07	0.92	3.85	7.91	-0.63	-0.43
K	-2.23	0.66	-2.10	-1.71	-1.80	-0.72	-1.11	1.72	0.29	0.11	-0.27	0.87	0.14	-1.90	3.52	-0.67	0.86	-0.63	2.61	-3.54
R	-2.14	-1.42	0.19	-1.33	-0.84	-1.51	-2.74	1.22	-0.74	-0.38	4.08	-0.33	-0.40	-0.06	3.45	0.24	2.73	-0.43	-3.54	0.73

Figure S1.3. Statistical Residual Vector Space (SRV). The figure was obtained from R2RCFM in Figure S1.2. by converting each of its frequency counts into a Statistical Residual (SR). SR is >1.96 is colored yellow and SR is < -1.96 is colored green. The 15.35 value of SR between C-C (>> 1.96) is reasonable; but the SR value of 0.73 between R-R is questionable since R, a positively charged residue, is unlikely to interact with another positive residue R. Thus, we would expect that its SR should be less than -1.96. Here we speculated that there are additional factors influencing this SRV such as multiple types of physiochemical binding forces added up together to bring two residues in close contact, and external binding forces brought by water molecules and/or physiochemical entanglement. This motivated the use of Principal Component Decomposition to disentangle the underlying factors.

Definition 6 – Statistical Residual Vector Space (SRV): R2RSRM can be considered as a vector space referred to as Statistical Residual Vector Space (SRV). Fig. S1.3 is the SRV obtained from Dataset 618. Formally,

$$(\mathrm{SRV})_{i,j} = (\mathrm{R2RSRM})_{i,j}$$

Definition 7 – Residue vector (*r*-vector): A residue vector (*r*-vector) for the residue *i* is the ith row vector in the SRV such that its jth coordinate is the SR of the residue i interacting with the residue *j*. Mathematically, it is denoted as

$$\boldsymbol{r}_i = (\mathrm{SRV})_{i,:}$$

Definition 8 – Principal Component Decomposition (PCD): Given a matrix A with n rows and p columns, PCD[21] decomposes the matrix into at most p eigenvectors (referred to as PCs) sorted by their eigenvalues in descending order. When applying on SRV, PCD[21] obtains at most $|\Sigma_\mathrm{R}|$ PCs. Formally:

$$PCD(\mathrm{SRV}) = \{(v_k, \tau_k)|1 \le k \le |\Sigma_\mathrm{R}|\}$$

where v_k is the ktheigenvector represented as a column vector with $|\Sigma_\mathrm{R}|$ rows, and τ_k is its corresponding eigenvalue. The eigenvectors are sorted such that $\tau_{k+1} \ge \tau_k$. In practice, we determine an appropriate value of k such that the data variance coverage is almost 0.8.

$$varaince\ coverage = \frac{1}{\sum_{b=1}^{|\Sigma_\mathrm{R}|} \tau_b} \sum_{h=1}^{\mathrm{k}} \tau_h \approx 0.80$$

Definition 9 – PC Projection. Given SRV and its kth PC v_k, we can obtain a column vector p_k with $|\Sigma_\mathrm{R}|$ real values[21] , where each real value represents a projected coordinate (corresponding to the kth PC of SRV) from a *r*-vector from SRV. Formally, we define a PC projection as:

$$project(\mathrm{SRV}, v_k) = p_k$$

where p_k is also denoted as the kth PC projection containing the projections of all the *r*-vector s in SRV. Top panels of Fig. S1.4 (a) to (f)) are the PC projection of SRV obtained from Dataset 618.

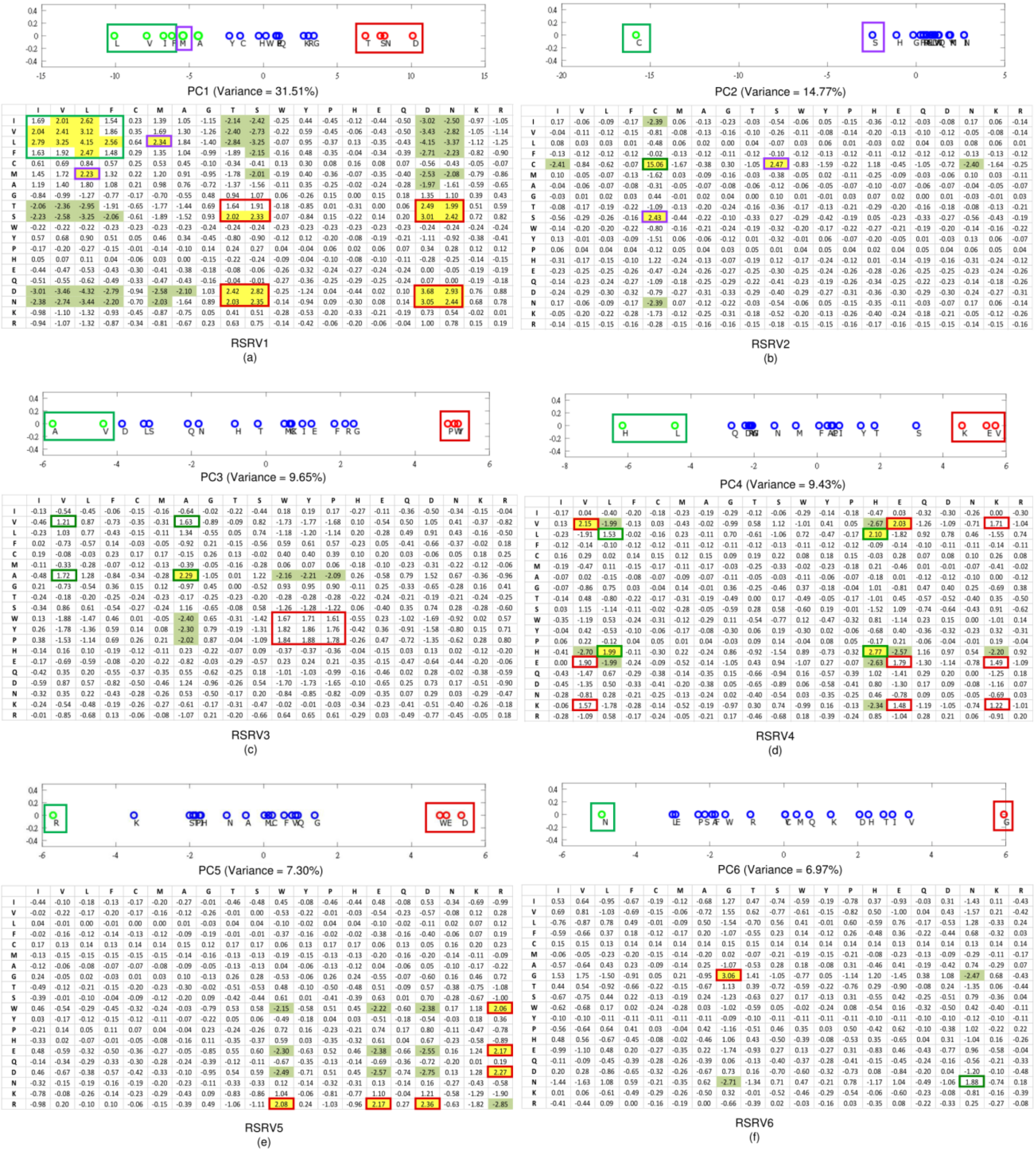

Figure S1.4. Projections of *r*-vector s from the SRV onto PCs and their re-projection onto RSRVs. The top panels in **(a)-(f) s**how the projections of the *r*-vector s onto the top six PCs (PC1 to PC6) sorted by their corresponding eigenvalues. The distinct projection of an *r*-vector at the far left or far right (red and green boxes) of a PC indicates that certain of its coordinates (SRs) corresponding to the interacting residues are strong. The bottom panels in (**a)-(f)** show the SR coordinates when projected back to their RSRVs (RSRV1 to RSRV6 respectively). The projections of the *r*-vector s functionally related, distinct and orthogonal to others in each PC and their corresponding SR coordinates re-projected on the RSRV are enclosed in boxes of the same colored borders (C-Boxes). The corresponding analysis of these figures is found in our analysis below and in Supplement Note 3.

Definition 10 – Re-projection of PC projection. Given a SRV, the kth PC v_k of SRV with the kth PC Projection p_k of SRV, the re-projection of p_k is defined as

$$Reproject(\text{SRV}, p_k, v_k) = p_k \cdot {v_k}^T + C^{SRV}$$

Here, C^{SRV} is defined as a matrix with at a dimension of $|\Sigma_\text{R}| \times |\Sigma_\text{R}|$:

$$C^{SRV} = \begin{bmatrix} c_{1,1} & c_{1,2} & \cdots & c_{1,|\Sigma_\text{R}|} \\ c_{2,1} & c_{2,2} & \cdots & c_{2,|\Sigma_\text{R}|} \\ \cdots & \cdots & \cdots & \cdots \\ c_{|\Sigma_\text{R}|,1} & c_{|\Sigma_\text{R}|,2} & \cdots & c_{|\Sigma_\text{R}|,|\Sigma_\text{R}|} \end{bmatrix}$$

where each matrix entry $c_{1,j} = c_{2,j} = \cdots = c_{|\Sigma_\text{R}|,\text{j}}$ is set as the mean of the j-th column of SRV, for $j = 1,2, \dots, |\Sigma_\text{R}|$.

We also denote the matrix, at a dimension of $|\Sigma_\text{R}| \times |\Sigma_\text{R}|$, obtained by the Re-projection of the kth PC projection as the kth re-projected SRV (RSRVk):

$$\text{RSRV}k = Reproject(\text{SRV}, p_k, v_k)$$

Bottom panels of Fig. S1.4 (a) to (f)) are the Re-projection of PC projection of SRV obtained from Dataset 618.

Definition 11 – Disentanglement of SRV (Table S1.1). Given SRV, the disentanglement of SRV is to obtain a set of {RSRV1, RSRV2,…RSRVk}. Formally it is defined as:

$$Disentangle(\text{SRV}) = \{\text{RSRV}k | 1 \le k \le |\Sigma_\text{R}|\}$$

Table S1.1. Disentanglement of SRV

**Disentanglement of SRV**
Input: SRV
Output: $\{\mathbf{RSRV}\boldsymbol{k} | \mathbf{1} \le \boldsymbol{k} \le |\boldsymbol{\Sigma}_\mathbf{R}|\}$
Step 1: Apply PCD on SRV to obtain $\{(\boldsymbol{v_k}, \boldsymbol{\tau_k}) | \mathbf{1} \le \boldsymbol{k} \le |\boldsymbol{\Sigma}_\mathbf{R}|\}$ (See Definition 8)
Step 2: Select k (See Definition 8)
Step 3: Project SRV on $\boldsymbol{v_1}, \boldsymbol{v_2}, \dots \boldsymbol{v_k}$ to obtain $\boldsymbol{p_1}, \boldsymbol{p_2}, \dots, \boldsymbol{p_k}$ (See Definition 9)
Step 4: Re-project $\boldsymbol{p_1}, \boldsymbol{p_2}, \dots, \boldsymbol{p_k}$ to obtain **RSRV1**, **RSRV2**,…, **RSRV$\boldsymbol{k}$** (See Definition 10)

1.4. Formal derivation of the statistical residual of the observed frequency of a residue pair

As stated in Definition 4, SR, s_{r_i,r_j} is a statistical residual that accounts for the deviation of the observed frequency of a residue pair (r_i,r_j) in R2R contact if it is a random happening. Technically, it is in the form of adjusted standard residual[24]. For a confidence interval of 95%, the observed frequency of a residue pair (r_i,r_j) in R2R contact is positively or negatively statistically significant if its SR is >1.96 or < -1.96 respectively. If the SR is between -1.96 and 1.96 exclusively, the observed frequency of a residue pair (r_i,r_j) in R2R contact is statistically considered as a chance, random or irrelevant happening. s_{r_i,r_j} is defined as

$$s_{r_i,r_j} = \frac{X_{r_i,r_j} - N \cdot \mathrm{p}(\mathrm{r}_i, \mathrm{r}_j)}{\sqrt{N \cdot \mathrm{p}(\mathrm{r}_i, \mathrm{r}_j) \cdot \left(1 - \mathrm{p}(\mathrm{r}_i, \mathrm{r}_j)\right)}}$$

Here we provide a formal derivation of s_{r_i,r_j} leveraging (adjusted) standard residual[24]. For easier presentation, we refer r_i as the ith element in the residue alphabet set $\sum_R$. Thus we first define the observed frequency of a residue pair in R2R contact involving r_i as:

$$\mathrm{f}(r_i) = \sum_{j=1}^{|\Sigma_R|} (\mathrm{R2RCFM})_{i,j}$$

We can further define the probability of observing a residue pair in R2R contact involving r_i as:

$$\mathrm{p}(\mathrm{r}_i) = \frac{\mathrm{f}(\mathrm{r}_i)}{\sum_{j=1}^{|\Sigma_R|} \mathrm{f}(\mathrm{r}_j)}$$

Next, if the occurrence of a residue pair in R2R contact is random, we define the probability of observing a residue pair $(\mathrm{r}_i, \mathrm{r}_j)$ in R2R contact as:

$$\mathrm{p}(\mathrm{r}_i, \mathrm{r}_j) = \begin{cases} \mathrm{p}(\mathrm{r}_i) \cdot \mathrm{p}(\mathrm{r}_i), & if \;\; i = j \\ \mathrm{p}(\mathrm{r}_i) \cdot \mathrm{p}(\mathrm{r}_j) + \mathrm{p}(\mathrm{r}_j) \cdot \mathrm{p}(\mathrm{r}_i), & if \;\; i \neq j \end{cases}$$

In the case of $i \neq j$, the frequency count of the two residue pairs $(\mathrm{r}_i, \mathrm{r}_j)$ and $(\mathrm{r}_j, \mathrm{r}_i)$ in R2R contact are summed and considered to be the frequency count of the residue pair $(\mathrm{r}_i, \mathrm{r}_j)$ in R2R contact. Hence, in calculating the probability of observing a residue pair $(\mathrm{r}_i, \mathrm{r}_j)$ in R2R contact, we need to sum the probability of observing the two residue pairs $(\mathrm{r}_i, \mathrm{r}_j)$ and $(\mathrm{r}_j, \mathrm{r}_i)$ in R2R contact.

Here, the set of all mutually exclusive residue pairs is $C = \{(\mathrm{r}_i, \mathrm{r}_j) | 1 \leq i \leq |\sum_R|, 1 \leq j \leq i\}$. Note that each type of residue pair is mutually independent. Let X_{r_i,r_j} be a random variable representing the occurrence of a residue pair $(\mathrm{r}_i, \mathrm{r}_j)$ in R2R contact. The standard residual[24] Z_{r_i,r_j} is thus defined as follows.

$$Z_{r_i,r_j} = \frac{X_{r_i,r_j} - E[X_{r_i,r_j}]}{\sqrt{E[X_{r_i,r_j}]}}$$

We model the random process of N observations of residue pairs in R2R-contact with $|C|$ types, as if extracting N balls (with $k = |C|$ types) from a bag with replacement. In other words, it is a multinomial distribution. $E[X_{r_i,r_j}]$, i.e. the expected value of X_{r_i,r_j} is thus $N \cdot \mathrm{p}(\mathrm{r}_i, \mathrm{r}_j)$. Z_{r_i,r_j} is thus as written as follows:

$$Z_{r_i,r_j} = \frac{X_{r_i,r_j} - N \cdot \mathrm{p}(\mathrm{r}_i, \mathrm{r}_j)}{\sqrt{N \cdot \mathrm{p}(\mathrm{r}_i, \mathrm{r}_j)}}$$

For a more precise analysis, standard residual has to be adjusted by its variance[24]. The adjusted standard residual s_{r_i,r_j}, is defined as:

$$s_{r_i,r_j} = \frac{Z_{r_i,r_j}}{\sqrt{Var(Z_{r_i,r_j})}}$$

We compute $Var\left(Z_{r_i,r_j}\right)$ as follows:

$$Var\left(Z_{r_i,r_j}\right) = Var(\frac{1}{\sqrt{N \cdot \mathrm{p}(\mathrm{r}_i, \mathrm{r}_j)}}(X_{r_i,r_j} - N \cdot \mathrm{p}(\mathrm{r}_i, \mathrm{r}_j)))$$

$$Var\left(Z_{r_i,r_j}\right) = \frac{1}{N \cdot \mathrm{p}(\mathrm{r}_i, \mathrm{r}_j)} Var\left(X_{r_i,r_j} - N \cdot \mathrm{p}(\mathrm{r}_i, \mathrm{r}_j)\right)$$

$$Var\left(Z_{r_i,r_j}\right) = \frac{1}{N \cdot \mathrm{p}(\mathrm{r}_i, \mathrm{r}_j)} Var\left(X_{r_i,r_j}\right)$$

$$Var\left(Z_{r_i,r_j}\right) = \frac{1}{N \cdot \mathrm{p}(\mathrm{r}_i, \mathrm{r}_j)} N \cdot \mathrm{p}(\mathrm{r}_i, \mathrm{r}_j) \left(1 - \mathrm{p}(\mathrm{r}_i, \mathrm{r}_j)\right)$$

$$Var\left(Z_{r_i,r_j}\right) = 1 - \mathrm{p}(\mathrm{r}_i, \mathrm{r}_j)$$

Hence, the adjusted standard residual is derived as:

$$s_{r_i,r_j} = \frac{Z_{r_i,r_j}}{\sqrt{1 - \mathrm{p}(\mathrm{r}_i, \mathrm{r}_j)}}$$

Putting it altogether, the adjusted standard residual is derived as:

$$s_{r_i,r_j} = \frac{X_{r_i,r_j} - N \cdot \mathrm{p}(\mathrm{r}_i, \mathrm{r}_j)}{\sqrt{N \cdot \mathrm{p}(\mathrm{r}_i, \mathrm{r}_j) \cdot \left(1 - \mathrm{p}(\mathrm{r}_i, \mathrm{r}_j)\right)}}$$

1.5. R2R-I Predictor Construction and Operation

Given two protein sequences, a sequence based R2R-I prediction is the process of predicting all R2R-I between two interacting proteins. An illustration is given in Fig. S1.5.

R2R-I is referred to as a residue in one protein interacting with a residue in another protein. R2R-I occurs usually when the two residues are under close contact. Previous studies[3,15,20] suggested that if the contact distance between two residues is below a certain threshold (such as 6Å[3,15,20]), R2R-I is likely to occur. Hence, in our machine learning experiments, all R2R-C pairs, those with contact distance $< 6\dot{A}$, were marked as positive (+ve) R2R-I, otherwise ($\geq 6\dot{A}$) as negative (-ve) R2R-I. For each PPI pair with only sequence information available, all residues pairs between the two proteins are denoted as candidate R2R-I.

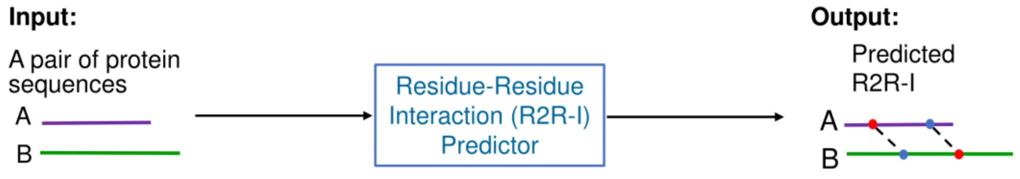

Figure S1.5. R2R-I Prediction Problem Definition. Given two protein sequences A and B, R2R-I prediction between protein sequences A and B is to predict the R2R-I between them when PPI takes place

R2R-I Predictor Construction (Training phase)

Intrinsically, given two residues located on two protein sequences, the R2R-I predictor is a binary classifier to predict whether they can interact. Its construction (Fig. S1.6) is described as below.

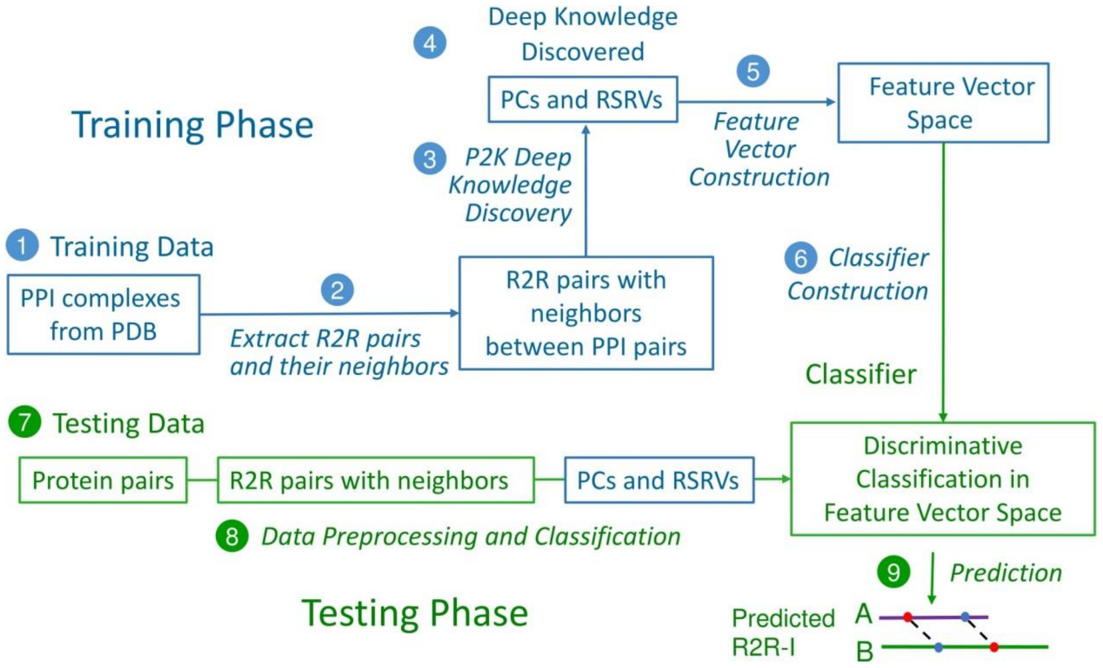

Figure S1.6. An Overview pf P2K Deep Knowledge Directed R2R-I Prediction. A new machine learning method using the deep knowledge discovered to direct the construction of a predictor for R2R-I prediction between interacting

protein sequences. The key steps are marked in circled numerals. **(1) Collection of Positive/Negative R2R-I in PPI Complexes from PDB for Experimental Testing.** R2R pairs (a pair of residues residing on two different protein sequences) in PPI complexes were obtained from PDB. Previous studies[3,15,20] suggested that if the contact distance between two residues is below a threshold (such as 6Å[3,15,20]), R2R-I is likely to occur in the R2R pair. Hence, in our experiments, all R2R pairs, with contact distance $< 6\dot{A}$, were marked as having positive (+ve) R2R-I, otherwise ($\geq 6\dot{A}$) as having negative (-ve) R2R-I. See Definition 1 in Supplement Note 1 for details. **(2) Acquisition of Deep Knowledge Discovered via P2K.** We first obtained via P2K the deep knowledge, consisting of PCs and their corresponding RSRVs with top variance, on the +ve R2R-I. **(3) Extraction of Neighbors of Positive/Negative R2R-I.** For each positive/negative R2R-I taken in step 1, we exploited the R2R-I knowledge of their neighbors via FV construction in machine learning. Fig. 7a gives an example of a R2R pair (A-V) with three neighbors on both sides. **(4) Construction of the FV for R2R-Is**. For each positive/negative R2R-I, we applied the deep knowledge (the more precise disentangled statistical measures procured in PCs and RSRVs not only to the central R2R pair (A-V) but also to its six neighbors (three on each side) to build up its FV (an example is in Fig.7a). Fig. 7b depicts the transformation of the central R2R pair (A-V) with its neighboring residues into a 126-dimension FV. **(5) Construction of the FV Space**. We obtained the FV for R2R-I predictor construction from all positive/negative R2R-Is. **(6) R2R-I Predictor Construction**. From the FV Space obtained in step 5, we constructed a classifier through training, based on the training set with +ve and –ve R2R-I in the FV Space. **(7) Procurement of Protein Pairs for Testing.** Given two input protein sequences, we took all R2R pairs between them for R2R-I prediction. **(8) Obtaining and Inputing the FVs for Prediction**. Each R2R pair between the two proteins was transformed into an FV (like that in Fig. 7b) but without class labels. We inputted these FVs into the R2R-I predictor. **(9) The R2R-I predictor then assigned each FV a score**. We then outputted the R2R pairs with top scores as the predicted R2R-Is. An illustration is given in Fig. S1.8 in Supplement Note 1.

(1) **Collection of Positive/Negative R2R-I in PPI Complexes from PDB for Prediction Experiments.** R2R pairs (a pair of residues residing on two different protein sequences) in PPI complexes were obtained from the PDB. In our experiments, we used the protein-protein docking benchmark dataset (DBD) version 4.0[20]. Previous studies[1,2,20] suggested that if the contact distance between two residues is below a threshold (such as 6Å[1,2,20]), R2R-I is likely to occur in the R2R pair. Hence, in our experiments, all R2R pairs, with contact distance $< 6\dot{A}$, were marked as having positive (+ve) R2R-I, while those with contact distances of $\geq 6\dot{A}$ were considered to have negative (-ve) R2R-I.

(2) **Acquisition of Deep Knowledge Discovered via P2K.** We first obtained via P2K the deep knowledge, consisting of PCs and their corresponding RSRVs with top variance, on the +ve R2R-I pairs. In this study, the top six PCs with a total variance of approximately 80% was selected to direct the predictor construction.

(3) **Extraction of Neighbors of Positive/Negative R2R-I Pairs.** For each positive/negative R2R-I pairs chosen in step 1, we exploited the R2R-I knowledge of their neighbors via feature vector construction using the practice adopted in machine learning. Fig. S1.7 (a) gives an example of a R2R pair (A-V) with three neighbors on both sides.

(4) **Construction of the Feature Vector (FV) for R2R-Is**. For each positive/negative R2R-I, we applied deep knowledge, which incorporates the more precise disentangled statistical measures procured in PCs and RSRVs not only to the central R2R pair (A-V) but also to its six neighbors (three on each side) to build up its FV (an example is presented in Fig. S1.7 (a)). Fig S1.7 (b) shows how the central R2R pair (A-V) with its neighboring residues were transformed into a 126-dimension FV. Features 1 to 84 would be the top 6 PC projection coordinates of the 14 residue alphabets (6x14=84), while features 85 to 126 would be the average RSRVs of the top 6 PCs obtained for each of the 7 R2R pairs (P-E, V-F, K-A, A-V, A-L, F-T and V-I) (6 x 7=42) Hence, the total number of features is 84+42=126, which is the dimension of the FV.

(5) Construction of the Feature Vector Space. From all positive/negative R2R-Is, we obtained their FV for R2R-I predictor construction subsequently.

(6) R2R-I Predictor Construction. From the FV Space obtained in step 5, we constructed an extra decision tree classifier[45] (one variant of the widely used classifier random forests) with 1000 trees through training using the machine learning package scikit-learn 0.18.2[46] under default parameters based on the training set with +ve and –ve R2R-I in the FV Space

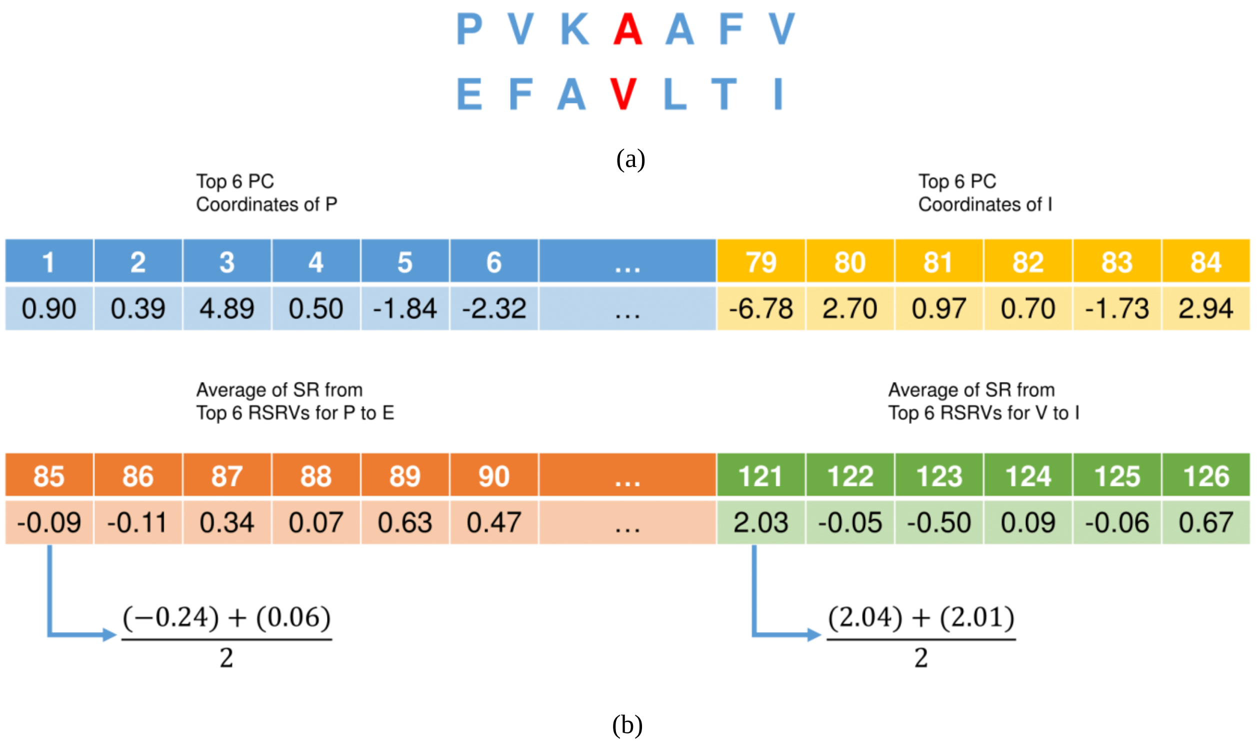

Figure S1.7. An Overview of P2K Deep Knowledge Directed R2R-I Prediction. **(a)** The central R2R pair with its neighbors. This figure gives an example of a R2R pair (A-V) with its neighbors **(b)** A 126-dimensional feature vector (FV) transformed from the R2R-pair with its neighbors shown in (a). Features 1 to 84 (6x14) would be in the projected values of the top 6 PCs of the 14 residue alphabets, while features 85 to 126 would be the average RSRVs of the top 6PCs obtained for each of the 7 R2R pairs (P-E, V-F, K-A, A-V, A-L, F-T and V-I) (6x7=42). Hence, the total number of features is 84+42=126.

Fig. S1.7 (b) gives an example of how the +ve R2R-I (A-V) with its neighboring residues are transformed into a 126-dimension FV. Features 1 to 84 would be the top 6 PC projections of the 14 residue alphabets, while features 85 to 126 would be the average SR from the top 6 RSRVs obtained for each of the R2R pairs (P-E, V-F, K-A, A-V, A-L, F-T and V-I). Hence, the total number of features was 126. A technical detail provided here is that the boundary cases which have no neighboring residues are considered as missing values. Missing values are handled by setting zeros.

R2R-I Predictor Operation in the Testing phase.

The R2R-I predictor constructed could be used for predicting R2R-I between two input protein sequences. This Testing Phase is described in the continuing steps

(7) **Protein Pairs for Testing.** Given two input protein sequences, we took all R2R pairs between them for R2R-I prediction.

(8) **Obtain and Input the FVs for Prediction**. Each R2R pair between the two proteins was transformed into an FV (like that in Fig. S1.7 (b)), but without class labels. We inputted these FVs into the R2R-I predictor.

(9) **The R2R-I predictor then assigned each FV a score.** We then outputted the R2R pairs with top scores as the predicted R2R-Is. An illustration is given in Fig. S1.8 in Supplement Note 1.

An illustration example is provided in Fig. S1.8. A pair of input proteins, say Protein A: SC and Protein B: KLC, is given. All R2R pairs between them are enumerated. The R2R pairs are (S, K), (S, L), (S, C), (C, K), (C, L) and (C, C). All of these R2R pairs are then transformed into a FV. These R2R pairs are then inputted to the R2R-I predictor to obtain a score. The R2R pairs with top scores are outputted.

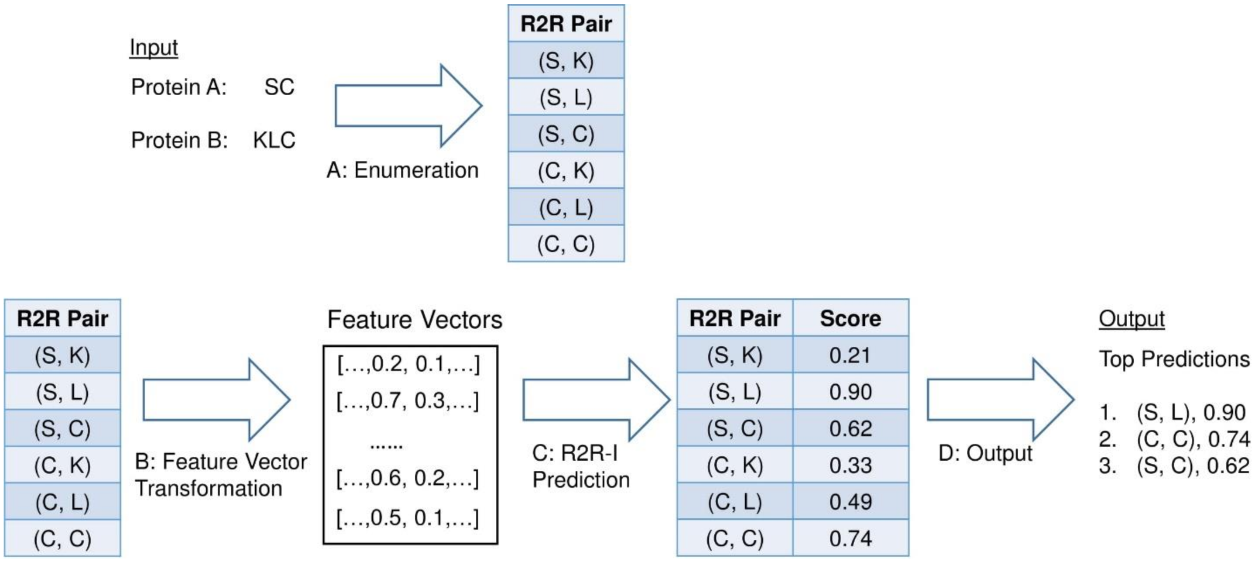

Figure S1.8. A diagram depicting how a R2R-I predictor is operated (Testing Phase) A R2R-I predictor is applied to a pair of protein sequences (Protein A and protein B) to predict the R2R-I between them.

1.6. Illustration of the input and Output R2R-I Predictor.

A real case study is conducted on the target complex 1GL1-A:I, which is one of the 52 PPI complexes newly introduced in protein-protein docking benchmark dataset version 4.0 (abbreviated as DBD 4.0[4]).

Input:

R2R-I predictor needs to have input of two protein sequences. Hence, in the case study, only the two protein sequence chains of the target complex 1GL1-A:I were inputted into the R2R-I predictor. It should be noted that the R2R-I predictor does not need the users to input any structures to function.

>1GL1-A
CGVPAIQPVLSGLSRIVNGEEAVPGSWPWQVSLQDKTGFHFCGGSLINENWVVTAAHCGVTTSD
VVVAGEFDQGSSSEKIQKLKIAKVFKNSKYNSLTINNDITLLKLSTAASFSQTVSAVCLPSASDDF
AAGTTCVTTGWGLTRYTNANTPDRLQQASLPLLSNTNCKKYWGTKIKDAMICAGASGVSSCMG
DSGGPLVCKKNGAWTLVGIVSWGSSTCSTSTPGVYARVTALVNWVQQ

>1GL1-I
EISCEPGKTFKDKCNTCRCGADGKSAACTLKACPNQ

Output:

R2R-I predictor outputs a score of every pair residues between the two protein sequences. The higher the score, the more likely the pair is having interaction. The score is between 0 and 1 inclusively. The top 10 output of R2R-I prediction on the two protein sequence chains of the target complex 1GL1-A:I is illustrated in Table S1.2.

Table S1.2: Top 10 prediction results of the R2R-I predictor on the target complex 1GL1-A:I, which is one of the 52 PPI complexes newly introduced in protein-protein docking benchmark dataset version 4.0 (abbreviated as DBD 4.0[4]). It should be noted that the position starts from 0. Also, in our case study, only the top 5 predictions were considered as positive prediction, while the remaining were considered to be negative predictions.

PairID	ChainID	Position	Residue	ChainID	Position	Residue	Probability	Prediction
7014	A	194	S	I	30	K	0.59	1
7008	A	194	S	I	24	S	0.548	1
7013	A	194	S	I	29	L	0.548	1
1471	A	40	F	I	31	A	0.54667	1
6943	A	192	G	I	31	A	0.531	1
6942	A	192	G	I	30	K	0.521	0
7012	A	194	S	I	28	T	0.513	0
7737	A	214	W	I	33	P	0.509	0
6905	A	191	M	I	29	L	0.494	0
6907	A	191	M	I	31	A	0.491	0
…	…	…	…	…	…	…	…	…

1.7. References in Supplement Note 1

1. Arkin, M. R., Tang, Y. & Wells, J. A. Small-molecule inhibitors of protein-protein interactions: Progressing toward the reality. *Chemistry and Biology* **21,** 1102–1114 (2014).

2. Zhao, Y., Aguilar, A., Bernard, D. & Wang, S. Small-molecule inhibitors of the MDM2-p53 protein-protein interaction (MDM2 inhibitors) in clinical trials for cancer treatment. *Journal of Medicinal Chemistry* **58,** 1038–1052 (2015).

3. Afsar Minhas, F. ul A., Geiss, B. J., Ben-Hur, A. & Minhas, F. U. A. A. PAIRpred: partner-specific prediction of interacting residues from sequence and structure. *Proteins* **82,** 1142–55 (2014).

4. Ezkurdia, I. *et al.* Progress and challenges in predicting protein-protein interaction sites. *Brief. Bioinform.* **10,** 233–246 (2009).

5. Pierce, B. G. *et al.* ZDOCK server: Interactive docking prediction of protein-protein complexes and symmetric multimers. *Bioinformatics* **30,** 1771–1773 (2014).

6. Szilagyi, A. & Zhang, Y. Template-based structure modeling of protein-protein interactions. *Current Opinion in Structural Biology* **24,** 10–23 (2014).

7. Hamer, R., Luo, Q., Armitage, J. P., Reinert, G. & Deane, C. M. i-Patch: interprotein contact prediction using local network information. *Proteins* **78,** 2781–97 (2010).

8. Hopf, T. A. *et al.* Sequence co-evolution gives 3D contacts and structures of protein complexes. *Elife* **3,** e03430 (2014).

9. Wang, H. *et al.* InSite: a computational method for identifying protein-protein interaction binding sites on a proteome-wide scale. *Genome Biol.* **8,** R192 (2007).

10. Amos-Binks, A. *et al.* Binding site prediction for protein-protein interactions and novel motif discovery using re-occurring polypeptide sequences. *BMC Bioinformatics* **12,** 225 (2011).

11. González, A. J., Liao, L. & Wu, C. H. Prediction of contact matrix for protein-protein interaction. *Bioinformatics* **29,** 1018–1025 (2013).

12. Du, T., Liao, L., Wu, C. & Sun, B. Prediction of residue-residue contact matrix for protein-protein interaction with Fisher score features and deep learning. *Methods* (2016).

13. Friedrich, T., Pils, B., Dandekar, T., Schultz, J. & Müller, T. Modelling interaction sites in protein domains with interaction profile hidden Markov models. *Bioinformatics* **22,** 2851–2857 (2006).

14. Stein, A., C??ol, A. & Aloy, P. 3did: Identification and classification of domain-based interactions of known three-dimensional structure. *Nucleic Acids Res.* **39,** (2011).

15. Ahmad, S. & Mizuguchi, K. Partner-aware prediction of interacting residues in protein-protein complexes from sequence data. *PLoS One* **6,** e29104 (2011).

16. Aumentado-Armstrong, T. T., Istrate, B. & Murgita, R. A. Algorithmic approaches to protein-protein interaction site prediction. *Algorithms Mol. Biol.* **10,** 7 (2015).

17. Wang, W., Yang, Y., Yin, J. & Gong, X. Different protein-protein interface patterns predicted by different machine learning methods. *Sci. Rep.* **7,** (2017).

18. Fout, A., Shariat, B., Byrd, J. & Ben-Hur, A. Protein Interface Prediction using Graph Convolutional Networks. in *Advances in Neural Information Processing Systems 30* 6512–6521

(2017).

19. Berman, H. M. *et al.* The Protein Data Bank. *Nucleic Acids Res.* **28,** 235–242 (2000).

20. Glaser, F., Steinberg, D. M., Vakser, I. A. & Ben-Tal, N. Residue frequencies and pairing preferences at protein-protein interfaces. *Proteins Struct. Funct. Genet.* **43,** 89–102 (2001).

21. Shlens, J. A Tutorial on Principal Component Analysis. *ArXiv* 1–13 (2014). doi:10.1.1.115.3503

22. Crowley, P. B. & Golovin, A. Cation-π interactions in protein-protein interfaces. *Proteins Struct. Funct. Bioinforma.* **59,** 231–239 (2005).

23. Winter, C., Henschel, A., Tuukkanen, A. & Schroeder, M. Protein interactions in 3D: From interface evolution to drug discovery. *Journal of Structural Biology* **179,** 347–358 (2012).

24. Haberman, S. J. The Analysis of Residuals in Cross-Classified Tables. *Biometrics* **29,** 205 (1973).

25. Hwang, H., Vreven, T., Janin, J. & Weng, Z. Protein-protein docking benchmark version 4.0. *Proteins Struct. Funct. Bioinforma.* **78,** 3111–3114 (2010).

26. Geurts, P., Ernst, D. & Wehenkel, L. Extremely randomized trees. *Mach. Learn.* **63,** 3–42 (2006).

27. Pedregosa, F., Varoquaux, G. & Gramfort, A. Scikit-learn: Machine learning in Python. *J. Mach.* (2011).

Supplement Note 2: Related work

In this study, R2R-I refers to residues binding between two separate proteins in PPI. In practice, the binding in R2R-I is often considered that the residues in the two proteins are in close contact[1,2]. A high-level definition of R2R-I prediction is: *"Given two proteins A and B, predict which residues in protein A in close contact with residues in protein B, assuming proteins A and B can interact"*[1,2]. Such prediction is also referred to as partner-specific R2R-I prediction. There are four types of computational methods geared towards predicting R2R-I.

The first type is computational docking[3], which requires unbound structures of the target proteins to simulate whether they can interact based on physiochemical properties such as shape complementarity, electrostatics, and biochemical information[4]. However, computational docking is applicable only if the protein structures are available. It should be noted that not many protein sequences have corresponding unbound structures, where the structure to sequence ratio was found to be 0.13%[5]. Recently, template-based methods have been described[6] that map protein sequences to homologous structures to perform docking.

The second type[7,8] is based on co-evolution conjecture, which requires external software such as PSI-Blast[9] to create a multiple sequence alignment (MSA) separately for both proteins. It conjectures that, in PPI between two protein chains, mutations on a protein chain are often compensated by correlated mutations in another protein chain. By creating a MSA separately for both proteins, statistically associated columns are then predicted to be in spatial proximity. It should be noted that the quality of MSA relies on whether or not there is adequate sequence homology.

The third type[10,11] leverages statistical measures derived from external protein databases. InSite[10] takes a library of conserved sequence motifs and a library of motif-motif interaction as input to predict site-to-site interaction between proteins. Sites are considered as interacting if their motifs are similar to interacting motifs in the motif interacting library. PIPE-Sites[11] takes a dataset of PPI as input and predicts site-to-site interaction if those sites are found to be frequently co-occurring in the dataset; the effectiveness of this method depends on the quality of the database. It should be noted that in this context[10,11], a site is referred to as a region on a protein sequence instead of between two interacting residues. Hence, an R2R-I pair is unable to be pinpointed by these methods.

The fourth type[1,2,12,13] is based on machine learning. Taking a dataset of PPI complexes as input for both interacting pairs and non-interacting pairs in building the prediction models, the machine learning method first derives a variety of features, either from structures or MSA created from PSI-Blast external software[9]. Prediction is then achieved if the same type of features can be extracted from the two input proteins. The method[2] is structure-based if it requires structures from the two input proteins. On the other hand, the method[1,2] is sequence-based as it requires only sequences from the two input proteins. Recently there are methods[12,13] that leverage interaction profiles of Hidden Markov Models (imHMMs)[14], which describe domain-domain interaction, have been used to predict R2R-I in PPI, where these imHMMs are obtained from an external database termed 3DID[15]. All these existing methods based on sequence information[1,2] rely on feature engineering over external knowledge of the sequences, such as using external software to obtain features of the input sequences. The key drawback is that a large amount of time is needed in the trail-and-error in the engineering of features, i.e. selecting the optimum combinations of features[16] (e.g. surface accessibility, hydrophobicity, charge…). There is a recent review[16] on the commonly used features for R2R-I prediction. One recent attempt[17] is to use different R2R-I predictors for different types of PPI but it requires the users to have prior knowledge over the type of PPI of the input protein sequences. Another recent attempt[18] is to adopt deep learning using graph convolutional neuron network, where high-end graphical processing units are required during its development. In this study, Pattern-to-Knowledge (P2K), moves in a new direction to predict R2R-I between two proteins based only on sequence information, leveraging the deep knowledge discovered from R2R contact (R2R-C) data as features to build machine learning models.

The entire process does not require neither time-consuming feature engineering over external knowledge on the sequences nor high-end hardware equipment during the construction of the machine learning models. In this paper, we compared with PPiPP[1] in our experiments because it is the closest counterpart available, as it is 1) requiring only sequence input; 2) do not require the users to have prior information over the type of PPI of the input protein sequences; 3) do not require hand-end graphical processing units in neither constructing nor using the machine learning models; 4) providing an easy-to-use web interface. PPiPP[1] is the only available exisiting software meeting these four crieria.

References in Supplement Note 2

1. Ahmad, S. & Mizuguchi, K. Partner-aware prediction of interacting residues in protein-protein complexes from sequence data. *PLoS One* **6,** e29104 (2011).
2. Afsar Minhas, F. ul A., Geiss, B. J., Ben-Hur, A. & Minhas, F. U. A. A. PAIRpred: partner-specific prediction of interacting residues from sequence and structure. *Proteins* **82,** 1142–55 (2014).
3. Pierce, B. G. *et al.* ZDOCK server: Interactive docking prediction of protein-protein complexes and symmetric multimers. *Bioinformatics* **30,** 1771–1773 (2014).
4. Ezkurdia, I. *et al.* Progress and challenges in predicting protein-protein interaction sites. *Brief. Bioinform.* **10,** 233–246 (2009).
5. Wozniak, P. P. & Kotulska, M. Characteristics of protein residue-residue contacts and their application in contact prediction. *J. Mol. Model.* **20,** 2497 (2014).
6. Szilagyi, A. & Zhang, Y. Template-based structure modeling of protein-protein interactions. *Current Opinion in Structural Biology* **24,** 10–23 (2014).
7. Hamer, R., Luo, Q., Armitage, J. P., Reinert, G. & Deane, C. M. i-Patch: interprotein contact prediction using local network information. *Proteins* **78,** 2781–97 (2010).
8. Hopf, T. A. *et al.* Sequence co-evolution gives 3D contacts and structures of protein complexes. *Elife* **3,** e03430 (2014).
9. Altschul, S. F. *et al.* Gapped BLAST and PSI-BLAST: A new generation of protein database search programs. *Nucleic Acids Research* **25,** 3389–3402 (1997).
10. Wang, H. *et al.* InSite: a computational method for identifying protein-protein interaction binding sites on a proteome-wide scale. *Genome Biol.* **8,** R192 (2007).
11. Amos-Binks, A. *et al.* Binding site prediction for protein-protein interactions and novel motif discovery using re-occurring polypeptide sequences. *BMC Bioinformatics* **12,** 225 (2011).
12. González, A. J., Liao, L. & Wu, C. H. Prediction of contact matrix for protein-protein interaction. *Bioinformatics* **29,** 1018–1025 (2013).
13. Du, T., Liao, L., Wu, C. & Sun, B. Prediction of residue-residue contact matrix for protein-protein interaction with Fisher score features and deep learning. *Methods* (2016).
14. Friedrich, T., Pils, B., Dandekar, T., Schultz, J. & Müller, T. Modelling interaction sites in protein domains with interaction profile hidden Markov models. *Bioinformatics* **22,** 2851–2857 (2006).
15. Stein, A., C??ol, A. & Aloy, P. 3did: Identification and classification of domain-based interactions of known three-dimensional structure. *Nucleic Acids Res.* **39,** (2011).
16. Aumentado-Armstrong, T. T., Istrate, B. & Murgita, R. A. Algorithmic approaches to protein-protein interaction site prediction. *Algorithms Mol. Biol.* **10,** 7 (2015).
17. Wang, W., Yang, Y., Yin, J. & Gong, X. Different protein-protein interface patterns predicted by different machine learning methods. *Sci. Rep.* **7,** (2017).
18. Fout, A., Shariat, B., Byrd, J. & Ben-Hur, A. Protein Interface Prediction using Graph Convolutional Networks. in *Advances in Neural Information Processing Systems 30* 6512–6521 (2017).

Supplement Note 3: Exploratory Experiment on Dataset 618

While the main text furnishes a brief analysis of the exploratory experiment on Dataset 618 (Definition 2, Supplement Note 1), this document provides a comprehensive analysis. For ease of understanding and completeness, we restate certain parts as presented in the main text.

The structure of Supplement Note 3 is described as below.

Section 3.1 – "Introduction" provides the background and an overview of the exploratory experiment on Dataset 618.

Section 3.2 – "Analysis of the projections of r-vectors on PC1 and their re-projections on RSRV1" provides a comprehensive analysis of the projections of r-vectors on PC1 and their re-projections on RSRV1.

Section 3.3 – "Analysis of the projections of r-vectors on PC2 and their re-projections on RSRV2" provides a comprehensive analysis of the projections of r-vectors on PC2 and their re-projections on RSRV2.

Section 3.4 – "Analysis of the projections of r-vectors on PC3 and their re-projections on RSRV3" provides a comprehensive analysis of the projections of r-vectors on PC3 and their re-projections on RSRV3

Section 3.5 – "Analysis of the projections of r-vectors on PC4 and their re-projections on RSRV4" provides a comprehensive analysis of the projections of r-vectors on PC4 and their re-projections on RSRV4.

Section 3.6 – "Analysis of the projections of r-vectors on PC5 and their re-projections on RSRV5" provides a comprehensive analysis of the projections of r-vectors on PC5 and their re-projections on RSRV5.

Section 3.7 – "Analysis of the projections of r-vectors on PC6 and their re-projections on RSRV6" provides a comprehensive analysis of the projections of r-vectors on PC6 and their re-projections on RSRV6.

Section 3.8 – "Discussion" provides an overview of the analysis and made a summary.

Section 3.9 – "References in Supplement Note 3" provides the references used in this document.

3.1. Introduction

The projections in PCs 1-6, covering about 80% of the data variance of SRV, are shown in Fig. S3.1-3.6 (i). For each PC, on the negative side, the projections with 1 threshold distance away from the mean are colored green, while those on the positive side with 1 threshold distance away from the mean are colored red. The remaining projections are colored blue. The threshold is chosen to be the standard deviation of the projections in PC1. The corresponding RSRVs are shown in Fig. S3.1-3.6 (ii). The yellow-shaded values signify positive statistical significance at a confidence interval of 95% (>1.96) while green-shaded values signify negative statistical significance at a confidence interval of 95% (<-1.96). Blank-shaded values signify statistically insignificant. We enclosed the significant residue(s) (r-vector(s)) in the PC and the corresponding RSRVs in boxes (referred to as C-box) of the same color.

For each significant residue in the PCs, its statistical R2R-I preference in RSRVs is enclosed by boxes with the same colored border as the one enclosing it in the PC. We denote these boxes with colored borders as colored boxes (C-boxes).

Though most of the distinct residues in the top 6 PCs are found significant only in one PC and its corresponding RSRV, we noted some interesting exceptions (PC3 and RSRV3). We also noticed that a few residues (like V, E and W) appear in more than one RSRVs --- an indication of multiple interacting functionality in the 3-D environment. Here we list the major findings. Experimental and observational details are elaborated later in the discussion.

3.2. Analysis of the projections of r-vectors on PC1 and their re-projections on RSRV1

The projections of r-vectors in SRV on PC1, covering a variance of 31.51%, are shown in Fig. S3.1 (i). We found that the green groups (L V I F M A) are associated with a high hydrophobicity scale (or hydropathy index)[1] and the red groups (T S N D) are associated with a low hydrophobicity scale. The higher the hydrophobicity scale a residue has, the more hydrophobic it is[1]. We also enclosed M with a purple C-box, as M is close to the green group and the hydrophobicity scale of M is also relatively high. Table S3.1 summarizes the hydrophobicity scale of the residues in these two groups. It should be noted that hydrophobicity reflects the tendency for a residue to be found on the surface of a protein[2,3]. Studies reported that hydrophobicity plays an important role in intermolecular recognition processes[4] such as PPI[5], and hydrophobicity has been used for predicting protein interface residues[6–9]. When we compared the projection coordinates of this group of residues in PC1, a disentangled statistical/functional subspace, with their hydrophobicity scale in Table S3.1 taken from the literature[1], we were surprised to find that the statistical strength and the functional strength astoundingly follow the same order. This is a perfect case to show that physical knowledge could be reflected by statistics obtained from disentangled subspace from R2R-C data.

RSRV1 shows the re-projections of the projections of r-vectors in SRV on PC1 in Fig. S3.1 (ii). We observed that the members of each group are statistically more preferred with their own members but not those of others. The positive significant statistical association among the red group (D S T N) could be attributed to hydrophilic-hydrophilic interaction[10]. Likewise, those association among the green group (L V I F M A) could be attributed to hydrophobic-hydrophobic interaction[10]. It has also been reported that surface patches with high hydrophobicity are energetically unfavorable in an aqueous solution, but favorable when in contact with other hydrophobic surfaces[11]. We would also like to point out that **as shown in the SRV (Fig. 3) the SR of S-T is 1.23 (insignificant), whereas in RSRV1 (Fig. S3.1 (ii)) the SR of S-T and T-S are 2.02 (significant) and 1.91 (almost significant), respectively. This indicates that disentanglement helps to reveal that S-T does interact in** hydrophilic setting**.**

Table S3.1. A summary of the projection of colored residues in PC1 and their hydrophobicity scale[1].

**Residue**	**Color**	**Hydrophobicity scale[1]**
L	Green	3.8
V	Green	4.2
I	Green	4.5
F	Green	2.8
M	Green	1.9
A	Green	1.8
T	Red	-0.7
S	Red	-0.8
N	Red	-3.5
D	Red	-3.5

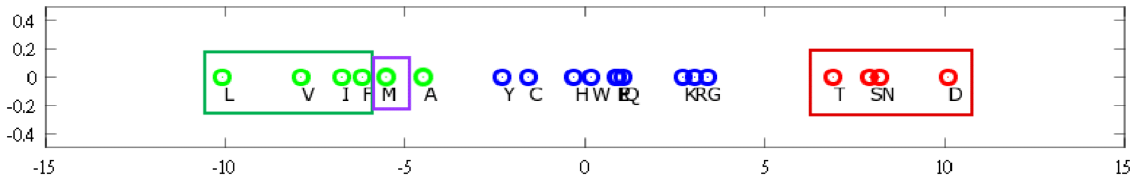

(i) PC1 (Variance = 31.5150%)

	I	V	L	F	C	M	A	G	T	S	W	Y	P	H	E	Q	D	N	K	R
**I**	1.69	2.01	2.62	1.54	0.23	1.39	1.05	-1.15	-2.14	-2.42	-0.25	0.44	-0.45	-0.12	-0.44	-0.50	-3.02	-2.50	-0.97	-1.05
**V**	2.04	2.41	3.12	1.86	0.35	1.69	1.30	-1.26	-2.40	-2.73	-0.22	0.59	-0.45	-0.06	-0.43	-0.50	-3.43	-2.82	-1.05	-1.14
**L**	2.79	3.25	4.15	2.56	0.64	2.34	1.84	-1.40	-2.84	-3.25	-0.07	0.95	-0.37	0.13	-0.35	-0.43	-4.15	-3.37	-1.12	-1.25
**F**	1.63	1.92	2.47	1.48	0.29	1.35	1.04	-0.99	-1.89	-2.15	-0.16	0.48	-0.35	-0.04	-0.34	-0.39	-2.71	-2.23	-0.82	-0.90
**C**	0.61	0.69	0.84	0.57	0.25	0.53	0.45	-0.10	-0.34	-0.41	0.13	0.30	0.08	0.16	0.08	0.07	-0.56	-0.43	-0.05	-0.07
**M**	1.45	1.72	2.23	1.32	0.22	1.20	0.91	-0.95	-1.78	-2.01	-0.19	0.40	-0.36	-0.07	-0.35	-0.40	-2.53	-2.08	-0.79	-0.86
**A**	1.19	1.40	1.80	1.08	0.21	0.98	0.76	-0.72	-1.37	-1.56	-0.11	0.35	-0.25	-0.02	-0.24	-0.28	-1.97	-1.61	-0.59	-0.65
**G**	-0.84	-0.99	-1.27	-0.77	-0.17	-0.70	-0.55	0.48	0.94	1.07	0.06	-0.26	0.15	0.00	0.15	0.18	1.35	1.10	0.39	0.43
**T**	-2.06	-2.36	-2.95	-1.91	-0.65	-1.77	-1.44	0.69	1.64	1.91	-0.18	-0.85	0.01	-0.31	0.00	0.06	2.49	1.99	0.51	0.59
**S**	-2.23	-2.58	-3.25	-2.06	-0.61	-1.89	-1.52	0.93	2.02	2.33	-0.07	-0.84	0.15	-0.22	0.14	0.20	3.01	2.42	0.72	0.82
**W**	-0.22	-0.22	-0.22	-0.23	-0.23	-0.23	-0.23	-0.24	-0.24	-0.24	-0.23	-0.23	-0.23	-0.23	-0.23	-0.23	-0.24	-0.24	-0.24	-0.24
**Y**	0.57	0.68	0.90	0.51	0.05	0.46	0.34	-0.45	-0.80	-0.90	-0.12	0.12	-0.20	-0.08	-0.19	-0.21	-1.11	-0.92	-0.38	-0.41
**P**	-0.17	-0.20	-0.27	-0.15	-0.01	-0.14	-0.10	0.14	0.24	0.27	0.04	-0.04	0.06	0.02	0.06	0.07	0.34	0.28	0.12	0.12
**H**	0.05	0.07	0.11	0.04	-0.06	0.03	0.00	-0.15	-0.22	-0.24	-0.09	-0.04	-0.10	-0.08	-0.10	-0.11	-0.28	-0.25	-0.14	-0.15
**E**	-0.44	-0.47	-0.53	-0.43	-0.30	-0.41	-0.38	-0.18	-0.08	-0.06	-0.26	-0.32	-0.24	-0.27	-0.24	-0.24	0.00	-0.05	-0.19	-0.19
**Q**	-0.51	-0.55	-0.62	-0.49	-0.33	-0.47	-0.43	-0.16	-0.04	-0.01	-0.27	-0.36	-0.25	-0.29	-0.25	-0.24	0.07	0.00	-0.19	-0.18
**D**	-3.01	-3.46	-4.32	-2.79	-0.94	-2.58	-2.10	1.03	2.42	2.82	-0.25	-1.24	0.04	-0.44	0.02	0.10	3.68	2.93	0.76	0.88
**N**	-2.38	-2.74	-3.44	-2.20	-0.70	-2.03	-1.64	0.89	2.03	2.35	-0.14	-0.94	0.09	-0.30	0.08	0.14	3.05	2.44	0.68	0.78
**K**	-0.98	-1.10	-1.32	-0.93	-0.45	-0.87	-0.75	0.05	0.41	0.51	-0.28	-0.53	-0.20	-0.33	-0.21	-0.19	0.73	0.54	-0.02	0.01
**R**	-0.94	-1.07	-1.32	-0.87	-0.34	-0.81	-0.67	0.23	0.63	0.75	-0.14	-0.42	-0.06	-0.20	-0.06	-0.04	1.00	0.78	0.15	0.19

(ii) RSRV1 (Re-projection of projections in PC1)

Figure S3.1 (i). The projections of r-vectors in SRV on PC1 with variance = 31.51%. We observed two groups: hydrophilic (red) and hydrophobic (green). **(ii)** In the RSRV1, those SRs that are positively statistically significant are colored yellow, and those that are negatively significant are colored green. In this RSRV, residues in each group only interact with members within their own group but not with members of the other group; and all the SRs not in these two groups have low values since they are both statistically insignificant and/or functionally orthogonal to this corresponding PC**.**

Table S3.2. A summary of the projection of all residues in PC1 and their hydrophobicity scale[1].

Residue	Hydrophobicity scale [1]	PC1 Projection
I	4.5	-6.77
V	4.2	-7.89
L	3.8	-10.07
F	2.8	-6.21
C	2.5	-1.56
M	1.9	-5.69
A	1.8	-4.47
G	-0.4	3.40
T	-0.7	6.91
S	-0.8	7.91
W	-0.9	0.18
Y	-1.3	-2.30
P	-1.6	0.90
H	-3.2	-0.31
E	-3.5	0.85
Q	-3.5	1.06
D	-3.5	10.08
N	-3.5	8.20
K	-3.9	2.74
R	-4.5	3.03

A summary of the projection of all residues in PC1 and their hydrophobicity scale[1] is shown in Table S3.2. The Pearson correction between the PC1 projection and the hydropathy scale has been computed. The value of R is -0.8063, indicating a strong negative correlation. In other words, the projected coordinates in PC1 are found to be negatively correlated with the hydropathy scale. The value of R^2, the coefficient of determination, is 0.6501. The P-Value of the Pearson correction is **1.8E-05 < 0.05**. This indicates that the correlation between the PC1 projection and the hydropathy scale is statistically significant. In addition, the Spearman correlation between the PC1 projection and the hydropathy scale has a value of R = -0.71774 and the two-tailed P-value is **3.7E-04 < 0.05**.

3.3. Analysis of the projections of r-vectors on PC2 and their re-projections on RSRV2

The projections of r-vectors in SRV on PC2, covering a data variance of 14.77%, are shown in Fig. S3.2 (i). We observed that the projection of C (in green) is highly distinctive. We also enclosed the projection of S in purple. In RSRV2 (Fig. S3.2 (ii)), the SRs of R2R pairs C-C and C-S are both positively significant, and the C-C pair is also highly distinctive. This could be attributed to the fact that C-C could form a disulfide bond which plays a major role in PPI[12]. This is also consistent with the observation in a previous study[10]. In addition, the finding that C is likely to interact with S via a C-S hydrogen bond (H-bond) was reported only in a recent study[13]. As we observed, both C and S in PC2 are not very close to each other. This suggests that they may not be that close in functionality. Note that the SR of C-S in SRV as shown in Fig. 3 is 1.53, which shows no statistical significance, but their significance is revealed in RSRV2 as 2.47. Furthermore, we have a very significant and revealing observation from this PC. We found that the projected coordinates of the r-vectors on it are distinct from other PCs in such a way that there is no residue with a projected coordinate opposite that of "C" on the other side of the Mean. This result suggests that the disulfide bond may be the dominant molecular force between C and C, with no obvious counterparts. Since this is the natural result of functional disentanglement, it renders another case showing that deep knowledge can be unveiled by P2K from R2R-C data. It also furnishes additional information that biochemists and other scientists can use to acquire a more complete picture of R2R-I.

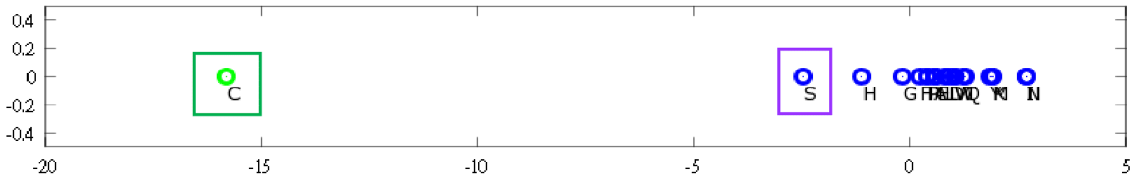

(i) PC2 (Variance = 14.7750%)

	I	V	L	F	C	M	A	G	T	S	W	Y	P	H	E	Q	D	N	K	R
I	0.17	-0.06	-0.09	-0.17	-2.39	0.06	-0.13	-0.23	-0.03	-0.54	-0.06	0.05	-0.15	-0.36	-0.12	-0.03	-0.08	0.17	0.06	-0.14
V	-0.04	-0.11	-0.12	-0.15	-0.81	-0.08	-0.13	-0.16	-0.10	-0.26	-0.11	-0.08	-0.14	-0.20	-0.13	-0.10	-0.12	-0.05	-0.08	-0.14
L	0.08	0.03	0.03	0.01	-0.48	0.06	0.02	0.00	0.04	-0.07	0.03	0.06	0.01	-0.03	0.02	0.04	0.03	0.08	0.06	0.01
F	-0.13	-0.12	-0.12	-0.12	-0.02	-0.13	-0.12	-0.11	-0.12	-0.10	-0.12	-0.13	-0.12	-0.11	-0.12	-0.12	-0.12	-0.13	-0.13	-0.12
C	-2.41	-0.84	-0.62	-0.07	15.06	-1.67	-0.38	0.30	-1.05	2.47	-0.83	-1.59	-0.22	1.18	-0.45	-1.05	-0.72	-2.40	-1.64	-0.25
M	0.10	-0.05	-0.07	-0.13	-1.62	0.03	-0.09	-0.16	-0.03	-0.38	-0.05	0.02	-0.11	-0.25	-0.09	-0.03	-0.06	0.10	0.03	-0.11
A	-0.04	-0.06	-0.07	-0.08	-0.31	-0.05	-0.07	-0.08	-0.06	-0.12	-0.06	-0.05	-0.07	-0.10	-0.07	-0.06	-0.07	-0.04	-0.05	-0.07
G	-0.03	0.01	0.02	0.03	0.44	-0.01	0.02	0.04	0.00	0.10	0.01	-0.01	0.03	0.07	0.02	0.00	0.01	-0.03	-0.01	0.03
T	-0.08	-0.17	-0.19	-0.22	-1.09	-0.13	-0.20	-0.24	-0.16	-0.36	-0.17	-0.13	-0.21	-0.29	-0.20	-0.16	-0.18	-0.08	-0.13	-0.21
S	-0.56	-0.29	-0.26	-0.16	2.43	-0.44	-0.22	-0.10	-0.33	0.27	-0.29	-0.42	-0.19	0.05	-0.23	-0.33	-0.27	-0.56	-0.43	-0.19
W	-0.14	-0.20	-0.20	-0.22	-0.80	-0.16	-0.21	-0.24	-0.19	-0.32	-0.20	-0.17	-0.22	-0.27	-0.21	-0.19	-0.20	-0.14	-0.16	-0.22
Y	0.13	-0.01	-0.03	-0.09	-1.51	0.06	-0.06	-0.12	0.01	-0.32	-0.01	0.06	-0.07	-0.20	-0.05	0.01	-0.03	0.13	0.06	-0.07
P	0.06	0.04	0.04	0.04	-0.12	0.05	0.04	0.03	0.05	0.01	0.04	0.05	0.04	0.02	0.04	0.05	0.04	0.06	0.05	0.04
H	-0.31	-0.17	-0.15	-0.10	1.22	-0.24	-0.13	-0.07	-0.19	0.12	-0.17	-0.24	-0.12	0.01	-0.14	-0.19	-0.16	-0.31	-0.24	-0.12
E	-0.23	-0.25	-0.25	-0.26	-0.47	-0.24	-0.26	-0.27	-0.25	-0.30	-0.25	-0.24	-0.26	-0.28	-0.26	-0.25	-0.25	-0.23	-0.24	-0.26
Q	-0.14	-0.23	-0.24	-0.27	-1.09	-0.18	-0.25	-0.29	-0.22	-0.41	-0.23	-0.19	-0.26	-0.34	-0.25	-0.22	-0.23	-0.14	-0.18	-0.26
D	-0.24	-0.29	-0.30	-0.32	-0.79	-0.27	-0.31	-0.33	-0.29	-0.40	-0.29	-0.27	-0.31	-0.36	-0.30	-0.29	-0.30	-0.24	-0.27	-0.31
N	0.17	-0.06	-0.09	-0.17	-2.39	0.07	-0.12	-0.22	-0.03	-0.54	-0.06	0.05	-0.15	-0.35	-0.11	-0.03	-0.07	0.17	0.06	-0.14
K	-0.05	-0.20	-0.22	-0.28	-1.73	-0.12	-0.25	-0.31	-0.18	-0.52	-0.20	-0.13	-0.26	-0.40	-0.24	-0.18	-0.21	-0.05	-0.12	-0.26
R	-0.14	-0.15	-0.15	-0.16	-0.28	-0.15	-0.16	-0.16	-0.15	-0.18	-0.15	-0.15	-0.16	-0.17	-0.16	-0.15	-0.15	-0.14	-0.15	-0.16

(ii) RSRV2 (Re-projection of projections in PC2)

Figure S3.2 (i). The projections of r-vectors in SRV on PC2 cover a data variance of 14.77%. There are two distinctive projected r-vectors corresponding to C and S (green and purple); **(ii)** In RSRV2, only C-C (distinct) and C-S (less distinct) are boxed. While C interacting with C via a disulfide bond is known to be important in PPI[12], the finding that C is able to interact with S was confirmed only in a recent study[13]. Both C and S are on the same side in the PC box, as they are related through their interaction with C and they are not opposite R2R-I. However, the finding that they are not very close in the PC might suggest that they may not be that close in functionality.

3.4. Analysis of the projections of r-vectors on PC3 and their re-projections on RSRV3

The projections of r-vectors in SRV on PC3, covering a data variance of 9.65%, are shown in Fig. S3.3 (i). We observed that there are two groups, the green group (A, V) and the red group (P,Y,W). We conjectured that these two groups correspond to aliphatic-hydrophobic and aromatic groups, respectively, where only residues within, but not between groups could interact[14]. In RSRV3 (Fig. S3.3 (ii)), most of the SRs in the C-boxes are slightly below 1.96, except for that between A-A, which is conjectured to be an aliphatic-aliphatic interaction[14]. We reasoned that the statistical preference between A and V could be attributed to aliphatic-hydrophobic interaction[14], while those between Y and W could be attributed to aromatic-aromatic interaction[14]. As for proline (P), with SR > 1.62, we found that it can interact with aromatic residues through CH/π interaction[15]. As for isoleucine (I), although it is aliphatic, its SRs are low in PC3 and RSRV3.

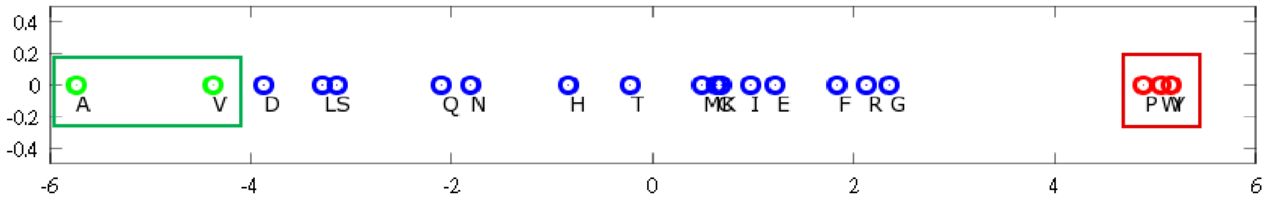

(i) PC3 (Variance = 9.6500%)

	I	V	L	F	C	M	A	G	T	S	W	Y	P	H	E	Q	D	N	K	R
**I**	-0.13	-0.54	-0.45	-0.06	-0.15	-0.16	-0.64	-0.02	-0.22	-0.44	0.18	0.19	0.17	-0.27	-0.11	-0.36	-0.50	-0.34	-0.15	-0.04
**V**	-0.46	1.21	0.87	-0.73	-0.35	-0.31	1.63	-0.89	-0.09	0.82	-1.73	-1.77	-1.68	0.10	-0.54	0.50	1.05	0.41	-0.37	-0.82
**L**	-0.23	1.03	0.77	-0.43	-0.15	-0.11	1.34	-0.55	0.05	0.74	-1.18	-1.20	-1.14	0.20	-0.28	0.49	0.91	0.43	-0.16	-0.50
**F**	0.02	-0.73	-0.57	0.14	-0.03	-0.05	-0.92	0.21	-0.15	-0.56	0.59	0.61	0.57	-0.23	0.05	-0.41	-0.66	-0.37	-0.02	0.18
**C**	0.19	-0.08	-0.03	0.23	0.17	0.17	-0.15	0.26	0.13	-0.02	0.40	0.40	0.39	0.10	0.20	0.03	-0.06	0.05	0.18	0.25
**M**	-0.11	-0.33	-0.28	-0.07	-0.12	-0.13	-0.39	-0.05	-0.16	-0.28	0.06	0.07	0.06	-0.18	-0.10	-0.23	-0.31	-0.22	-0.12	-0.06
**A**	-0.48	1.72	1.28	-0.84	-0.34	-0.28	2.29	-1.05	0.01	1.22	-2.16	-2.21	-2.09	0.26	-0.58	0.79	1.52	0.67	-0.36	-0.96
**G**	0.21	-0.73	-0.54	0.36	0.15	0.12	-0.97	0.45	0.00	-0.52	0.93	0.95	0.90	-0.11	0.25	-0.33	-0.65	-0.28	0.16	0.41
**T**	-0.24	-0.18	-0.20	-0.25	-0.24	-0.23	-0.17	-0.25	-0.23	-0.20	-0.28	-0.28	-0.28	-0.22	-0.24	-0.21	-0.19	-0.21	-0.24	-0.25
**S**	-0.34	0.86	0.61	-0.54	-0.27	-0.24	1.16	-0.65	-0.08	0.58	-1.26	-1.28	-1.22	0.06	-0.40	0.35	0.74	0.28	-0.28	-0.60
**W**	0.13	-1.88	-1.47	0.46	0.01	-0.05	-2.40	0.65	-0.31	-1.42	1.67	1.71	1.61	-0.55	0.23	-1.02	-1.69	-0.92	0.02	0.57
**Y**	0.26	-1.78	-1.36	0.59	0.14	0.08	-2.30	0.79	-0.19	-1.31	1.82	1.86	1.76	-0.42	0.36	-0.91	-1.58	-0.80	0.15	0.71
**P**	0.38	-1.53	-1.14	0.69	0.26	0.21	-2.02	0.87	-0.04	-1.09	1.84	1.88	1.78	-0.26	0.47	-0.72	-1.35	-0.62	0.28	0.80
**H**	-0.14	0.16	0.10	-0.19	-0.12	-0.11	0.23	-0.22	-0.07	0.09	-0.37	-0.37	-0.36	-0.04	-0.15	0.03	0.13	0.02	-0.12	-0.20
**E**	-0.17	-0.69	-0.59	-0.08	-0.20	-0.22	-0.82	-0.03	-0.29	-0.57	0.23	0.24	0.21	-0.35	-0.15	-0.47	-0.64	-0.44	-0.20	-0.06
**Q**	-0.42	0.35	0.20	-0.55	-0.37	-0.35	0.55	-0.62	-0.25	0.18	-1.01	-1.03	-0.99	-0.16	-0.46	0.02	0.28	-0.02	-0.38	-0.59
**D**	-0.59	0.87	0.57	-0.82	-0.50	-0.46	1.24	-0.96	-0.26	0.54	-1.70	-1.73	-1.65	-0.10	-0.65	0.25	0.73	0.17	-0.51	-0.90
**N**	-0.32	0.35	0.22	-0.43	-0.28	-0.26	0.53	-0.50	-0.17	0.20	-0.84	-0.85	-0.82	-0.09	-0.35	0.07	0.29	0.03	-0.29	-0.47
**K**	-0.24	-0.54	-0.48	-0.19	-0.26	-0.27	-0.61	-0.17	-0.31	-0.47	-0.02	-0.01	-0.03	-0.34	-0.23	-0.41	-0.51	-0.40	-0.26	-0.18
**R**	-0.01	-0.85	-0.68	0.13	-0.06	-0.08	-1.07	0.21	-0.20	-0.66	0.64	0.65	0.61	-0.29	0.03	-0.49	-0.77	-0.45	-0.05	0.18

(ii) RSRV3 (Re-projection of projections in PC3)

Figure S3.3 (i). The projections of r-vectors in SRV on PC3 covering a data variance of 9.65%. There are two distinct groups (A, V) boxed in green and (P, Y, W) boxed in red. **(ii).** In RSRV3, we observed that only residues within, but not between groups, could interact. Note that the majority of their SRs is below 1.96 but above 1.62, indicating that their interactions are weaker. We conjectured that these two groups (A,V) and (P, Y, W) are corresponding to aliphatic-hydrophobic and aromatic groups, respectively[14].

3.5. Analysis of the projections of r-vectors on PC4 and their re-projections on RSRV4

The projections of r-vectors in SRV on PC4, covering a data variance of 9.43%, are shown in Fig. S3.4 (i). We observed that there are two groups, green (H, L) and red (K, E, V) that could interact with their own group members but not between groups. **(ii)** In RSRV4 (Fig. S3.4 (ii)), The SRs of V-V, V-E are strong (SR=2.15 and 2.03 respectively). With SR = 2.77 and 2.70 respectively, we observed strong contact preferences of H-L and H-H. We speculated that interaction within the (K, E, V) group and the (H, L) group are due to hydrogen bonding (H-bonding). For both groups, we found examples of 3D PPI complex to support our speculation that the interaction within the (K, E, V) group and the (H, L) group are due to hydrogen bonding (H-bonding). For the (K, E, V) group, they are 1) 1pma-C159E and 1pma-D60E, 2) 1gua-A37E and 1gua-B69V, 3) 1hsl-A25E and 1hsl-B39K, 4) 1mdt-A13V and 1mdt-B13V, 5) 1cdc-A39V and 1cdc-B51K, 6) 1dyn-A15K and 1dyn-B15K; while the group (H, L) related to another type of H-bond, as found in 1) 1bhm-A121H and 1bhm-B121H, and 2) 1sft-A5H and 1sft-B67L. Our reasoning is that H-bonds are essential in determining binding specificity[16,17] and provide favorable free energy for the binding[18], while unfulfilled bonds, introduced by the presence of hydrogen bonding residues without a bonding partner, could destabilize binding[19]. The contrast in energetics contributes to a high selectivity in matching the H-bonds between proteins, and confers binding specificity. Hence, we conjectured that H-bonds are determining the binding specificity within these groups[16].

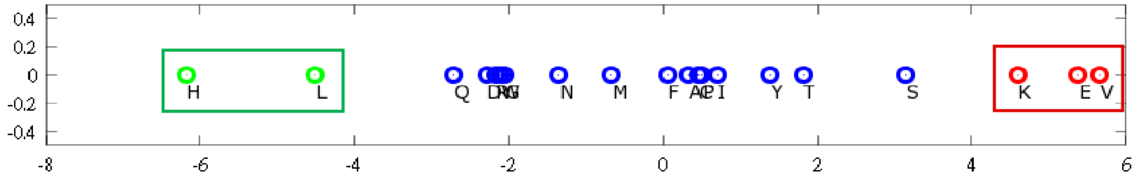

(i) PC4 (Variance = 9.4309%)

	I	V	L	F	C	M	A	G	T	S	W	Y	P	H	E	Q	D	N	K	R
**I**	-0.17	0.04	-0.40	-0.20	-0.18	-0.23	-0.19	-0.29	-0.12	-0.06	-0.29	-0.14	-0.18	-0.47	0.03	-0.32	-0.30	-0.26	0.00	-0.30
**V**	0.13	2.15	-1.99	-0.13	0.03	-0.43	-0.02	-0.99	0.58	1.12	-1.01	0.41	0.05	-2.67	2.03	-1.26	-1.09	-0.71	1.71	-1.04
**L**	-0.23	-1.91	1.53	-0.02	-0.16	0.23	-0.11	0.70	-0.61	-1.06	0.72	-0.47	-0.17	2.10	-1.82	0.92	0.78	0.46	-1.55	0.74
**F**	-0.12	-0.14	-0.10	-0.12	-0.12	-0.11	-0.12	-0.11	-0.12	-0.13	-0.11	-0.12	-0.12	-0.09	-0.14	-0.10	-0.11	-0.11	-0.14	-0.11
**C**	0.16	0.29	0.02	0.14	0.15	0.12	0.15	0.09	0.19	0.22	0.08	0.18	0.15	-0.03	0.28	0.07	0.08	0.10	0.26	0.08
**M**	-0.19	-0.47	0.11	-0.15	-0.17	-0.11	-0.17	-0.03	-0.25	-0.33	-0.02	-0.23	-0.18	0.21	-0.46	0.01	-0.01	-0.07	-0.41	-0.02
**A**	-0.07	0.02	-0.15	-0.08	-0.07	-0.09	-0.07	-0.11	-0.05	-0.03	-0.11	-0.06	-0.07	-0.18	0.01	-0.12	-0.12	-0.10	0.00	-0.12
**G**	-0.07	-0.86	0.75	0.03	-0.04	0.14	-0.01	0.36	-0.25	-0.46	0.37	-0.18	-0.04	1.01	-0.81	0.47	0.40	0.25	-0.69	0.38
**T**	-0.14	0.48	-0.80	-0.22	-0.17	-0.31	-0.19	-0.49	0.00	0.17	-0.49	-0.05	-0.17	-1.01	0.45	-0.57	-0.52	-0.40	0.35	-0.50
**S**	0.03	1.15	-1.14	-0.11	-0.02	-0.28	-0.05	-0.59	0.28	0.58	-0.60	0.19	-0.01	-1.52	1.09	-0.74	-0.64	-0.43	0.91	-0.62
**W**	-0.35	-1.19	0.53	-0.24	-0.31	-0.12	-0.29	0.11	-0.54	-0.77	0.12	-0.47	-0.32	0.81	-1.14	0.23	0.15	0.00	-1.01	0.14
**Y**	-0.04	0.42	-0.53	-0.10	-0.06	-0.17	-0.08	-0.30	0.06	0.19	-0.30	0.02	-0.06	-0.68	0.40	-0.36	-0.32	-0.23	0.32	-0.31
**P**	0.06	0.22	-0.12	0.04	0.05	0.01	0.04	-0.03	0.09	0.14	-0.04	0.08	0.05	-0.17	0.21	-0.06	-0.04	-0.01	0.19	-0.04
**H**	-0.41	-2.70	1.99	-0.11	-0.30	0.22	-0.24	0.86	-0.92	-1.54	0.89	-0.73	-0.32	2.77	-2.57	1.16	0.97	0.54	-2.20	0.92
**E**	0.00	1.90	-1.99	-0.24	-0.09	-0.52	-0.14	-1.05	0.43	0.94	-1.07	0.27	-0.07	-2.63	1.79	-1.30	-1.14	-0.78	1.49	-1.09
**Q**	-0.43	-1.47	0.67	-0.29	-0.38	-0.14	-0.35	0.15	-0.66	-0.94	0.16	-0.57	-0.39	1.02	-1.41	0.29	0.20	0.00	-1.25	0.18
**D**	-0.45	-1.35	0.50	-0.33	-0.41	-0.20	-0.38	0.05	-0.65	-0.89	0.06	-0.58	-0.41	0.80	-1.30	0.17	0.09	-0.08	-1.16	0.07
**N**	-0.28	-0.81	0.28	-0.21	-0.25	-0.13	-0.24	0.02	-0.40	-0.54	0.03	-0.35	-0.25	0.46	-0.78	0.09	0.05	-0.05	-0.69	0.03
**K**	-0.06	1.57	-1.78	-0.28	-0.14	-0.52	-0.19	-0.97	0.30	0.74	-0.99	0.16	-0.13	-2.34	1.48	-1.19	-1.05	-0.74	1.22	-1.01
**R**	-0.28	-1.09	0.58	-0.17	-0.24	-0.05	-0.21	0.17	-0.46	-0.68	0.18	-0.39	-0.24	0.85	-1.04	0.28	0.21	0.06	-0.91	0.20

(ii) RSRV4 (Re-projection of projections in PC4)

Figure S3.4 (i). The projections of r-vectors in SRV on PC4 covering a data variance of 9.43%. There are two conjectured groups: H-bond 1 (red) and H-bond 2 (green). **(ii)** RSRV4 shows that only members in the same group could interact but not those between groups.

3.6. Analysis of the projections of r-vectors on PC5 and their re-projections on RSRV5

The projections of r-vectors in SRV on PC5 covering a data variance of 7.30% are shown in Fig. S3.5 (i). We observed that there are two distinctive groups: The green group (R) and the red group (W, E, D), which correspond with positive and negative charge, respectively. While the residue W is not usually listed as a residue with negative charge, a recent finding reported that its surface is negatively charged [15], complying with the R2R-I knowledge (SR=2.08) we discovered. Note that the projection of K is close to that of R, and it also contains positive charge. In RSRV5 (Fig. S3.5 (ii)), it is obvious that residues of opposite charges attract and of same charges repel (reflected by the yellow and green cells, respectively). We noticed that in PC5 and RSVR5, which are dominated by charged residues, the SR values for all other non-charged residues are insignificant and irrelevant, an obvious manifestation of the result of disentanglement.

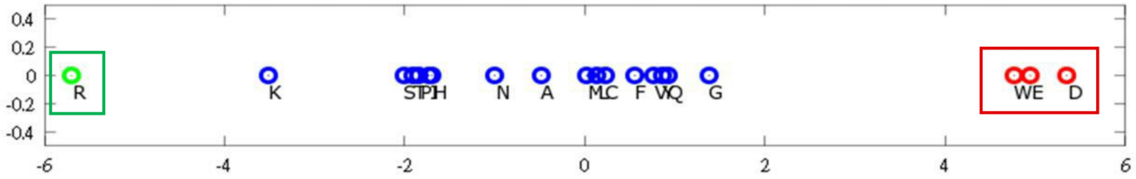

(i) PC5 (Variance = 7.3018%)

	I	V	L	F	C	M	A	G	T	S	W	Y	P	H	E	Q	D	N	K	R
**I**	-0.44	-0.10	-0.18	-0.13	-0.17	-0.20	-0.27	-0.01	-0.46	-0.48	0.45	-0.08	-0.46	-0.44	0.48	-0.08	0.53	-0.34	-0.69	-0.99
**V**	-0.02	-0.22	-0.17	-0.20	-0.17	-0.16	-0.12	-0.26	-0.01	0.00	-0.53	-0.22	-0.01	-0.03	-0.54	-0.23	-0.57	-0.08	0.12	0.28
**L**	0.04	-0.01	0.00	-0.01	0.00	0.00	0.01	-0.03	0.04	0.04	-0.10	-0.02	0.04	0.04	-0.10	-0.02	-0.11	0.02	0.07	0.12
**F**	-0.02	-0.16	-0.12	-0.14	-0.13	-0.12	-0.09	-0.19	-0.01	-0.01	-0.37	-0.16	-0.02	-0.02	-0.38	-0.16	-0.40	-0.06	0.07	0.19
**C**	0.17	0.13	0.14	0.13	0.14	0.14	0.15	0.12	0.17	0.17	0.06	0.13	0.17	0.17	0.06	0.13	0.05	0.16	0.20	0.23
**M**	-0.13	-0.15	-0.15	-0.15	-0.15	-0.15	-0.14	-0.16	-0.13	-0.13	-0.19	-0.15	-0.13	-0.13	-0.20	-0.16	-0.20	-0.14	-0.11	-0.09
**A**	-0.12	-0.06	-0.08	-0.07	-0.07	-0.08	-0.09	-0.05	-0.13	-0.13	0.04	-0.06	-0.13	-0.12	0.04	-0.06	0.05	-0.10	-0.17	-0.22
**G**	0.24	-0.05	0.02	-0.03	0.01	0.03	0.10	-0.13	0.26	0.28	-0.53	-0.06	0.26	0.24	-0.55	-0.07	-0.60	0.16	0.46	0.72
**T**	-0.49	-0.12	-0.21	-0.15	-0.20	-0.23	-0.30	-0.02	-0.51	-0.53	0.48	-0.10	-0.50	-0.48	0.51	-0.09	0.57	-0.38	-0.75	-1.08
**S**	-0.39	-0.01	-0.10	-0.04	-0.09	-0.12	-0.20	0.09	-0.42	-0.44	0.61	0.01	-0.41	-0.39	0.63	0.01	0.70	-0.28	-0.67	-1.00
**W**	0.46	-0.54	-0.29	-0.45	-0.32	-0.24	-0.03	-0.79	0.53	0.58	-2.15	-0.58	0.51	0.45	-2.22	-0.60	-2.38	0.17	1.18	2.06
**Y**	0.03	-0.17	-0.12	-0.15	-0.12	-0.11	-0.07	-0.22	0.05	0.06	-0.49	-0.18	0.04	0.03	-0.51	-0.18	-0.54	-0.03	0.18	0.36
**P**	-0.21	0.14	0.05	0.11	0.07	0.04	-0.04	0.23	-0.24	-0.26	0.72	0.16	-0.23	-0.21	0.74	0.17	0.80	-0.11	-0.47	-0.78
**H**	-0.33	0.02	-0.07	-0.01	-0.05	-0.08	-0.16	0.11	-0.35	-0.37	0.59	0.03	-0.35	-0.32	0.61	0.04	0.67	-0.23	-0.58	-0.89
**E**	0.48	-0.59	-0.32	-0.50	-0.36	-0.27	-0.05	-0.85	0.55	0.60	-2.30	-0.63	0.52	0.46	-2.38	-0.66	-2.55	0.16	1.24	2.17
**Q**	-0.14	-0.34	-0.29	-0.33	-0.30	-0.28	-0.24	-0.39	-0.12	-0.11	-0.67	-0.35	-0.13	-0.14	-0.69	-0.36	-0.72	-0.20	0.01	0.19
**D**	0.46	-0.67	-0.38	-0.57	-0.42	-0.33	-0.10	-0.95	0.54	0.59	-2.49	-0.71	0.51	0.45	-2.57	-0.74	-2.75	0.13	1.28	2.27
**N**	-0.32	-0.15	-0.19	-0.16	-0.19	-0.20	-0.23	-0.11	-0.33	-0.33	0.12	-0.14	-0.32	-0.31	0.13	-0.14	0.16	-0.27	-0.43	-0.58
**K**	-0.78	-0.08	-0.26	-0.14	-0.23	-0.29	-0.43	0.09	-0.83	-0.86	1.04	-0.06	-0.81	-0.77	1.10	-0.04	1.21	-0.58	-1.29	-1.90
**R**	-0.98	0.20	-0.10	0.10	-0.06	-0.15	-0.39	0.49	-1.06	-1.11	2.08	0.24	-1.03	-0.96	2.17	0.27	2.36	-0.63	-1.82	-2.85

(ii) RSRV5 (Re-projection of projections in PC5)

Figure S3.5 (i). The projections of r-vectors in SRV on PC5 covering a data variance of 7.30%. There are two distinct groups: the green group (R) and the red group (W, E, D), which are found to have positive and negative charge, respectively. While the residue W is not listed as a residue with negative charge in most text books, it was identified as a negative residue due to its negative surface charge, as reported in a recent study[15]. **(ii)** In RSRV5, residues with the opposite charge attract and the same charge repel (shown by SR in yellow and green cells, respectively).

3.7. Analysis of the projections of r-vectors on PC6 and their re-projections on RSRV6

The projections of r-vectors in SRV on PC6, covering a data variance of 6.97%, are shown in Fig. S3.6 (i). We observed that there are two distinctive groups, the green group (N) and the red group (G). In RSRV6 (Fig. 3.6 (ii)), G-G is statistically preferred and G-N is not. Though not significant, the SR of N-N is quite large (1.88). Note that it is reported that interaction involving G is not rationalized[10].

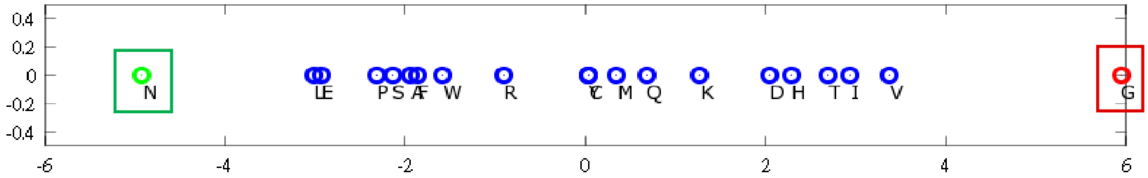

(i) PC6 (Variance = 6.9655%)

	I	V	L	F	C	M	A	G	T	S	W	Y	P	H	E	Q	D	N	K	R
**I**	0.53	0.64	-0.95	-0.67	-0.19	-0.12	-0.68	1.27	0.47	-0.74	-0.59	-0.19	-0.78	0.37	-0.93	-0.03	0.31	-1.43	0.11	-0.43
**V**	0.69	0.81	-1.03	-0.69	-0.15	-0.06	-0.72	1.55	0.62	-0.77	-0.61	-0.15	-0.82	0.50	-1.00	0.04	0.43	-1.57	0.21	-0.42
**L**	-0.76	-0.87	0.78	0.49	-0.01	-0.09	0.50	-1.54	-0.70	0.56	0.41	-0.01	0.60	-0.59	0.76	-0.17	-0.53	1.28	-0.33	0.24
**F**	-0.59	-0.66	0.37	0.18	-0.12	-0.17	0.20	-1.07	-0.55	0.23	0.14	-0.12	0.26	-0.48	0.36	-0.22	-0.44	0.68	-0.32	0.03
**C**	0.15	0.15	0.13	0.14	0.14	0.14	0.14	0.15	0.15	0.14	0.14	0.14	0.14	0.14	0.13	0.14	0.14	0.13	0.14	0.14
**M**	-0.06	-0.05	-0.23	-0.20	-0.15	-0.14	-0.20	0.02	-0.07	-0.21	-0.19	-0.15	-0.21	-0.08	-0.23	-0.13	-0.09	-0.29	-0.11	-0.17
**A**	-0.57	-0.64	0.43	0.23	-0.09	-0.14	0.25	-1.07	-0.53	0.28	0.18	-0.08	0.31	-0.46	0.41	-0.19	-0.42	0.74	-0.29	0.07
**G**	1.53	1.75	-1.50	-0.91	0.05	0.21	-0.95	3.06	1.41	-1.05	-0.77	0.05	-1.14	1.20	-1.45	0.38	1.08	-2.47	0.68	-0.43
**T**	0.44	0.54	-0.92	-0.66	-0.22	-0.15	-0.67	1.13	0.39	-0.72	-0.59	-0.22	-0.76	0.29	-0.90	-0.08	0.24	-1.35	0.06	-0.44
**S**	-0.67	-0.75	0.44	0.22	-0.13	-0.19	0.24	-1.23	-0.63	0.27	0.17	-0.13	0.31	-0.55	0.42	-0.25	-0.51	0.79	-0.36	0.04
**W**	-0.62	-0.68	0.17	0.02	-0.24	-0.28	0.03	-1.02	-0.59	0.05	-0.02	-0.24	0.08	-0.54	0.16	-0.32	-0.50	0.42	-0.40	-0.11
**Y**	-0.10	-0.10	-0.11	-0.11	-0.11	-0.11	-0.11	-0.09	-0.10	-0.11	-0.11	-0.11	-0.11	-0.10	-0.11	-0.10	-0.10	-0.12	-0.10	-0.11
**P**	-0.56	-0.64	0.64	0.41	0.03	-0.04	0.42	-1.16	-0.51	0.46	0.35	0.03	0.50	-0.42	0.62	-0.10	-0.38	1.02	-0.22	0.22
**H**	0.48	0.56	-0.67	-0.45	-0.08	-0.02	-0.46	1.06	0.43	-0.50	-0.39	-0.08	-0.53	0.35	-0.65	0.04	0.31	-1.04	0.16	-0.26
**E**	-0.99	-1.10	0.48	0.20	-0.27	-0.35	0.22	-1.74	-0.93	0.27	0.13	-0.27	0.31	-0.83	0.46	-0.43	-0.77	0.96	-0.58	-0.04
**Q**	-0.11	-0.09	-0.45	-0.39	-0.28	-0.26	-0.39	0.06	-0.13	-0.40	-0.37	-0.28	-0.41	-0.15	-0.45	-0.24	-0.16	-0.56	-0.21	-0.33
**D**	0.20	0.28	-0.86	-0.65	-0.32	-0.26	-0.67	0.73	0.16	-0.70	-0.60	-0.32	-0.73	0.08	-0.84	-0.20	0.04	-1.20	-0.10	-0.48
**N**	-1.44	-1.63	1.08	0.59	-0.21	-0.35	0.62	-2.71	-1.34	0.71	0.47	-0.21	0.78	-1.17	1.04	-0.49	-1.06	1.88	-0.74	0.18
**K**	0.01	0.06	-0.61	-0.49	-0.29	-0.26	-0.50	0.32	-0.01	-0.52	-0.46	-0.29	-0.54	-0.06	-0.60	-0.23	-0.08	-0.81	-0.16	-0.39
**R**	-0.41	-0.44	0.09	0.00	-0.16	-0.19	0.00	-0.66	-0.39	0.02	-0.03	-0.16	0.03	-0.35	0.08	-0.22	-0.33	0.25	-0.27	-0.08

(ii) RSRV6 (Re-projection of projections in PC6)

Figure S3.6 (i). The projections of r-vectors in SRV on PC6 covering a data variance of 6.97%. There are two distinct groups: N (in green) and G (in red). **(ii)** In RSRV6, we observed that each group preferred to interact with others in the same group, but not with those in the other group.

3.8. Discussion

Here we discuss some exceptional and interesting cases that resulted from our observations above.

In the PC1 projection, we observed that there are two strong groups: the hydrophobic group (I, L, V, F, M, A) and the hydrophilic group (T, S, N, D). We observed that the last two residues in the hydrophobic group, apart from the M-L interaction, do not generally exhibit very strong interactions. The most amazing finding is that the PC coordinates of the residue projections in PC1 are astoundingly correlated with their hydrophobic indices (Table S3.1). Another surprising observation is related to the isoleucine residue (I). While both the disentangled statistic results and the chemical findings show prominent associations, it is surprising to find in the report[20] that "*its side chain is very non-reactive and rarely directly involved in protein function such as catalysis, but can be involved in binding/recognition.*" Hence further study seems to be necessary to validate our finding.

We observed that on the whole, the statistically distinctive residue projections on the PC comply with special R2R-I functionality, as reflected by their appearance in unique RSRVs. Examples are residues "C" in PC2/RSRV2 and most of the charged residues (W, D, R; except E in PC5). We also observed that few other residues distinctly appear in more than one of the PCs and RSRVs. Examples are residue V which appears in PC1 (hydrophobic contact) with (I, V, L) and PC4 (special H-bond) with (V, E); residue E with PC5 (charged) and PC4 (special H-Bond) with (V, E); and residue W in PC5 (with negative surface charge) and in PC3 (aromatic contact) with (W, Y, P), though not very strong. These are indications of multiple interacting functionalities in the 3-D environment, and their statistical association with different functionality can be brought forth via disentanglement from the R2R-C data.

As mentioned in the Analysis of R2R-I of residues on PC2 and their re-projections on RSRV2, we found that the projected coordinates of the r-vectors C and S on PC2 are distinct from other PCs since there is no residue with a projected coordinate opposite to that of "C" on the other side of the mean --- suggesting that the disulfide bond in C-C has no obvious counterparts. It also revealed the contact preference of S with C, which was not discovered until recently[13]. It is interesting to observe that the R2R-I of S with C is on the same side of the mean in PC2 but not too close to C, suggesting their R2R-I differences. These observations furnish additional information that biochemists and other investigators can use to acquire a more complete picture of R2R interactions.

Of all these experimental results, the most intriguing case can be found in PC3 and RSRV3. Although its eigenvalue is ranked third among all PCs, a closer look at the distribution of their projections on PC3 show that the distributions are quite broad. This may explain its high eigenvalues. It looks as though the contribution to its high variance is actually due to the aromatic group (W, E, P) and the aliphatic-hydrophobic residue A (due to strong A-A association). Nevertheless, their overall R2R-I SR values are relatively weak compared with other RSRVs. In RSRV3, the SR for V interacting with others in this space is weak.

3.9. Reference in Supplement Note 3

1. Kyte, J. & Doolittle, R. F. A simple method for displaying the hydropathic character of a protein. *J. Mol. Biol.* **157,** 105–132 (1982).
2. JANIN, J. Surface and inside volumes in globular proteins. *Nature* **277,** 491–492 (1979).
3. Rose, G., Geselowitz, A., Lesser, G., Lee, R. & Zehfus, M. Hydrophobicity of amino acid residues in globular proteins. *Science (80-.).* **229,** (1985).
4. Eisenhaber, F. & Argos, P. Hydrophobic regions on protein surfaces: definition based on hydration shell structure and a quick method for their computation. *Protein Eng.* **9,** 1121–33 (1996).
5. Chanphai, P., Bekale, L. & Tajmir-Riahi, H. A. Effect of hydrophobicity on protein–protein interactions. *Eur. Polym. J.* **67,** 224–231 (2015).
6. Xue, L. C., Dobbs, D., Bonvin, A. M. J. J. & Honavar, V. Computational prediction of protein interfaces: A review of data driven methods. *FEBS Lett.* **589,** 3516–3526 (2015).
7. Maheshwari, S. & Brylinski, M. Predicting protein interface residues using easily accessible on-line resources. *Brief. Bioinform.* 1–10 (2015). doi:10.1093/bib/bbv009
8. Esmaielbeiki, R., Krawczyk, K., Knapp, B., Nebel, J. C. & Deane, C. M. Progress and challenges in predicting protein interfaces. *Brief. Bioinform.* **17,** 117–131 (2016).
9. Zhang, J. & Kurgan, L. Review and comparative assessment of sequence-based predictors of protein-binding residues. *Brief. Bioinform.* **320,** 991–1009 (2017).
10. Glaser, F., Steinberg, D. M., Vakser, I. A. & Ben-Tal, N. Residue frequencies and pairing preferences at protein-protein interfaces. *Proteins Struct. Funct. Genet.* **43,** 89–102 (2001).
11. Winter, C., Henschel, A., Tuukkanen, A. & Schroeder, M. Protein interactions in 3D: From interface evolution to drug discovery. *Journal of Structural Biology* **179,** 347–358 (2012).

12. Meitzler, J. L., Hinde, S., Banfi, B., Nauseef, W. M. & Ortiz de Montellano, P. R. Conserved Cysteine Residues Provide a Protein-Protein Interaction Surface in Dual Oxidase (DUOX) Proteins. *J. Biol. Chem.* **288,** 7147–7157 (2013).
13. Hussain, H. B., Wilson, K. A. & Wetmore, S. D. Serine and Cysteine pi-Interactions in Nature: A Comparison of the Frequency, Structure, and Stability of Contacts Involving Oxygen and Sulfur. *Aust. J. Chem.* (2014).
14. Pereira de Araujo, A. F., Pochapsky, T. C. & Joughin, B. Thermodynamics of interactions between amino acid side chains: experimental differentiation of aromatic-aromatic, aromatic-aliphatic, and aliphatic-aliphatic side-chain interactions in water. *Biophys. J.* **76,** 2319–2328 (1999).
15. Zondlo, N. J. Aromatic-proline interactions: Electronically tunable CH/?? interactions. *Acc. Chem. Res.* **46,** 1039–1049 (2013).
16. Fersht, A. R. Basis of biological specificity. *Trends Biochem. Sci.* **9,** 145--147 (1984).
17. Honig, B. & Yang, A. Free Energy Balance in Protein Folding. *Adv. Prot. Chem.* **46,** 27–57 (1995).
18. Xu, D., Lin, S. L. & Nussinov, R. Protein binding versus protein folding: the role of hydrophilic bridges in protein associations. *J. Mol. Biol.* **265,** 68–84 (1997).
19. Byrne, M. P., Manuel, R. L., Lowe, L. G. & Stites, W. E. Energetic contribution of side chain hydrogen bonding to the stability of staphylococcal nuclease. *Biochemistry* **34,** 13949–60 (1995).
20. Betts, M. J. & Russell, R. B. Amino acid properties and consequences of subsitutions. In Bioinformatics for Geneticists. in *Wiley* 311–342 (2003). doi:10.1002/0470867302.ch14

Supplement Note 4: Benchmarking experiments

While the main text furnishes a brief description of the benchmarking experiments on protein-protein docking benchmark dataset version 4.0 (abbreviated as DBD 4.0[1]), this document provides a comprehensive explanation. For ease of understanding and completeness, we restate certain parts as presented in the main text.

The structure of Supplement Note 4 is shown below.

Section 4.1 – "Introduction" provides the background and an overview of the benchmarking experiment.

Section 4.2 – "Experiments" provides all the experimental details and the workflow.

Section 4.3 – "Experimental Results" reports the experimental results and the analysis.

Section 4.4 – "Case Study" provides a case study to demonstrate the effectiveness of P2K.

Section 4.5 – "Discussion" summarizes the analysis on the benchmarking experiments.

Section 4.6 – "References in Supplement Note 4" provides the references used in this document.

4.1. Introduction

We have achieved the discovery of deep knowledge from R2R contact data procured from existing PPI complexes. However, could such discovered knowledge be used for R2R-I prediction? To answer this question, we developed a R2R-I predictor utilizing such information. We devised a new learning method to compose the deep knowledge discovered by P2K in the form of a feature vector for machine learning. Through extensive experiments, we aim to show that we can leverage the deep knowledge discovered to construct machine learning models for R2R-I prediction without feature engineering over external knowledge of the sequences.

4.2. Experiments

To evaluate the effectiveness of statistical measures reflected by PC Projections and RSRVs discovered by P2K in predicting R2R-I between two protein sequences, we conducted benchmark experiments as described below.

4.2.1. Problem definition

As mentioned in Section 5 in Supplement Note 1, the problem of sequence-based R2R-I prediction is defined as follows. Given two protein sequences, a sequence based R2R-I prediction is the process of predicting all R2R-I between two interacting proteins.

4.2.2. Data preprocessing

As mentioned in Definition 2 in Supplement Note 1, R2R-C is referred to as a pair of residues in separate protein chains with the closest Euclidean distance in 3D coordinate space between their C-Beta atoms (C-Alpha atoms for Gly (G))[2]) being less than 6Å[2–4]. Hence, in our machine learning experiments, all R2R-C pairs, whose with contact distance $< 6\dot{A}$, were marked as positive (+ve) R2R-I, otherwise ($\geq 6\dot{A}$) as negative (-ve) R2R-I. For each PPI pair with only sequence information available, all residues pairs between the two proteins are denoted as candidate R2R-I.

4.2.3. Experimental configuration

In our experimental configuration, we used the protein-protein docking benchmark dataset version 4.0 (abbreviated as DBD 4.0[1]), which contains 176 non-redundant PPI complexes, to evaluate and compare our results with those obtained by other methods considered the best to date. We divided the benchmark data into two sets: one was used for training and the other was retained for testing. The training data consisted of the first 124 non-redundant PPI complexes accessible from the Protein Data Bank (PDB)[5], as summarized in Supplement Table 4.1. This set of data is equivalent to protein-protein docking benchmark dataset version 3.0 (abbreviated as DBD 3.0[6]). The testing data was the last 52 non-redundant PPI complexes in DBD 4.0[1], as summarized in Supplement Table 4.2.

Following a previous study[3], in our experiment, given a PPI complex, all pairwise ligand/receptor protein chains were considered. For example, for the case of 1FCC-AB:C, the R2R-I predictor was requested to make prediction on both 1FCC-A:C and 1FCC-B:C, and the prediction performance was evaluated together as a whole for 1FCC-AB:C. Here, 1FCC is the PDB PPI Complex ID. It has three protein chains: A, B, C. "AB:C" means that there could only be 2 possible PPI cases, which are A:C and B:C.

In machine learning, the term AUC (Area Under Curve) refers to the area under a receiver operating characteristic (ROC) curve[7], which is often used for the evaluation of a binary classification system. The higher the AUC value, the better the prediction performance. The maximum value of AUC value is 1.0 whereas the minimum value is 0.0. In our experiments, based on a previous study[3], we evaluated the prediction performance of the sequence-based R2R-I predictor based on AUC.

For training P2K, an extra decision tree classifier[8] (one variant of the widely used classifier random forests) was trained with 1000 trees under default parameter except setting class weight to be balanced using the machine learning package scikit-learn 0.18.2[9].

For our leave-one-complex-out-alone experiment, we trained P2K on the training set and validated it on the validation set. By leave-one-complex-out-alone cross-validation, we mean that in one fold of an experiment, one case in Supplement Table 4.1 is consider to be validation set, while the other cases are considered to be training set. The leave-one-complex-out-alone cross-validation would have 124 folds such that each case would have a chance to be as the validation set. An AUC value is obtained in each fold. The final output is the average of AUC obtained among the 124 folds.

In this paper, we compared with PPiPP[1] in our experiments because it is the closest counterpart available, as it is 1) requiring only sequence input; 2) do not require the users to have prior information over the type of PPI of the input protein sequences; 3) do not require hand-end graphical processing units in neither constructing nor using the machine learning models; 4) providing an easy-to-use web interface. PPiPP[1] is the only available exisiting software meeting these four crieria.

For our comparison with PPiPP[3], we trained P2K on the first 124 PPI complexes in DBD 4.0[1] containing a total of 8,497,005 training samples, as summarized in Supplement Table 4.1. We then applied P2K to predict R2R-I on the last 52 PPI complexes in DBD 4.0[1], as summarized in Supplement Table 4.2. For benchmarking purpose, we compared our performance with PPiPP[3], which was also trained by the same 124 non-redundant PPI complexes and is the one that provides a web interface to access the predictor. It should also be noted that the primary objective of the comparison is only to demonstrate that the PC projections and RSRVs discovered by P2K are effective for R2R-I prediction between two protein sequences.

4.2.4. Experiment rationale

As demonstrated in Supplement Note 3, the R2R-C data procured from 618 protein interacting complexes with 17,278 contact pairs had captured various physiochemical characteristics of the target R2R-I. Our next question is whether the physiochemical characteristics captured can be applied to the neighboring residues of the target R2R-I. The underlying hypothesis is that the neighboring residues could affect the target R2R-I pair. As it is difficult to prove such a hypothesis, we turned to an empirical statistical approach using machine learning. Thus, we designed an experiment to test how much the discovered deep knowledge could help to acquire more R2R-I statistical support from additional data in the cloud. We then incrementally applied the discovered deep knowledge to the neighboring residues of the target candidate R2R-I pair from the PPI data acquired from the PDB in the construction of machine-learning prediction models, and investigated empirically whether additional statistical information could improve the prediction performance.

For a machine-learning perspective, we evaluated different settings of the dimension (the length) of feature vectors. The process of feature vector construction is provided in Section 5 in Supplement Note 1. We hereby provide an example of transforming the candidate interacting segments (PVK**A**AFV, EFA**V**LTI) with the centered residue pair (**A, V**) in bold into discriminating information and incorporate it into a discriminative feature vector. If the number of neighboring residues considered was 3, features 1 to 84 would be the top 6 PC projections of the 14 residue alphabets, while features 85 to 126 would be the average SR from the top 6 RSRVS obtained for each of the R2R pairs (P-E, V-F, K-A, A-V, A-L, F-T and V-I). Hence, the total number of features was 126, as illustrated in Fig. S1.7 in Supplement Note 1.

Similarly, if the number of neighboring residues considered was 2, features 1 to 60 would be the top 6 PC projections of the 10 residue alphabets, while features 61 to 90 would be the average SR from the top 6 RSRVS obtained for each of the R2R pairs (V-F, K-A, A-V, A-L, F-T). Hence, the total number of features is 90. If the number of neighboring residues considered was 1, features 1 to 36 would be the top 6 PC projections of the 6 residue alphabets, while features 37 to 54 would be the average SR from the top 6 RSRVS obtained for each of the R2R pairs (K-A, A-V, A-L). Hence, the total number of features was 54. If the number of neighboring residues considered was 0, features 1 to 12 would be the top 6 PC projections of the 2 residue alphabets, while features 13 to 18 would be the average SR from the top 6 RSRVS obtained for the R2R pair (A-V). Hence, the total number of features was 18. These 4 cases are illustrated in Fig. S4.1.

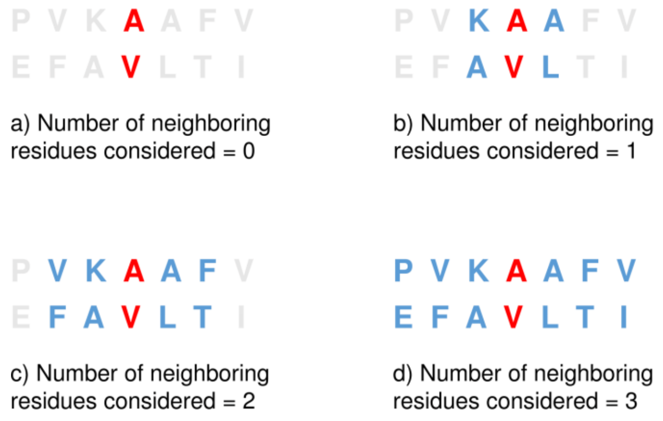

Figure. S4.1. The figure illustrates the 4 cases of the number of neighboring residues to be considered in feature vector construction on the R2R pair (A,V): a) the number of neighboring residues is considered as 0. The total number of features in the feature was 18; b) the number of neighboring residues is considered to be 1. The total number of features in the feature was 54; c) the number of neighboring residues is considered to be 2. The total number of features in the feature was 90; d) the number of neighboring residues is considered to be 3. The total number of features in the feature was 126.

Supplement Table 4.1: A summary of the first 124 non-redundant PPI complexes in protein-protein docking benchmark dataset version 4.0 (abbreviated as DBD 4.0[1]). This set of data is equivalent to protein-protein docking benchmark dataset version 3.0 (abbreviated as DBD 3.0[6]), and was the training data in our experiments. Here the complex column records the PDB PPI Complex ID. For example, 1FCC is the PDB ID. It has three protein chains: A, B, C. "AB:C" means that there could only be 2 possible PPI cases, which are A:C and B:C.

Case	Complex ID	No. of +ve R2R-I	No. of –ve R2R-I	Total
1	1AVX-A:B	20	37820	37840
2	1AY7-A:B	14	8530	8544
3	1BVN-P:T	22	35194	35216
4	1CGI-E:I	29	13691	13720
5	1D6R-A:I	17	12743	12760
6	1DFJ-E:I	15	56529	56544
7	1E6E-A:B	20	51621	51641
8	1EAW-A:B	23	12689	12712
9	1EWY-A:C	15	29679	29694
10	1EZU-C:AB	33	61567	61600
11	1F34-A:B	43	44807	44850
12	1HIA-AB:I	24	10680	10704
13	1MAH-A:F	24	32489	32513
14	1N8O-ABC:E	24	32480	32504
15	1OPH-A:B	19	82481	82500
16	1PPE-E:I	21	6359	6380
17	1R0R-E:I	35	13939	13974
18	1TMQ-A:B	32	54958	54990
19	1UDI-E:I	24	18817	18841
20	1YVB-A:I	25	23174	23199
21	2B42-B:A	27	66949	66976
22	2MTA-HL:A	17	52168	52185
23	2O8V-A:B	17	24715	24732
24	2PCC-A:B	11	31741	31752
25	2SIC-E:I	30	29395	29425
26	2SNI-E:I	29	17571	17600
27	2UUY-A:B	20	11576	11596
28	7CEI-A:B	18	11031	11049
29	1A2K-C:AB	20	48588	48608
30	1AK4-A:D	15	23910	23925
31	1AKJ-AB:DE	13	85259	85272
32	1AZS-AB:C	14	128467	128481
33	1B6C-A:B	17	34865	34882
34	1BUH-A:B	14	20076	20090
35	1E96-A:B	13	32917	32930
36	1EFN-B:A	12	5916	5928
37	1F51-AB:E	22	43175	43197
38	1FC2-C:D	8	8850	8858
39	1FQJ-A:B	20	42141	42161
40	1GCQ-B:C	15	3918	3933
41	1GHQ-A:B	11	39592	39603
42	1GLA-G:F	12	78717	78729
43	1GPW-A:B	18	50582	50600
44	1HE1-C:A	19	23741	23760

45	1I4D-D:AB	11	66010	66021
46	1J2J-A:B	8	6757	6765
47	1K74-AB:DE	17	69679	69696
48	1KAC-A:B	19	22921	22940
49	1KLU-AB:D	10	84860	84870
50	1KTZ-A:B	15	8677	8692
51	1KXP-A:D	23	152839	152862
52	1ML0-A:D	14	23730	23744
53	1QA9-A:B	9	9681	9690
54	1RLB-ABCD:E	16	84548	84564
55	1S1Q-A:B	15	9712	9727
56	1SBB-A:B	12	56870	56882
57	1T6B-X:Y	26	114894	114920
58	1XD3-A:B	29	17146	17175
59	1Z0K-A:B	18	10291	10309
60	1Z5Y-D:E	11	16037	16048
61	1ZHI-A:B	15	24360	24375
62	2AJF-A:E	15	103863	103878
63	2BTF-A:P	15	51832	51847
64	2HLE-A:B	25	24979	25004
65	2HQS-A:H	20	44476	44496
66	2OOB-A:B	9	3159	3168
67	1AHW-AB:C	14	85586	85600
68	1BVK-DE:F	9	28887	28896
69	1DQJ-AB:C	10	54686	54696
70	1E6J-HL:P	14	90076	90090
71	1JPS-HL:T	16	85184	85200
72	1MLC-AB:E	15	55713	55728
73	1VFB-AB:C	9	28758	28767
74	1WEJ-HL:F	13	45435	45448
75	2FD6-HL:U	10	104150	104160
76	2I25-N:L	19	14687	14706
77	2VIS-AB:C	16	115061	115077
78	1BJ1-HL:VW	12	81016	81028
79	1FSK-BC:A	13	68993	69006
80	1I9R-HL:ABC	22	186164	186186
81	1IQD-AB:C	20	63160	63180
82	1K4C-AB:C	16	44377	44393
83	1KXQ-H:A	27	59493	59520
84	1NCA-HL:N	19	165189	165208
85	1NSN-HL:S	15	57807	57822
86	1QFW-HL:AB	6	43531	43537
87	1QFW-IM:AB	13	44706	44719
88	2JEL-HL:P	11	36114	36125
89	1ACB-E:I	24	15159	15183
90	1IJK-A:BC	10	50138	50148
91	1KKL-ABC:H	27	43059	43086
92	1M10-A:B	13	53120	53133
93	1NW9-B:A	23	21635	21658
94	1GP2-A:BG	21	135171	135192
95	1GRN-A:B	23	37604	37627
96	1HE8-B:A	6	124328	124334
97	1I2M-A:B	23	63997	64020

98	1IB1-AB:E	30	80782	80812
99	1K5D-AB:C	32	121056	121088
100	1N2C-ABCD:EF	50	1095950	1096000
101	1WQ1-R:G	21	53099	53120
102	1XQS-A:C	19	43541	43560
103	2CFH-A:C	25	23964	23989
104	2H7V-A:C	12	47870	47882
105	2HRK-A:B	15	21402	21417
106	2NZ8-A:B	21	49362	49383
107	1BGX-HL:T	70	343550	343620
108	1FQ1-A:B	17	53968	53985
109	1PXV-A:C	32	20281	20313
110	1ATN-A:D	23	95695	95718
111	1BKD-R:S	35	72839	72874
112	1DE4-AB:CF	15	471155	471170
113	1EER-A:BC	38	70678	70716
114	1FAK-HL:T	21	62016	62037
115	1H1V-A:G	26	120310	120336
116	1IBR-A:B	24	76920	76944
117	1IRA-Y:X	19	45076	45095
118	1JMO-A:HL	30	104095	104125
119	1R8S-A:E	37	29883	29920
120	1Y64-A:B	22	146294	146316
121	2C0L-A:B	24	35600	35624
122	2OT3-B:A	33	39688	39721
123	1E4K-AB:C	22	74282	74304
124	2HMI-CD:AB	11	428781	428792
Total	-	2456	8494549	8497005

Supplement Table 4.2: A summary of the last 52 non-redundant PPI complexes newly introduced in protein-protein docking benchmark dataset version 4.0 (abbreviated as DBD 4.0[1]) . Here the complex column records the PDB PPI Complex ID. For example, 1FCC is the PDB ID. It has three protein chains: A, B, C. "AB:C" means that there could only be 2 possible PPI cases, which are A:C and B:C. Note that "1ML0_AB:D" had been changed to "1ML0_A:D" due to its update in PDB[5]. This dataset was the testing data in our experiments.

Case	Complex ID	No. of +ve R2R-I	No. of –ve R2R-I	Total
1	1CLV-A:I	23	15017	15040
2	1FLE-E:I	20	10743	10763
3	1GL1-A:I	21	8105	8126
4	1GXD-A:C	24	119784	119808
5	1JTG-B:A	20	43210	43230
6	1OC0-A:B	12	13456	13468
7	1OYV-A:I	37	28459	28496
8	1OYV-B:I	28	28468	28496
9	2ABZ-B:E	12	18048	18060
10	2J0T-A:D	28	19936	19964
11	2OUL-A:B	25	25762	25787

12	3SGQ-E:I	27	9051	9078
13	1FCC-AB:C	16	23056	23072
14	1FFW-A:B	9	8695	8704
15	1H9D-A:B	26	15599	15625
16	1HCF-AB:X	24	24418	24442
17	1JWH-CD:A	15	135729	135744
18	1OFU-A:XY	15	72744	72759
19	1PVH-A:B	20	33949	33969
20	1RV6-VW:X	6	17382	17388
21	1US7-A:B	13	39745	39758
22	1WDW-A:BD	28	185542	185570
23	1XU1-ABD:T	16	16013	16029
24	1ZHH-A:B	23	71529	71552
25	2A5T-A:B	14	75330	75344
26	2A9K-A:B	22	35168	35190
27	2B4J-AB:C	18	22942	22960
28	2FJU-A:B	7	123185	123192
29	2G77-A:B	21	55918	55939
30	2OOR-AB:C	27	128339	128366
31	2VDB-A:B	25	31545	31570
32	3BP8-AB:C	18	57057	57075
33	3D5S-A:C	10	19295	19305
34	1JIW-P:I	24	49326	49350
35	4CPA-A:I	8	11080	11088
36	1LFD-A:B	12	14517	14529
37	1MQ8-A:B	10	32558	32568
38	1R6Q-A:C	20	13328	13348
39	1SYX-A:B	6	8364	8370
40	2AYO-A:B	26	26599	26625
41	2J7P-A:D	37	77343	77380
42	2OZA-B:A	64	112816	112880
43	2Z0E-A:B	30	37140	37170
44	3CPH-A:G	14	77161	77175
45	1F6M-A:C	21	34539	34560
46	1ZLI-A:B	23	22621	22644
47	2O3B-A:B	7	32528	32535
48	1JK9-A:B	37	37142	37179
49	1JZD-AB:C	26	51068	51094
50	1ZM4-A:B	16	170138	170154
51	2I9B-A:E	24	32306	32330
52	2IDO-A:B	20	12955	12975
Total	-	1075	2386748	2387823

4.3. Experimental results

We first validated the R2R-I prediction results of P2K over the first 124 PPI complexes in protein-protein docking benchmark dataset version 4.0 (abbreviated as DBD 4.0[1]) under leave-one-complex-out-alone cross-validation. It should be noted that this subset of PPI complexes is equivalent to protein-protein docking benchmark dataset version 3.0 (abbreviated as DBD 3.0[6]).

As shown in Supplement Table 4.3, P2K has achieved a higher average AUC (0.69078±0.02757) than that of a Random Predictor (0.50000±0.00000). Even when no neighboring residue is considered, the average

AUC (0.59600±0.01531) is still much higher than that of a Random Predictor (0.50000±0.00000). This strongly indicates that the deep knowledge discovered from R2R-C data is effective for R2R-I prediction.

Supplement Table 4.3: A summary of average AUC achieved by P2K for the first 124 PPI complexes in protein-protein docking benchmark dataset version 4.0 (abbreviated as DBD 4.0[1]) under leave-one-complex-alone cross-validation

Method	Average AUC
**Random Predictor**	0.50000±0.00000
**P2K (no. of neighboring residues considered=0; no. of features=18)**	0.59600±0.01531
**P2K (no. of neighboring residues considered=1; no. of features=54)**	0.64293±0.02210
**P2K (no. of neighboring residues considered=2; no. of features=90)**	0.68374±0.02644
**P2K (no. of neighboring residues considered=3; no. of features=126)**	**0.69078±0.02757**

Supplement Table 4.4: A summary of average AUC achieved by P2K (PC projections taking absolute values) for the first 124 PPI complexes in protein-protein docking benchmark dataset version 4.0 (abbreviated as DBD 4.04) under leave-one-complex-alone cross-validation validation using the scikit-learn package 0.18.2[9]

Method	Average AUC
**Random Predictor**	0.50000±0.00000
**P2K (no. of neighboring residues considered=0; no. of features=18)**	0.58340±0.01483
**P2K (no. of neighboring residues considered=1; no. of features=54)**	0.64222±0.02362
**P2K (no. of neighboring residues considered=2; no. of features=90)**	0.66816±0.02800
**P2K (no. of neighboring residues considered=3; no. of features=126)**	**0.68498±0.02841**

We further compared the R2R-I prediction results of P2K with that of PPiPP[3], which is an existing sequence-based R2R-I prediction software that leverages feature engineering over external knowledge of sequences, on the 52 PPI complexes newly introduced in protein-protein docking benchmark dataset version 4.0 (abbreviated as DBD 4.0[1]).

As shown in Supplement Table 4.5, P2K has achieved a higher average AUC (0.64317±0.04159) than that of PPiPP[3] (0.50112±0.00257). All the experimental results are summarized in Supplement Table 4.6. We conducted a two-tailed paired student's t-test between the AUC obtained by PPiPP[3] and those obtained by P2K (no. of neighboring residues considered=3). The p-value was 1.9E-08 < 0.05, demonstrating a strong statistical significance. This indicates that the deep knowledge discovered from R2R-C data is effective for R2R-I prediction. To ensure robustness, experiments were repeated using the machine learning package scikit-learn 0.19.2[9] to obtain the results as shown in Supplement Tables 4.5B and 4.6B. The results show that there is no significant difference between using scikit-learn 0.18.2[9] and scikit-learn 0.19.2[9].

Supplement Table 4.5: A summary of average AUC achieved by P2K compared with PPiPP[3] for the 52 PPI complexes newly introduced in protein-protein docking benchmark dataset version 4.0 (abbreviated as DBD 4.0[1])

Method	Average AUC
**PPiPP[3]**	0.50112±0.00257
**P2K (no. of neighboring residues considered=0; no. of features=18)**	0.55911±0.02502
**P2K (no. of neighboring residues considered=1; no. of features=54)**	0.59947±0.03251

**P2K (no. of neighboring residues considered=2; no. of features=90)**	0.61933±0.04069
**P2K (no. of neighboring residues considered=3; no. of features=126)**	**0.64317±0.04159**

Supplement Table 4.6: A summary of AUC achieved by comparing P2K with PPiPP[3] for the 52 PPI complexes newly introduced in protein-protein docking benchmark dataset version 4.0 (abbreviated as DBD 4.0[1]). Here the complex column records the PDB PPI Complex ID. For example, 1FCC is the PDB ID. It has three protein chains: A, B, C. "AB:C" means that there could only be 2 possible PPI cases, which are A:C and B:C.

Case	Target Complex	PPiPP[3]	P2K (no. of neighboring residues considered=0)	P2K (no. of neighboring residues considered=1)	P2K (no. of neighboring residues considered=2)	P2K (no. of neighboring residues considered=3)
1	1CLV-A:I	0.52033	0.59056	0.65856	**0.72945**	0.71888
2	1FLE-E:I	0.49721	0.51108	**0.74936**	0.68546	0.61433
3	1GL1-A:I	0.49852	0.57080	0.85483	**0.98426**	0.97944
4	1GXD-A:C	0.49835	0.43743	0.43251	0.44369	**0.52648**
5	1JTG-B:A	0.50000	0.51318	0.67009	0.70867	**0.78784**
6	1OC0-A:B	0.49732	0.52438	**0.58052**	0.39270	0.44739A
7	1OYV-A:I	0.49842	0.54008	0.71694	0.85936	**0.91749**
8	1OYV-B:I	0.49842	0.48733	0.86599	0.91204	**0.96417**
9	2ABZ-B:E	0.49922	0.34673	0.64460	**0.66948**	0.58806
10	2J0T-A:D	0.49925	0.54499	0.49113	0.63469	**0.74412**
11	2OUL-A:B	0.51852	0.66274	0.79077	**0.81366**	0.80049
12	3SGQ-E:I	0.49878	0.63456	0.79927	0.91137	**0.91494**
13	1FCC-AB:C	0.49792	0.60940	0.62913	0.64391	**0.84698**
14	1FFW-A:B	0.55067	0.40459	**0.57204**	0.42785	0.53797
15	1H9D-A:B	0.49952	**0.74994**	0.67622	0.68973	0.57144
16	1HCF-AB:X	0.49918	**0.63150**	0.58404	0.52645	0.54721
17	1JWH-CD:A	0.50000	0.49801	**0.51398**	0.47146	0.47581
18	1OFU-A:XY	**0.49652**	0.41427	0.48241	0.43992	0.45319
19	1PVH-A:B	0.49867	**0.61500**	0.43336	0.45353	0.45652
20	1RV6-VW:X	0.49885	0.58095	**0.58982**	0.51694	0.56860
21	1US7-A:B	0.49917	0.41857	0.41923	**0.50205**	0.49915
22	1WDW-A:BD	0.49775	0.52439	0.50726	0.47268	**0.55015**
23	1XU1-ABD:T	0.49953	0.54986	0.63180	**0.63446**	0.53980
24	1ZHH-A:B	0.49853	0.53790	0.47837	0.59516	**0.66359**
25	2A5T-A:B	0.53410	0.50413	**0.70136**	0.53425	0.55422
26	2A9K-A:B	0.49859	0.62750	0.59662	0.65475	**0.72460**
27	2B4J-AB:C	**0.49861**	0.48470	0.46240	0.40889	0.41552
28	2FJU-A:B	0.49870	0.57817	0.76345	**0.82572**	0.80944
29	2G77-A:B	0.49933	0.49318	0.58498	0.61568	**0.65273**
30	2OOR-AB:C	0.49748	0.63837	0.65403	0.62548	**0.74239**
31	2VDB-A:B	0.49786	**0.53400**	0.42251	0.40297	0.37944
32	3BP8-AB:C	0.49774	0.52800	0.54943	**0.63155**	0.59367
33	3D5S-A:C	0.49907	0.52984	0.37385	0.49933	**0.53520**
34	1JIW-P:I	0.50000	0.49976	0.49396	0.45240	**0.51384**
35	4CPA-A:I	0.49928	0.64416	**0.70941**	0.69152	0.67842
36	1LFD-A:B	0.49907	0.74194	0.69927	**0.77309**	0.71291
37	1MQ8-A:B	0.49780	0.54473	0.58913	0.66379	**0.72048**

38	1R6Q-A:C	0.49850	0.56525	0.53570	0.59899	**0.69599**
39	1SYX-A:B	0.49892	0.52092	**0.56476**	0.51930	0.51231
40	2AYO-A:B	0.49883	0.69314	0.62930	**0.73291**	0.69498
41	2J7P-A:D	0.49742	0.58169	0.68831	**0.72076**	0.69356
42	2OZA-B:A	0.50000	0.54907	0.56714	**0.60993**	0.59733
43	2Z0E-A:B	0.49821	0.71387	0.63549	0.75229	**0.78501**
44	3CPH-A:G	0.49813	0.61462	0.72059	0.70454	**0.81235**
45	1F6M-A:C	0.49884	0.66575	0.63045	0.74470	**0.81086**
46	1ZLI-A:B	**0.49901**	0.38683	0.45226	0.42817	0.41485
47	2O3B-A:B	0.49848	0.73537	0.60021	0.68931	**0.75104**
48	1JK9-A:B	0.49915	**0.58864**	0.56552	0.51062	0.56056
49	1JZD-AB:C	0.49933	0.62025	0.70167	**0.79687**	0.75162
50	1ZM4-A:B	0.49780	0.65575	**0.67730**	0.63954	0.66948
51	2I9B-A:E	**0.49861**	0.41848	0.39855	0.36837	0.33519
52	2IDO-A:B	0.49896	0.51730	0.43229	0.49051	**0.61287**
**Average**		0.50112	0.55911	0.59947	0.61933	**0.64317**
**Sample standard deviation**		0.00943	0.09208	0.11961	0.14967	0.15300
**Standard error of the mean**		0.00131	0.01277	0.01659	0.02076	0.02122
**95% upper limit**		0.50369	0.58414	0.63197	0.66001	**0.68476**
**95% lower limit**		0.49856	0.53408	0.56696	0.57865	**0.60158**

Supplement Table 4.5B: A summary of average AUC achieved by P2K compared with PPiPP[3] for the 52 PPI complexes newly introduced in protein-protein docking benchmark dataset version 4.0 (abbreviated as DBD 4.0[1]) using the latest scikit-learn machine package 0.19.2[9]

Method	Average AUC
**PPiPP[3]**	0.50112±0.00257
**P2K (no. of neighboring residues considered=0; no. of features=18)**	0.55575±0.02443
**P2K (no. of neighboring residues considered=1; no. of features=54)**	0.60238±0.03204
**P2K (no. of neighboring residues considered=2; no. of features=90)**	0.61960±0.04176
**P2K (no. of neighboring residues considered=3; no. of features=126)**	**0.63355±0.04496**

Supplement Table 4.6B: A summary of AUC achieved by comparing P2K with PPiPP[3] for the 52 PPI complexes newly introduced in protein-protein docking benchmark dataset version 4.0 (abbreviated as DBD 4.0[1]) using the latest scikit-learn machine package 0.19.2[9]. Here the complex column records the PDB PPI Complex ID. For example, 1FCC is the PDB ID. It has three protein chains: A, B, C. "AB:C" means that there could only be 2 possible PPI cases, which are A:C and B:C.

Case	Target Complex	PPiPP [3]	P2K (no. of neighboring residues considered=0)	P2K (no. of neighboring residues considered=1)	P2K (no. of neighboring residues considered=2)	P2K (no. of neighboring residues considered=3)
1	1CLV-A:I	0.52033	0.63235	0.64041	**0.70660**	0.70304
2	1FLE-E:I	0.49721	0.52337	**0.65304**	0.63988	0.62363
3	1GL1-A:I	0.49852	0.56398	0.87404	0.97914	**0.98534**
4	1GXD-A:C	0.49835	0.44639	0.36237	0.39462	**0.49887**
5	1JTG-B:A	0.50000	0.50949	0.67343	0.76395	**0.77568**
6	1OC0-A:B	0.49732	0.47610	**0.53419**	0.41903	0.43391
7	1OYV-A:I	0.49842	0.55499	0.67183	0.76626	**0.81375**
8	1OYV-B:I	0.49842	0.49896	0.75610	0.83091	**0.88565**

9	2ABZ-B:E	0.49922	0.42161	0.56434	**0.56794**	0.51055
10	2J0T-A:D	0.49925	0.54117	0.51252	0.59374	**0.68240**
11	2OUL-A:B	0.51852	0.63607	0.81443	0.83120	**0.86081**
12	3SGQ-E:I	0.49878	0.64695	0.82360	0.89937	**0.91952**
13	1FCC-AB:C	0.49792	0.57231	0.59637	0.62298	**0.73582**
14	1FFW-A:B	0.55067	0.33600	**0.57179**	0.53358	0.56803
15	1H9D-A:B	0.49952	**0.74160**	0.73151	0.70993	0.67343
16	1HCF-AB:X	0.49918	**0.61613**	0.58934	0.52432	0.54001
17	1JWH-CD:A	0.50000	0.51694	**0.55150**	0.44586	0.44081
18	1OFU-A:XY	**0.49652**	0.40087	0.46489	0.42988	0.45644
19	1PVH-A:B	0.49867	**0.63730**	0.48873	0.39739	0.40448
20	1RV6-VW:X	0.49885	**0.59772**	0.45272	0.57755	0.52910
21	1US7-A:B	**0.49917**	0.39880	0.38640	0.48351	0.36237
22	1WDW-A:BD	0.49775	0.55049	0.52811	0.55891	**0.57345**
23	1XU1-ABD:T	0.49953	0.55060	0.64045	0.64395	**0.64574**
24	1ZHH-A:B	0.49853	0.55501	0.50760	0.56477	**0.61843**
25	2A5T-A:B	0.53410	0.50603	**0.70897**	0.60072	0.57084
26	2A9K-A:B	0.49859	0.55290	0.60284	0.62070	**0.63124**
27	2B4J-AB:C	**0.49861**	0.44975	0.47125	0.38222	0.42300
28	2FJU-A:B	0.49870	0.60988	0.75470	0.78204	**0.81193**
29	2G77-A:B	0.49933	0.50604	0.60398	0.65078	**0.65469**
30	2OOR-AB:C	0.49748	0.60294	0.62638	0.64771	**0.66013**
31	2VDB-A:B	**0.49786**	0.48053	0.38994	0.32328	0.39301
32	3BP8-AB:C	0.49774	0.54051	0.54191	**0.61419**	0.59609
33	3D5S-A:C	0.49907	0.52921	**0.60534**	0.49985	0.46580
34	1JIW-P:I	0.50000	0.52038	**0.53019**	0.44877	0.45147
35	4CPA-A:I	0.49928	0.61581	**0.78799**	0.77128	0.76603
36	1LFD-A:B	0.49907	0.74763	0.69938	0.77222	**0.77443**
37	1MQ8-A:B	0.49780	0.62459	0.68399	0.80133	**0.89053**
38	1R6Q-A:C	0.49850	0.58104	0.58588	0.56224	**0.58772**
39	1SYX-A:B	0.49892	0.47131	**0.56029**	0.48458	0.43441
40	2AYO-A:B	0.49883	0.65219	0.61584	0.70557	**0.70757**
41	2J7P-A:D	0.49742	0.58555	0.69361	**0.74392**	0.70802
42	2OZA-B:A	0.50000	0.57730	0.57243	**0.58985**	0.57065
43	2Z0E-A:B	0.49821	0.70596	0.68914	0.78765	**0.82130**
44	3CPH-A:G	0.49813	0.58253	0.65186	0.75772	**0.85609**
45	1F6M-A:C	0.49884	0.64422	0.70487	0.79904	**0.80091**
46	1ZLI-A:B	**0.49901**	0.39996	0.34261	0.41172	0.39191
47	2O3B-A:B	0.49848	0.75582	0.64599	0.67687	**0.77746**
48	1JK9-A:B	0.49915	**0.57992**	0.53564	0.47523	0.50732
49	1JZD-AB:C	0.49933	0.58420	0.67491	0.79349	**0.81359**
50	1ZM4-A:B	0.49780	0.59424	0.65329	0.71170	**0.74033**
51	2I9B-A:E	**0.49861**	0.45237	0.46076	0.36731	0.33694
52	2IDO-A:B	0.49896	0.52106	0.53984	0.55201	**0.55975**
**Average**		0.50112	0.55575	0.60238	0.61960	**0.63355**
**Sample standard deviation**		0.00943	0.08987	0.11788	0.15364	0.16541
**Standard error of the mean**		0.00131	0.01246	0.01635	0.02131	0.02294
**95% upper limit**		0.50369	0.58018	0.63442	0.66136	**0.67851**
**95% lower limit**		0.49856	0.53132	0.57034	0.57784	**0.58859**

4.4. Case Study

To demonstrate the effectiveness of P2K for illustration purpose, we conducted a case study on the target complex 1GL1-A:I, which is one of the 52 PPI complexes newly introduced in protein-protein docking benchmark dataset version 4.0 (abbreviated as DBD 4.0[1]). This target complex has the PDB[5] ID 1GL1, which describes the PPI between bovine alpha-chymotrypsin and PMP-C, an inhibitor from the insect Locusta migratoria. According to DBD 4.0[1], R2R-I occurs on protein sequence chains A and I. Following the procedure mentioned, we trained P2K over the first 124 PPI complexes in DBD 4.0[1] under default parameter setting except setting no. of neighboring residues considered=3. Also, the latest scikit-learn machine learning package 0.19.2[9] was adopted. We then applied P2K to predict R2R-I between the chains A and I of the target complex 1GLI. A working practice of an experimental biologist is to focus on the top predictions. Hence, in this experiment, only the top 5 positive predictions remained and the other positive predictions were forced to be negative predictions. As shown in Supplement Table 4.7, P2K outperformed PPiPP[3], where P2K had a precision of 80% among the top 5 predictions while PPiPP[3] had a precision of 0% among the top 5 predictions. Fig. S4.2 provides a 3D illustration of the predictions.

Supplement Table 4.7: Precision, Recall, Specificity, F-Measure achieved by P2K comparing with PPiPP[3] on the target complex 1GL1-A:I on DBD 4.0[1], where in each case only the top 5 positive predictions remained and the other positive predictions were forced to be negative predictions.

	TP	FP	TN	FN	Precision	Recall	Specificity	F1
P2K	4	1	8104	17	0.80000	0.19048	0.99988	0.30769
PPiPP[3]	0	5	8100	21	0.00000	0.00000	0.99938	0.00000

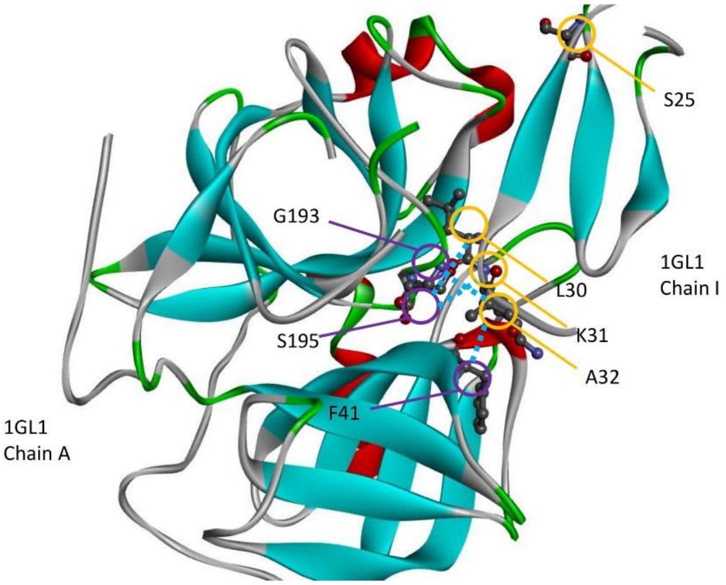

Figure. S4.2. A demonstration of the R2R-I prediction between protein sequence chains A and I on the target complex 1GL1. The residues on protein chain A are enclosed by purple circles while the residues on the protein sequence chain I are enclosed by yellow circles. The 4 out of 5 true positive predictions of P2K are shown in the figure and are connected by blue dash lines. They are 1: (S195 on chain A, K31 on chain I), 2: (S195 on chain A, L30 on chain I), 3: (F41 on chain A, A32 on chain I) and 4: (F41 on chain A, A32 on chain I), while the false positive prediction is (S195 on chain A, S25 on chain I). As shown in Supplement Table 4.7, P2K outperformed PPiPP[3], where P2K had a precision of 80% among the top 5 predictions while PPiPP [3] had a precision of 0% among the top 5 predictions.

4.5. Discussion

From the summary of prediction results for the 52 non-redundant PPI complexes introduced in DBD 4.0[1]) shown in Supplement Tables 4.5 and 4.6, we can now positively report that deep knowledge discovered from R2R contact data, i.e. PCs and RSRVs, are useful for constructing feature vectors to train machine learning models for sequence-based R2R-I prediction.

It is interesting to observe that the use of deep knowledge has incrementally improved the predictive performance when more neighboring residues are considered. Also, as shown in Supplement Table 4.4, there are 5, 9, 13, and 21 best prediction cases obtained by the R2R-I predictor constructed through the inclusion of 0, 1, 2 and 3 of neighboring residues, respectively. This indicates that the statistics of the subtle R2R-I physiochemical environment have been acquired. In aggregation, P2K has obtained 48 best prediction cases out of 52 cases analyzed. We will explore the combination of these different R2R-I predictors for R2R-I prediction in the near future.

An interesting observation here is that the improvement of AUC did not absolutely follow the number of neighboring residue pairs considered in the FV construction (Supplement Tables 4.5 and 4.6). As for the top prediction cases among the 52 cases, PPiPP[3] attained 4 cases and P2K attained 48 cases. Among the 48 cases, when the number of neighboring residue pairs considered increased from 0, 1, 2, and 3, the number of highest AUC obtained by P2K was 5, 9, 13, and 21 respectively. Such results indicate that intriguing stereo-physiochemical environment in R2R-I regions varies. It shows that, in some cases, the use of more neighboring residue pairs may not help. However, when all of them are used together as an ensemble, we believe that the prediction rate could be further improved. We include this in our plan when additional information from functional conserved regions are used to further refining the search and improve the prediction rate.

4.6. References in Supplement Note 4

1. Hwang, H., Vreven, T., Janin, J. & Weng, Z. Protein-protein docking benchmark version 4.0. *Proteins Struct. Funct. Bioinforma.* **78,** 3111–3114 (2010).
2. Glaser, F., Steinberg, D. M., Vakser, I. A. & Ben-Tal, N. Residue frequencies and pairing preferences at protein-protein interfaces. *Proteins Struct. Funct. Genet.* **43,** 89–102 (2001).
3. Ahmad, S. & Mizuguchi, K. Partner-aware prediction of interacting residues in protein-protein complexes from sequence data. *PLoS One* **6,** e29104 (2011).
4. Afsar Minhas, F. ul A., Geiss, B. J., Ben-Hur, A. & Minhas, F. U. A. A. PAIRpred: partner-specific prediction of interacting residues from sequence and structure. *Proteins* **82,** 1142–55 (2014).
5. Berman, H. M. *et al.* The Protein Data Bank. *Nucleic Acids Res.* **28,** 235–242 (2000).
6. Hwang, H., Pierce, B., Mintseris, J., Janin, J. & Weng, Z. Protein-protein docking benchmark version 3.0. *Proteins* **73,** 705–9 (2008).
7. POWERS, D. M. W. Evaluation: From Precision, Recall and F-Measure To Roc, Informedness, Markedness & Correlation. *J. Mach. Learn. Technol.* **2,** 37–63 (2011).
8. Geurts, P., Ernst, D. & Wehenkel, L. Extremely randomized trees. *Mach. Learn.* **63,** 3–42 (2006).
9. Pedregosa, F., Varoquaux, G. & Gramfort, A. Scikit-learn: Machine learning in Python. *J. Mach.* (2011).
